# The STOP COVID 2 Study: Fluvoxamine vs Placebo for Outpatients With Symptomatic COVID-19, a Fully Remote Randomized Controlled Trial

**DOI:** 10.1093/ofid/ofad419

**Published:** 2023-08-08

**Authors:** Angela M Reiersen, Caline Mattar, Rachel A Bender Ignacio, David R Boulware, Todd C Lee, Rachel Hess, Alexander J Lankowski, Emily G McDonald, J Philip Miller, William G Powderly, Matthew F Pullen, Jeffrey T Rado, Michael W Rich, Joshua T Schiffer, Julie Schweiger, Adam M Spivak, Angela Stevens, Simone N Vigod, Payal Agarwal, Lei Yang, Michael Yingling, Torie R Gettinger, Charles F Zorumski, Eric J Lenze

**Affiliations:** Department of Psychiatry, Washington University School of Medicine, St. Louis, Missouri, USA; Division of Infectious Diseases, Department of Internal Medicine, Washington University School of Medicine, St. Louis, Missouri, USA; Vaccine and Infectious Disease Division, Fred Hutchinson Cancer Center, Seattle, Washington, USA; Allergy & Infectious Diseases Division, Department of Medicine, University of Washington, Seattle, Washington, USA; Division of Infectious Diseases and International Medicine, University of Minnesota, Minneapolis, Minnesota, USA; Division of Infectious Diseases, Department of Medicine, McGill University Health Centre, Montréal, Québec, Canada; Department of Medicine, Clinical Practice Assessment Unit, McGill University Health Centre, Montréal, Québec, Canada; Division of Experimental Medicine, Department of Medicine, McGill University, Montréal, Québec, Canada; Division of Health System Innovation and Research, University of Utah, Salt Lake City, Utah, USA; Division of General Internal Medicine, University of Utah, Salt Lake City, Utah, USA; Vaccine and Infectious Disease Division, Fred Hutchinson Cancer Center, Seattle, Washington, USA; Department of Medicine, Clinical Practice Assessment Unit, McGill University Health Centre, Montréal, Québec, Canada; Division of Experimental Medicine, Department of Medicine, McGill University, Montréal, Québec, Canada; Division of General Internal Medicine, Department of Medicine, McGill University Health Centre, Montréal, Québec, Canada; Institute for Informatics, Data Science and Biostatistics, Washington University School of Medicine, St. Louis, Missouri, USA; Division of Infectious Diseases, Department of Internal Medicine, Washington University School of Medicine, St. Louis, Missouri, USA; Division of Infectious Diseases and International Medicine, University of Minnesota, Minneapolis, Minnesota, USA; Departments of Psychiatry & Behavioral Sciences and Medicine, Northwestern University Feinberg School of Medicine, Chicago, Illinois, USA; Department of Medicine, Cardiovascular Division, Washington University School of Medicine, St. Louis, Missouri, USA; Vaccine and Infectious Disease Division, Fred Hutchinson Cancer Center, Seattle, Washington, USA; Allergy & Infectious Diseases Division, Department of Medicine, University of Washington, Seattle, Washington, USA; Department of Psychiatry, Washington University School of Medicine, St. Louis, Missouri, USA; Division of Infectious Diseases, University of Utah, Salt Lake City, Utah, USA; Department of Psychiatry, Washington University School of Medicine, St. Louis, Missouri, USA; Department of Psychiatry, Temerty Faculty of Medicine, University of Toronto and Women's College Hospital, Toronto, Ontario, Canada; Department of Family and Community Medicine, Temerty Faculty of Medicine, University of Toronto and Women's College Hospital, Toronto, Ontario, Canada; Department of Psychiatry, Washington University School of Medicine, St. Louis, Missouri, USA; Department of Psychiatry, Washington University School of Medicine, St. Louis, Missouri, USA; Department of Psychiatry, Washington University School of Medicine, St. Louis, Missouri, USA; Department of Psychiatry, Washington University School of Medicine, St. Louis, Missouri, USA; Department of Psychiatry, Washington University School of Medicine, St. Louis, Missouri, USA; Department of Anesthesiology, Washington University School of Medicine, St. Louis, Missouri, USA

**Keywords:** COVID-19, clinical trial, fluvoxamine, fully remote, sigma1 receptor agonist

## Abstract

**Background:**

Prior randomized clinical trials have reported benefit of fluvoxamine ≥200 mg/d vs placebo for patients infected with severe acute respiratory syndrome coronavirus 2 (SARS-CoV-2).

**Methods:**

This randomized, double-blind, placebo-controlled, fully remote multisite clinical trial evaluated whether fluvoxamine prevents clinical deterioration in higher-risk outpatients with acute coronavirus disease 2019 (COVID-19). Between December 2020 and May 2021, nonhospitalized US and Canadian participants with confirmed symptomatic infection received fluvoxamine (50 mg on day 1, 100 mg twice daily thereafter) or placebo for 15 days. The primary modified intent-to-treat (mITT) population included participants who started the intervention within 7 days of symptom onset with a baseline oxygen saturation ≥92%. The primary outcome was clinical deterioration within 15 days of randomization, defined as having both (1) shortness of breath (severity ≥4 on a 0–10 scale or requiring hospitalization) *and* (2) oxygen saturation <92% on room air or need for supplemental oxygen.

**Results:**

A total of 547 participants were randomized and met mITT criteria (n = 272 fluvoxamine, n = 275 placebo). The Data Safety Monitoring Board recommended stopping early for futility related to lower-than-predicted event rates and declining accrual concurrent with vaccine availability in the United States and Canada. Clinical deterioration occurred in 13 (4.8%) participants in the fluvoxamine group and 15 (5.5%) participants in the placebo group (absolute difference at day 15, 0.68%; 95% CI, −3.0% to 4.4%; log-rank *P* = .91).

**Conclusions:**

This trial did not find fluvoxamine efficacious in preventing clinical deterioration in unvaccinated outpatients with symptomatic COVID-19. It was stopped early and underpowered due to low primary outcome rates.

**Clinical Trials Registration:**

ClinicalTrials.gov Identifier: NCT04668950.

Severe acute respiratory syndrome coronavirus 2 (SARS-CoV-2) infection causes coronavirus disease 2019 (COVID-19), which can result in a systemic hyperinflammatory response associated with clinical deterioration, most often in nonvaccinated persons of advanced age or with medical comorbidities such as obesity or diabetes [1]. Early outpatient treatment with antiviral or anti-inflammatory agents has the potential to prevent clinical deterioration [2]. In 2019, it was shown that early initiation of fluvoxamine, a selective serotonin reuptake inhibitor (SSRI), sigma-1 receptor (S1R) agonist [3], and functional inhibitor of acid sphingomyelinase (FIASMA) [4] reduced deterioration and mortality in animal models of sepsis [3]. Fluvoxamine has advantages for drug repurposing, including ease of use, high safety margin, and low cost [5].

In the STOP COVID 1 trial in 2020 [5], which compared fluvoxamine 100 mg 3 times daily vs placebo in 152 outpatients with early symptomatic COVID-19, clinical deterioration occurred in 0 of 80 patients in the fluvoxamine group vs 6 of 72 patients in the placebo group (absolute difference, 8.7%; 95% CI, 1.8% to 16.4%; *P* = .009). We designed STOP COVID 2 to replicate the findings of STOP COVID 1 in a higher-risk population and using a slightly lower dose (maximum total daily dose of 200 mg vs 300 mg).

## METHODS

STOP COVID-2 was a double-blind, placebo-controlled, randomized fully remote [6] clinical trial comparing fluvoxamine with placebo for treatment of outpatients within 7 days of COVID-19 symptom onset with a baseline oxygen saturation (SpO_2_) ≥92% on room air. The protocol and statistical analysis plan are in Supplementary Data 1.

### Patient Consent

Participants provided written informed consent (typically electronically). The design of the work, including consenting procedures, was approved by local ethical committees: The Institutional Review Board at Washington University in St. Louis approved trial conduct at US sites. The Research Institute of the McGill University Health Centre and Women's College Hospital Research Ethics Boards and Health Canada approved trial conduct at Canadian sites.

### Study Design

Participants in the United States and Canada were enrolled from December 22, 2020, to May 21, 2021. The study recruited locally from study sites in St. Louis, Missouri, Chicago, Illinois, Salt Lake City, Utah, and Seattle, Washington, as well as nationally in the United States and across Quebec and Ontario, Canada, from sites centered in Montreal and Toronto, respectively. Participants were recruited via electronic health records, referral from health care professionals and SARS-CoV-2 testing sites, advertisements on social media and the internet, a study website, and communication via television and news articles.

Potential participants underwent screening through electronic communication and/or telephone and provided written informed consent (typically electronically). After consent, participants were randomized and sent a study package by overnight mail or courier. The study package included a blinded container of study medication, an oxygen saturation monitor, an automated blood pressure monitor (for participants in the United States), and a thermometer. Participants were instructed to use this equipment to assess their vital signs at baseline (immediately upon receipt of the package), then twice daily for 15 days while taking the study medication. During this time, participants received a survey link each morning and evening via email or text message prompting them to record their vital signs, as well as self-reported medication adherence and COVID-19 symptoms. Study staff reviewed baseline data to reconfirm eligibility after receipt of the package and then continued to monitor progress remotely on a daily basis. Participants with SpO_2_ <92% and dyspnea severity ≥4 (scale of 0 to 10) at baseline or during trial conduct were guided to seek urgent local medical attention, with oversight from study clinicians. At the end of 15 days, participants completed end of active treatment measures. Final follow-up measures were collected at 90 days postrandomization. All study measures were collected using REDCap, and surveys were available in English, French, and Spanish, per participant preference and site availability. The prespecified primary outcome from the 15-day trial is reported in this manuscript.

### Participants

**Figure 1.**
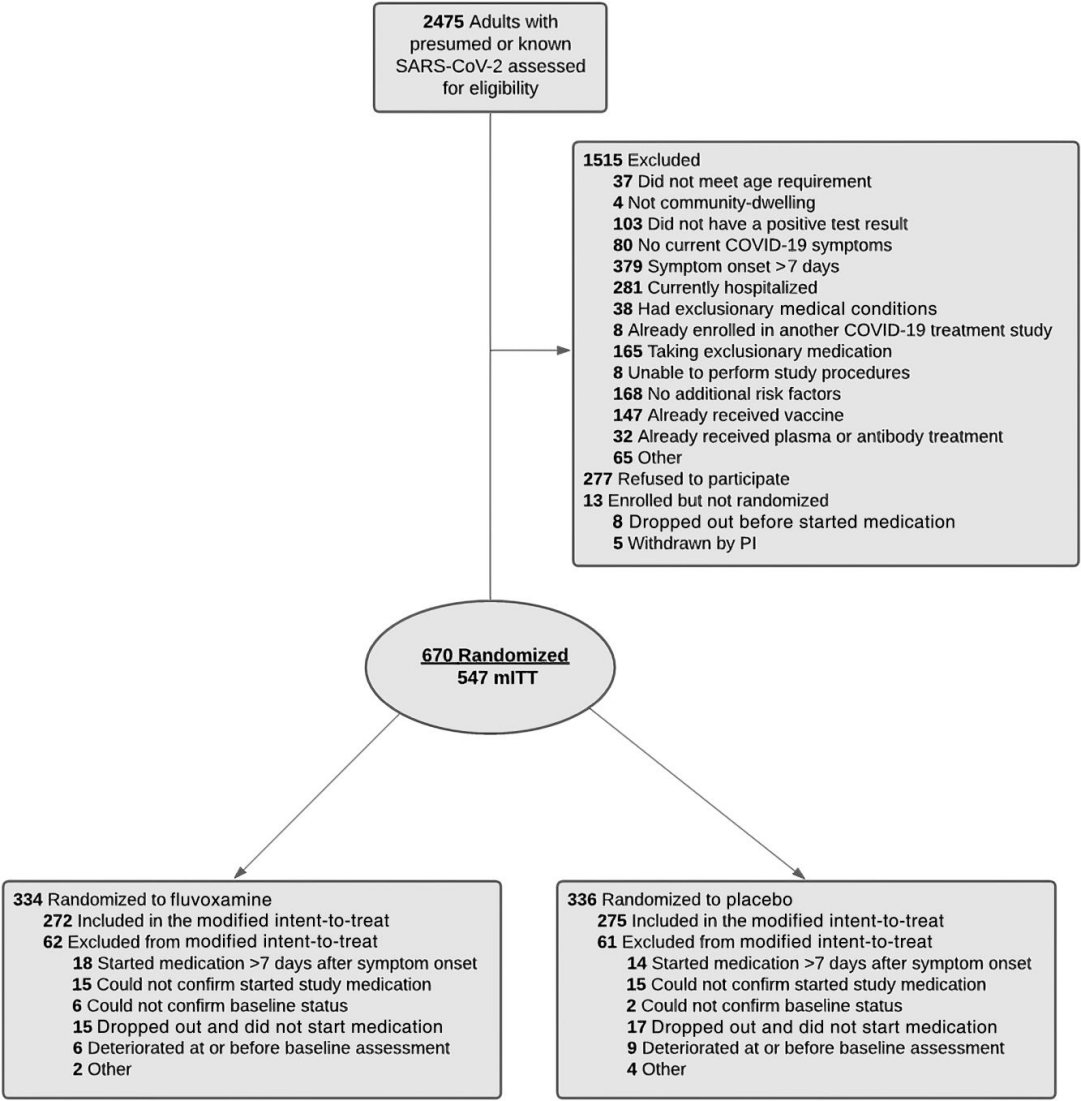
CONSORT flow diagram. Abbreviations: COVID-19, coronavirus disease 2019; mITT, modified intent-to-treat; SARS-CoV-2, severe acute respiratory syndrome coronavirus 2.

The CONSORT diagram is shown in Figure 1. The study included community-dwelling, unvaccinated adults aged ≥30 years with SARS-CoV-2 infection demonstrated by polymerase chain reaction (PCR) assay (confirmed by participant self-report, with documentation when feasible). Participants had to be symptomatic with at least 1 of the following at the time of randomization: fever, cough, myalgia, mild dyspnea, chest pain, diarrhea, nausea, vomiting, anosmia (inability to smell), ageusia (inability to taste), sore throat, nasal congestion. Participants completed a baseline survey in which they self-reported severity of symptoms (scale of 0–10, with 0 indicating never had symptoms and 10 indicating severe symptoms). Onset of symptoms had to be ≤7 days before the date of a participant's first anticipated study medication dose. In addition, participants had to have at least 1 of the following risk factors for COVID-19 progression per self-report: age ≥40; identifying as Black/African American, Hispanic, South Asian, or Native American/Indigenous Canadian (participants could report ≥1 race); or a diagnosis of at least 1 of the following medical conditions: obesity (body mass index >30 kg/m^2^), hypertension, diabetes, heart disease, lung disease (eg, moderate–severe asthma, chronic obstructive pulmonary disease), or immune disorder (eg, rheumatoid arthritis). Exclusion criteria were oxygen saturation <92% on room air or otherwise requiring hospitalization at or before the time of the first medication dose, highly unstable medical comorbidity, taking a medication that could not be co-prescribed with fluvoxamine, or known bipolar disorder (see Supplementary Methods 1 for full inclusion and exclusion criteria).

The primary analysis population was defined as a modified intent-to-treat (mITT) population, including all participants who took at least 1 dose of study drug within 7 days of symptom onset and who did not already meet the primary end point at the time of baseline assessment. If participants met clinical deterioration criteria at baseline or took the first medication dose after symptom day 7, they were not included in the mITT analysis. This mITT definition was necessitated by the contactless nature of the trial, in which clinical status could not be confirmed until receipt of the study materials, and it was expected that some proportion of patients would either not receive their package and thus not initiate study procedures until after the seventh day if at all or would already meet end point criteria on the baseline vital sign assessment.

### Randomization

Participants were randomized 1:1 to fluvoxamine or matching placebo. Randomization schedules were stratified by site, sex, and age group (<40 and ≥40 years). Treatments were randomly allocated using alternating blocks of sizes 2 and 4, with allocation conducted via REDCap. Research staff and participants were both blinded to treatment assignment.

### Intervention

The active intervention was fluvoxamine 100 mg twice daily for 15 days (starting on day 1 at 50 mg with a titration of 100 mg twice daily). The control intervention was a placebo that was formulated to look identical to the fluvoxamine capsules. Participants were instructed to take a first dose of study medication immediately after receiving their study supplies and completing baseline assessments (ie, oxygen saturation, blood pressure, heart rate, completion of online symptom questionnaire). Study staff contacted participants to confirm they had started the study medication and to instruct them to increase the dose to 100 mg twice daily (or matching placebo) on the second study day, if tolerated. The dose could be reduced (or the medication stopped) in consultation with a study clinician if intolerable adverse effects developed (eg, nausea, dizziness). Study staff contacted participants if concerning vital signs were reported to determine whether additional clinical intervention was required, with oversight by study clinicians.

### Outcomes

The main prespecified primary outcome was clinical deterioration through 15 days postrandomization, defined as both (1) the presence of dyspnea (score of ≥4 on a 0–10 severity scale) and/or hospitalization for shortness of breath or pneumonia *and* (2) decrease in oxygen saturation (<92% on room air) and/or requirement for supplemental oxygen to keep oxygen saturation ≥92% (Supplementary Methods 2). To enable direct comparison with outcomes from other COVID-19 trials, a second prespecified primary outcome for clinical deterioration was defined as the peak disease severity at any point during the 15 days postrandomization, as measured by a modified version of the World Health Organization (WHO) Therapeutic Trial Synopsis 9-point scale. Additional details are available in Supplementary Methods 2.

Outcomes were captured in the REDCap surveys, and clinical deterioration was also sometimes captured by direct communication with participants, or by medical record review (when available) if participants did not complete surveys through day 15. Participants remained in the study through day 90, when a final symptom questionnaire was completed and interval adverse events were assessed.

### Statistical Analyses

The sample size target was 880 participants in the mITT sample. This sample size calculation assumed an overall 16.5% event rate for the primary outcome of clinical deterioration. With an enriched sample (ie, those more likely to deteriorate), we expected to have a 20% rate of deterioration in the placebo group. If this prediction was accurate, a sample size of 880 in the mITT sample would give 80% power to detect a treatment effect of 35% reduction (20% in placebo vs 13% in fluvoxamine).

The a priori conditional power for futility was set at 10%. A preplanned interim analysis was conducted once the trial had recruited >50% of the planned number of participants.

The primary outcome was evaluated using a survival analysis, which censored participants on the day that they met the primary outcome or on the last day they completed an outcome assessment. Difference in outcomes was assessed by a log-rank test with stratification for the randomization stratification variables of site, sex, and age group.

The modified WHO COVID-19 Therapeutic Trial Synopsis 9-point scale was compared between the 2 groups using a *t* test. Each participant's score reflected their maximum rating for the 15 days of the trial. The numbers of participants at each level of the scale were compared using a Fisher exact test. A per-protocol analysis was conducted, which included participants who took ≥80% of expected doses of study medication until the time of reporting deterioration or completion of planned 15-day treatment. Adverse events were also analyzed by treatment arm. All analyses were performed in SAS, version 9.4 (SAS Institute, Cary, NC, USA), and R, version 4.1.2 (The R Foundation for Statistical Computing, Vienna, Austria).

## RESULTS

### Recruitment and Early Stopping for Futility

The first participant was enrolled on December 22, 2020. As of May 5, 2021, 527 of the 880 planned mITT participants had been randomized. At that time, the total observed event rate was only 5% (vs 16.5% original assumed rate). Therefore, the Data Safety Monitoring Board (DSMB) authorized the statistician (J.P.M.) to be unblinded; the statistician recalculated conditional power as 5% using an updated assumption with an event rate of 8% for the placebo group and 4% for the treatment group and the original sample size. This met the a priori stopping condition for futility, and the DSMB and study team determined it would be infeasible to increase the sample size to several thousand individuals, which would have been necessary for adequate power (Supplementary Results 1). Therefore, the study was stopped early; recruitment ended May 21, 2021. Enrolled participants remained in the trial through completion of planned follow-up.

### Participant Characteristics

Of 2475 individuals screened, 670 (27.1%) were eligible and randomized to receive the study intervention. Of these, 547 (81.6%) remained eligible for inclusion in the mITT population at the time of study material receipt and baseline assessment; a similar number in each arm (62 and 61) was excluded, due to not receiving and starting the study medication until >7 days after symptom onset (n = 32), inability to confirm baseline status and/or medication initiation (n = 38), withdrawal before medication initiation (n = 32), or already meeting clinical deterioration criteria at baseline (n = 15) (Figure 1).

Participant demographics and clinical characteristics at baseline are shown in Table 1. Nearly two-thirds (62%) were women, and 27% of participants self-identified as non-White; 13% identified as Hispanic. The median age of participants (interquartile range [IQR]) was 47 (41–55) years. The median duration of COVID-19 symptoms at time of first medication dose (IQR, range) was 5 (4–6, 1–7) days for both groups in the mITT population. The baseline oxygen saturation was also the same for both groups (median [IQR], of 97% [96%–98%]).

**Table 1.** Baseline Characteristics of Modified Intention-to-Treat Population Participants

Characteristics	Fluvoxamine (n = 272)	Placebo (n = 275)
Age, y		
Median (IQR)	47 (40–55)	48 (41–56)
Range	25–83	30–78
Sex at birth, No. (%)^a^		
Male	103 (37.9)	105 (38.2)
Female	169 (62.1)	170 (61.8)
Race, No. (%)^b,c^		
American Indian/Alaska Native	6 (2.2)	8 (2.9)
Asian	8 (2.9)	5 (1.8)
Black/African American	22 (8.1)	23 (8.4)
White/Caucasian	197 (72.4)	201 (73.1)
Native Hawaiian/Pacific Islander	4 (1.5)	5 (1.8)
South Asian	3 (1.1)	2 (0.7)
Unknown/not reported	18 (6.6)	22 (8.0)
Other	25 (9.2)	20 (7.3)
Ethnicity, No. (%)^b^		
Hispanic/Latino	35 (12.9)	37 (13.5)
Non-Hispanic/Non-Latino	234 (86.0)	236 (85.8)
Unknown/not reported	3 (1.1)	2 (0.7)
Oxygen saturation, %		
Median (IQR)	97 (96–98)	97 (96–98)
Duration of COVID-19 Symptoms, d^b^		
Median (IQR)	5 (4–6)	5 (4–6)
Body mass index category, No. (%)		
BMI <25 kg/m^2^	71 (26.1)	62 (22.5)
BMI 25–29.9 kg/m^2^	86 (31.6)	90 (32.7)
BMI ≥30 kg/m^2^	115 (42.3)	123 (44.7)
Coexisting conditions, No. (%)^b^		
Heart disease	4 (1.5)	4 (1.5)
Lung disease	2 (0.7)	2 (0.7)
Liver disease	1 (0.4)	1 (0.4)
Kidney disease	1 (0.4)	2 (0.7)
Hepatitis B/C	1 (0.4)	1 (0.4)
Immune disorders	14 (5.1)	4 (1.5)
HIV	1 (0.4)	4 (1.5)
Asthma	40 (14.7)	33 (12.0)
Hypertension	55 (20.2)	62 (22.5)
Diabetes	23 (8.5)	28 (10.2)
Active cancer	0 (0)	1 (0.4)
Thyroid problems	20 (7.4)	27 (9.8)
Other medical conditions	42 (15.4)	54 (19.6)
Most severe COVID-19 symptom at baseline, No (%)^b^		
Loss of smell	74 (27.2)	91 (33.1)
Fatigue	70 (25.7)	59 (21.5)
Loss of taste	47 (17.3)	48 (17.5)
Nasal congestion	45 (16.5)	30 (10.9)
Cough	34 (12.5)	37 (13.5)
Body aches	27 (9.9)	30 (10.9)
Loss of appetite	21 (7.7)	26 (9.5)
Subjective fever	12 (4.4)	18 (6.5)
Nausea	11 (4.0)	4 (1.5)
Chills	9 (3.3)	10 (3.6)
Diarrhea	8 (2.9)	9 (3.3)
Sore throat	8 (2.9)	7 (2.5)
Shortness breath	6 (2.2)	7 (2.5)

Abbreviations: BMI, body mass index; COVID-19, coronavirus disease 2019; IQR, interquartile range.

a Only 1 participant reported self-identified gender as different from sex assigned at birth.

b Per participant self-report.

c Numbers do not add up to total No. as participants were permitted to select more than 1 race.

### Efficacy of Fluvoxamine vs Placebo

**Figure 2.**
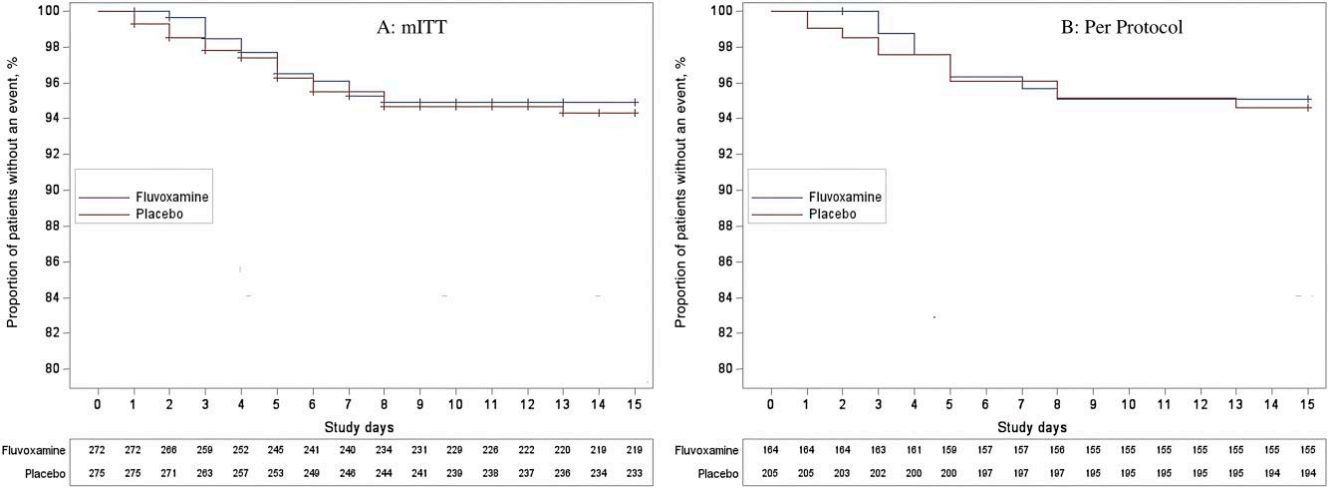
Time to clinical deterioration in the fluvoxamine and placebo groups. *A*, Time to clinical deterioration: modified intention-to-treat analysis. *B*, Time to clinical deterioration: per-protocol analysis. The x-axis indicates trial study days. Study day 0 represents the day of randomization. The y-axis is the percentage of patients free from clinical deterioration.

Detailed results of prespecified outcome measures are shown in Table 2. Clinical deterioration (by our 2-part study definition) occurred in 13 (4.8%) of 272 patients in the fluvoxamine arm and 15 (5.5%) of 275 patients in the placebo arm (absolute difference at day 15, 0.68%; 95% CI, −3.0% to 4.4%; log-rank *P* = .91) (Figure 2*A*).

**Table 2.** Primary Outcome

	Fluvoxamine	Placebo		
Primary End Points	(n = 272)	(n = 275)	Absolute Difference (95% CI)	*P* Value
Clinical deterioration, mITT, No. (%)	13 (4.8)	15 (5.5)	0.68 (−3.00 to 4.40)	.91^a^
WHO 9-point scale, mITT^b^				
1. Ambulatory, no activity limitation	259 (95.2)	259 (94.2)	…	.70
2. Ambulatory, activity limitation	4 (1.5)	6 (2.2)	…	.75
3. Hospitalization, no supplemental oxygen	2 (0.7)	1 (0.4)	…	.62
4. Hospitalization, oxygen needed	6 (2.2)	9 (3.3)	…	.60
5. Non-invasive ventilation	1 (0.4)	0 (0.0)	…	.50
6. Intubation & mechanical ventilation	0 (0.0)	0 (0.0)	…	1.00
7. IMV plus organ support needed	0 (0.0)	0 (0.0)	…	1.00
8. Death	0 (0.0)	0 (0.0)	…	1.00
WHO 9-point scale, mean (SD)^c^	1.11 (0.54)	1.13 (0.56)	0.02 (−0.07 to 0.11)	.72
Per-protocol analysis (exploratory)	(n = 164, 60.3%)	(n = 205, 74.5%)	**…**	**…**
Clinical deterioration, per-protocol, No. (%)	8 (4.9)	11 (5.4)	0.46 (−4.03 to 5.03)	.92^a^
WHO 9-point scale, per-protocol^b^				
1. Ambulatory, no activity limitation	156 (95.1)	194 (94.6)	…	1.00
2. Ambulatory, activity limitation	3 (1.8)	5 (2.4)	…	.74
3. Hospitalization, no supplemental oxygen	2 (1.2)	0 (0.0)	…	.20
4. Hospitalization, oxygen needed	3 (1.8)	6 (2.9)	…	.74
5. Noninvasive ventilation	0 (0.0)	0 (0.0)	…	1.00
6. Intubation & mechanical ventilation	0 (0.0)	0 (0.0)	…	1.00
7. IMV plus organ support needed	0 (0.0)	0 (0.0)	…	1.00
8. Death	0 (0.0)	0 (0.0)	…	1.00
WHO 9-point scale, mean (SD)^c^	1.10 (0.47)	1.11 (0.53)	0.01 (−0.09 to 0.11)	.78

Abbreviations: IMV, intubation and mechanical ventilation; mITT, modified intention to treat; WHO, World Health Organization.

a *P* values computed from the stratified log-rank test.

b *P* values computed from a Fisher exact test.

c *P* values computed from a *t* test.

When analyzed as a continuous score, mean WHO scale peak severity was not statistically significant between treatment groups. When the WHO scale was instead analyzed categorically, it was noted that a higher number of individuals in the placebo group reached level 4 severity (hospitalization with need for supplemental oxygen), but this difference also was not statistically significant (Table 2). There were no deaths within the mITT population during the trial.

### Study Medication Adherence

In the fluvoxamine group, 60.3% of participants took at least 80% of the expected medication doses before either clinical deterioration or completion of planned 15-day treatment, compared with 74.5% in the placebo group (Supplementary Results 1). Among participants in the per-protocol analysis, clinical deterioration occurred in 8 (4.9%) of 164 in the fluvoxamine group and 11 (5.4%) of 205 in the placebo group (absolute difference at day 15, 0.46%; 95% CI, −4.0% to 5.0%; from survival analysis; log-rank *P* = .92) (Figure 2*B*).

### Adverse Events

Adverse events are summarized in Table 3. The most common events apart from COVID-19 respiratory decompensation were mild to moderate gastrointestinal symptoms, primarily nausea (8.1% of fluvoxamine vs 4.0% placebo) and insomnia (3.3% vs 2.9%). Eleven participants (4.0%) in the fluvoxamine group and 12 (4.4%) in the placebo group experienced serious adverse events (SAEs), the majority of which were for COVID-19-related hospitalization; none of the SAEs were judged due to study drug.

**Table 3.** Adverse Events

Adverse Events	No. of Participants With Event (%)		
	Fluvoxamine (n = 272)	Placebo (n = 275)	*P* Value^a^
Event details (by No. of individuals)^b^			
Nausea, vomiting, upset stomach, or gastroesophageal reflux	22 (8.1)	11 (4.0)	.05
Worsened dyspnea or respiratory failure	13 (4.8)	16 (5.8)	.70
Hypoxia or need for supplemental oxygen	12 (4.4)	11 (4.0)	.83
Insomnia or sleep problem	9 (3.3)	8 (2.9)	.81
Pneumonia or fluid/inflammation in lungs	8 (2.9)	7 (2.5)	.80
Dry mouth	8 (2.9)	10 (3.6)	.81
Worsened somnolence, fatigue, or low energy	7 (2.6)	5 (1.8)	.58
Vertigo or dizziness	6 (2.2)	3 (1.1)	.34
Memory problem, disorientation, brain fog, groggy, fuzzy, or confused	5 (1.8)	2 (0.7)	.28
Anxiety, jitteriness, or panic	5 (1.8)	2 (0.7)	.28
Diarrhea or loose stool	5 (1.8)	0	.03
Migraine or headache	5 (1.8)	4 (1.5)	.75
Weakness, loss of strength	5 (1.8)	3 (1.1)	.50
Worsened fever or chills	4 (1.5)	2 (0.7)	.45
Presyncope, lightheadedness, syncope, collapse, or loss of consciousness	4 (1.5)	1 (0.4)	.21
Poor appetite	4 (1.5)	0	.06
Increased cough or chest congestion	3 (1.1)	6 (2.2)	.50
Urinary tract infection	3 (1.1)	2 (0.7)	.68
Dehydration	2 (0.7)	2 (0.7)	1.00
Rash, hives, itching, or other allergy-like skin reaction	3 (1.1)	1 (0.4)	.37
Palpitations, tachycardia, or arrhythmia	3 (1.1)	1 (0.4)	.37
Sweating	3 (1.1)	0	.12
Chest pain, tightness, or pressure	2 (0.7)	5 (1.8)	.45
Pulmonary embolism	2 (0.7)	2 (0.7)	1.00
Muscle twitching, shaking, tremor, or other involuntary movement	2 (0.7)	2 (0.7)	1.00
Musculoskeletal pain	2 (0.7)	7 (2.5)	.18
Paresthesia/abnormal skin sensation	1 (0.4)	2 (0.7)	1.00
Gastrointestinal or abdominal pain	1 (0.4)	1 (0.4)	1.00
Constipation or bowel blockage	1 (0.4)	1 (0.4)	1.00
Negative thoughts or mood disturbance	1 (0.4)	1 (0.4)	1.00
Restlessness or akathisia	1 (0.4)	0	.50
Hair loss	1 (0.4)	0	.50
Skin infection	1 (0.4)	0	.50
Sinusitis	1 (0.4)	0	.50
Low blood pressure	1 (0.4)	0	.50
High blood pressure	1 (0.4)	0	.50
Hyperglycemia or diabetes	1 (0.4)	0	.50
Blood infection	1 (0.4)	0	.50
Low hemoglobin/anemia	1 (0.4)	0	.50
Pulmonary fibrosis	1 (0.4)	0	.50
Noninvasive ventilation (BiPAP)	1 (0.4)	0	.50
Reduced sense of smell or taste	0	1 (0.4)	1.00
Hematochezia (blood in stool)	0	1 (0.4)	1.00
Slurred speech (possibly due to Bell's palsy)	0	1 (0.4)	1.00
Hypophosphatemia	0	1 (0.4)	1.00
Elevated D-dimer	0	1 (0.4)	1.00
Vision problem	0	1 (0.4)	1.00
Appendicitis	0	1 (0.4)	1.00
Group totals^b^			
Total No. of event details extracted	162 (N/A)	125 (N/A)	N/A
Total No. of documented adverse events	82 (N/A)	77 (N/A)	N/A
Seriousness of event (by No. of individuals)^c^			
Serious adverse event	11 (4.0)	12 (4.4)	1.00
Other adverse events	43 (15.8)	36 (13.5)	.40
Any adverse events	53 (19.5)	46 (16.7)	.44

a Computed from a Fisher exact test.

b One documented event sometimes included multiple event details (multiple symptoms, problems, interventions, as described here), and some individuals had >1 documented adverse event, so event details were extracted from each documented event description to allow more meaningful reporting of specific types of symptoms/problems that occurred.

c Number of individuals who had at least 1 serious, other, or any adverse event is reported here. Some individuals had multiple serious and/or nonserious events. There were 13 total serious adverse events in the fluvoxamine group because 1 individual had 3 documented serious adverse events. Of those who had a serious adverse event, 1 person in the fluvoxamine group and 2 people in the placebo group also had a nonserious adverse event.

## DISCUSSION

This fully remote trial testing the efficacy of fluvoxamine for early treatment of COVID-19 was stopped early due to lower-than-expected rates of clinical deterioration. We were unable to demonstrate a benefit of fluvoxamine for preventing clinical deterioration. The study showed that fluvoxamine was safe in this setting.

This study was innovative in several ways. The contactless, fully remote, and decentralized design allowed for rapid recruitment in 2 countries, including 48 US states. The prespecified design focusing on the mITT group allowed for evaluation of only participants who began the intervention, despite both expected and unforeseen challenges in distributing study kits, including a 2-week period of substantial disruption to US shipping timelines due to extreme winter weather in February 2021. The mITT definition also allowed for the inevitabilities of interacting with remote participants, including that some people might never receive the package or never open it, might change their mind about participating, or require urgent medical attention before receiving the study materials. The definition we used for clinical progression incorporated not only hospitalizations, but also participants who met criteria for severe respiratory COVID-19, whether they were hospitalized or not.

When pooled with the 2 previously completed trials of fluvoxamine 100 mg twice or 3 times daily (STOP COVID 1 and TOGETHER), the relative risk of all-cause hospitalization for fluvoxamine vs placebo was 0.75 (95% CI, 0.58 to 0.97) [7]. When considering differences between the 3 fluvoxamine studies that examined this dose, it is important to consider timing in the pandemic and geography. For example, STOP COVID 1 began recruitment in April 2020, near the beginning of the pandemic, when SARS-CoV-2 variants were more likely to result in clinical deterioration, especially for outpatients early in the course of illness. The different start times and study locations also meant that different variants predominated, and as the pandemic continued, additional interventions and supportive care became more common, all potentially affecting outcomes. The trials used different primary outcomes, but it is possible to compare the overall rates of hospitalization for the combined fluvoxamine and placebo groups. The 2 North American studies had similar pooled hospitalization rates (3.9% for STOP COVID 1 [5], 4.2% for STOP COVID 2), while TOGETHER's was higher at 12% [8]. Another factor that may have contributed to the lack of an observed treatment effect in STOP COVID 2 is that fluvoxamine was not initiated early enough in the disease course to achieve a meaningful clinical benefit. Compared with both STOP COVID 1 and TOGETHER, participants in the STOP COVID 2 trial started treatment on average 1–2 days later relative to their date of first symptoms, largely due to the time required to ship study medication to participants located across a wide geographic area of the continental United States and Canada, and further complicated by untimely weather-related delays.

It is possible that concomitant medications influenced outcomes, perhaps affecting group differences in clinical deterioration and/or reducing the overall number of deteriorations that occurred. Metformin, a drug that has shown promise as a COVID-19 treatment [9, 10], was taken more frequently by patients in the placebo group (9%) vs the fluvoxamine group (3%). Individuals in the placebo group more often took systemic steroids, and a slightly larger number of individuals in the fluvoxamine group took inhaled steroids. While none of these group differences in steroid or metformin use were statistically significant, there is still a possibility that these drugs might have affected outcomes and thus influenced our results. Metformin has been hypothesized to have both antiviral and anti-inflammatory actions [10], so it might be best started early, but could have potential for benefit regardless of treatment timing. The influence of steroids may depend more strongly upon timing, as they could be detrimental if they interfere with a beneficial immune response to the virus early in the course of illness, but they may improve outcomes by reducing risk for further deterioration if given once the inflammatory phase has begun.

The ideal timing for fluvoxamine is not entirely clear, but based on hypothesized mechanisms, very early treatment may be best. It was primarily fluvoxamine's anti-inflammatory effect through agonist action at the S1R that prompted the initial STOP COVID 1 trial, but fluvoxamine also has several other potential mechanisms that may be of benefit in COVID-19. When used to treat depression or anxiety, SSRIs may take several weeks for full benefit, but fluvoxamine's S1R agonist action has immediate effects on the activity of a transcription factor that regulates the production of cytokines in response to inflammatory triggers [3]. If given early enough, fluvoxamine may be able to prevent an excessive inflammatory response to the virus, or if given later it may help to calm inflammation once it has begun. Of course, this may be less effective if the inflammatory response has already caused organ damage. It has been demonstrated that platelets are hyperactivated in COVID-19 patients, and this can contribute to both inflammation and risk of blood clots [11]. Fluvoxamine and other SSRIs can inhibit platelet activity, in part by preventing platelets from taking up and storing serotonin [12]. This may prevent hyperactivated platelets from releasing excessive amounts of serotonin that could have detrimental effects on multiple organs and body functions. Considering this antiplatelet mechanism, early treatment would also be optimal, so that platelets would be depleted of serotonin before they become hyperactivated. If used early, fluvoxamine may have potential to prevent the need of stronger anti-inflammatory and antiplatelet agents. Fluvoxamine's hypothesized antiviral effects through the FIASMA mechanism may also be most effective early, as this host-directed antiviral mechanism has been demonstrated to inhibit entry of the virus into cells [4].

It is unclear whether combining fluvoxamine with these other medications can produce additional benefit or what the ideal treatment timing for such combinations would be, though one could hypothesize that antiviral agents would be most effective if given early in the course of illness (or even before infection), strongly immune-suppressing drugs would be most beneficial once the inflammatory process has begun, and drugs that modulate inflammation without strongly suppressing immune response to the virus might be best started early enough to prevent the inflammatory phase and potentially reduce the need for steroids, then continued for about 2 weeks, until after the peak of the inflammatory phase. Combining drugs with different actions and/or using drugs hypothesized to have multiple potentially beneficial mechanisms, like fluvoxamine along with metformin or inhaled budesonide [9, 13], may be particularly useful.

Dosing may also be important, as 2 randomized trials testing lower-dose fluvoxamine at 50 mg twice daily found that it was not beneficial for shortening time to recovery or reducing severe disease [9, 14].

### Limitations

The fully remote design limited the ability to verify COVID-19 test results and vital signs beyond self-report in many cases, which could have biased the results toward the null; yet, this is similar to other trials of acute COVID-19 in which vital signs and first positive SARS-CoV-2 test were self-reported by participants, even when a valid test result was verified. Importantly, home oximeter ratings are imperfect, which could affect classification according to our own 2-part definition of clinical deterioration (especially for those with self-reported low oxygen saturation who were never hospitalized) and our modified WHO scale classification of 1 vs 2 (which is also influenced by outpatients’ self-reported oxygen saturation). Also, in most ITT trials, individuals for whom it is unclear whether they ever took study medication would be considered lost to follow-up rather than excluded from analysis. There is also a possibility that medications received outside of the trial, including metformin and inhaled or systemic corticosteroids, could have worsened or improved outcomes in 1 or both treatment groups, also biasing results toward the null. The enrolled population was also younger and healthier than anticipated, as evidenced by the low rate of clinical deterioration from COVID-19 in both groups. It is unclear why there were such low rates of clinical deterioration despite enrichment for high-risk individuals, but some possibilities include differences in illness severity depending on the circulating SARS-CoV-2 variants or benefits from concomitant treatments, which may have been more commonly used later in the pandemic.

Although this trial did not detect differences in clinical deterioration, clinical benefits of fluvoxamine observed in study settings with a higher overall rate of deterioration might not be applicable to lower-risk populations. Fluvoxamine appeared safe and was generally well tolerated.

## CONCLUSIONS

While we were not able to demonstrate an effect of fluvoxamine in this population of patients with early symptomatic COVID-19, this study highlights the feasibility and pragmatic nature of conducting a fully remote trial for acute treatment of COVID-19 or other emerging infections in outpatients. Further studies are warranted to examine the efficacy of fluvoxamine for COVID-19, alone and in combination with other therapies [13], particularly in regions with higher rates of clinical deterioration and hospitalization due to COVID-19, lower rates of vaccination, or where access to highly effective COVID-19 treatments remains low.

## Supplementary Material

ofad419_Supplementary_Data
