# Supplementary material for: The STOP COVID 2 Study: Fluvoxamine vs Placebo for Outpatients With Symptomatic COVID-19, a Fully Remote Randomized Controlled Trial
Source: Open Forum Infect Dis. 2023 Aug 8;10(8):ofad419. doi: 10.1093/ofid/ofad419 (PMC10445518; doi:10.1093/ofid/ofad419)
Supplement: ofad419_Supplementary_Data [file ofad419_supplementary_data.zip › Suplement 2_Online Content.pdf]

Supplemental 2 Online Content

Reiersen, AM, Mattar, C, Bender Ignacio, RA, et al. A fully-remote randomized controlled trial of fluvoxamine vs placebo for outpatients with symptomatic COVID-19. *JAMA*

eMethods 1. Details of inclusion and exclusion criteria

eMethods 2. Summary of primary outcome measurement and analysis

eResults 1. Early stop for futility

eResults 1. Medication adherence

eTable 1. Concomittant Medications

This supplemental material has been provided by the authors to give readers additional information about their work.

eMethods 1. Details of inclusion and exclusion criteria

<table>
<tr><th>Inclusion Criteria</th><th>Exclusion Criteria</th></tr>
<tr><td>

Men and women aged 30 and older[a]
Not currently hospitalized
Proven SARS-CoV-2 positive (per lab or physician report). Positive test is within 3 days prior to or during the current symptomatic episode[b]
Currently symptomatic with one or more of the following symptoms: fever, cough, myalgia, mild dyspnea, chest pain, diarrhea, nausea, vomiting, anosmia (inability to smell), ageusia (inability to taste), sore throat, nasal congestion.
Expectation, on screening, that the participant will start study medication within 7 days of symptom onset[c]
Able to provide informed consent.
Upon initial screening, participant reports one of the following risk factors for clinical deterioration: age≥40, racial/ethnic group African-American, Hispanic, South Asian, or Native American (including more than one race), or 1+ of the following medical conditions which increase risk for developing moderate-severe COVID illness:

obesity
hypertension,
diabetes
heart disease (coronary artery disease
history of myocardial infarction, or heart failure)
lung disease (eg asthma, COPD), immune disorder (eg rheumatoid arthritis, lupus).

</td><td>

Illness severe enough to require hospitalization or already meeting study’s primary endpoint for clinical worsening (eg current O_2 saturation <92% on room air, current use of supplemental oxygen to maintain O_2 saturation ≥92%).[c]
Unstable medical comorbidities (eg decompensated cirrhosis), per patient report and/or medical records.
Immunocompromised from the following: solid organ transplant, BMT, high dose steroids (>20mg prednisone per day), or tocilizumab
Already enrolled in another COVID 19 medication trial (not including vaccination or prophylaxis trials)
Unable to provide informed consent.
Unable to perform the study procedures.
Taking donepezil or sertraline.
Taking warfarin-also known as Coumadin, phenytoin, clopidogrel, and St John's wort.
Taking SSRIs, SNRIs, or tricyclic antidepressants, unless these are at a low dose such that a study investigator concludes that a clinically significant interaction with fluvoxamine (ie either serotonin syndrome or TCA overdose) is unlikely.
Individuals who report they have bipolar disorder or are taking medication for bipolar disorder (lithium, valproate, high-dose antipsychotic), unless the investigator concludes that the risk for mania is unlikely (ie it is doubtful that the patient actually has bipolar disorder).
Individuals who take alprazolam or diazepam and are unwilling to cut the medication by 25%.
Received vaccine for COVID-19. Note that participants in vaccine trials are eligible, unless they know that they received the active vaccine.
Individuals who are already taking an approved or investigational COVID-19 treatment, or have already received monoclonal antibody treatment or convalescent plasma. .

</td></tr>
</table>

Abbreviations: SARS-CoV-2, severe acute respiratory syndrome coronavirus 2; COVID-19, coronavirus disease 2019; COPD, chronic obstructive pulmonary disease; O_2, oxygen; mg, milligram; BMT, bone marrow transplant; S1R, sigma-1 receptor; SSRIs, selective serotonin reuptake inhibitors; SNRIs, serotonin and norepinephrine reuptake inhibitors; TCA, tricyclic antidepressant

[a] In February 2021, the minimum age inclusion was increased from 18 years old to 30 years old.

[b] Test positivity could be via participant self-report initially; however, staff also attempted to get confirmation.

[c] Participants who were randomized and then failed to start study medication by day 7 of symptoms or found to meet clinical deterioration at baseline or met deterioration criteria prior to starting study medication were allowed to remain in the study and could be managed and assessed just as other randomized participants. However, these participants were not included in the modified intent-to-treat analysis.

eMethods 2. Summary of Primary Outcome Measurement and Analysis

Below is a summary of the measurement and analysis for the primary outcome. See Supplement 1 for additional information regarding analysis.

Outcome	Definition	Measurement	Analysis
Clinical Deterioration[a]	(1) presence of dyspnea (Shortness of Breath Rating of 4+) and/or hospitalization for shortness of breath or pneumonia ***PLUS*** (2) decrease in oxygen saturation (<92% on room air) and/or supplemental oxygen requirement to keep oxygen saturation ≥92%).	(1) Twice-daily Severity Scale with range of 0 to 10 (2) Twice-daily at-home self-monitoring or via monitoring at a hospital/Emergency Room/urgent care center	Survival analysis using stratified log-rank statistic; stratified by site, sex at birth, and age group

[a] A pre-specified analysis for clinical deterioration based upon a modified version of the World Health Organization (WHO) Therapeutic Trial Synopsis 9-point scale was also conducted. This was done to directly compare findings to other clinical trials. The modified WHO scale measured clinical severity status at its worst during the trial. It was scored as follows: 0 = no clinical or virological evidence of infection; 1 = ambulatory, no activity limitation (For this trial, 1 = not meeting criteria for clinical deterioration and not hospitalized for COVID-related reason.); 2 = ambulatory, activity limitation (For this trial, clinically deteriorated but not hospitalized.); 3 = hospitalized, no supplemental oxygen; 4 = hospitalized, supplemental oxygen given; 5 = hospitalized, treated with non-invasive ventilation such as bilevel positive airway pressure (BiPAP), or high flow nasal cannula; 6 = hospitalized, intubated, treated with mechanical ventilation; 7 = hospitalized, intubated, mechanical ventilation, plus organ support needed; 8 = death. While blinded to treatment condition, one of the study Principal Investigators (AMR) examined data regarding all hospitalized and/or clinically deteriorated patients to assign the appropriate WHO scale score, and these ratings were also approved by the other principal investigator (EJL). A t-test was used to compare the scale between the fluvoxamine and placebo groups; the distribution of participants at each level of the scale was also compared.

eResults 1. Early stop for futility

A pre-planned interim analysis was conducted once the trial had recruited >50% of the originally planned number of participants for the main analysis. The a priori conditional power for futility had been set at 10%. The Data Safety Monitoring Board (DSMB) met on May 10, 2021. At the DSMB meeting, it was noted that recruitment had slowed, mainly due to increasing vaccination rates in high-risk adults. While the study was designed under the assumption of an overall 16.5% event rate (20% in the placebo arm and 13% in the fluvoxamine arm), the overall event rate was 26/527 (5%). Conditional power under the original design was presented at 22%. The team proposed in consultation with the DSMB updated assumptions of 8% event rate for the placebo group and 4% for the intervention group be used to estimate conditional power.

This <=5% event rate for progression to severe disease has subsequentially been observed in multiple outpatient COVID-19 clinical trials conducted in 2021 and 2022. Thus, the underlying assumptions of the trial were drastically altered in the vaccination era. The trial was halted in May 2021, before the onset of the SARS-CoV-2 Delta variant period.

eResults 1. Medication Adherence

In the fluvoxamine group, 60.3% of participants took ≥ 80% of expected doses of study medication until the time of deterioration or completion of planned 15-day treatment, in comparison to 74.5% in the placebo group.

A per-protocol analysis of TOGETHER found that patients who took ≥ 80% of study pills had a 66% reduction in risk for this primary outcome, suggesting the importance of adherence to the study medication. In STOP COVID 1, participants were instructed to increase fluvoxamine up to 100 mg three times daily, but only about half reached this target dose (300 mg). Most participants reached a dose of 100 mg twice daily. Because fluvoxamine showed evidence of benefit despite ~50% taking less than the target dose, we decided to have participants take a maximum of 100 mg twice daily for STOP COVID 2. Even with this lower target dose in STOP COVID 2, those taking fluvoxamine were more likely to stop their study medication, presumably due to medication side effects. It is important to achieve an effective dose quickly when treating an acute infection, but the required dose (and tolerability) may vary depending on individual differences in the speed of drug metabolism. Based on pharmacokinetic modeling, it has been suggested to ramp the fluvoxamine dose up to the target dose of 100 mg twice daily quickly to ensure the required drug level,[1] but consider de-escalating the dose if the drug is poorly tolerated.[2]

eTable 1. Concomittant Medications

The table below includes concomitant Medications taken by at least five percent of participants in one or both treatment groups (listed in order of frequency in the fluvoxamine group).

Drug or Drug Class	Fluvoxamine (N=272) n (%)	Placebo (N=275) n (%)
Vitamin D	62 (22.8%)	64 (23.3%)
Acetaminophen	60 (22.1%)	59 (21.5%)
NSAIDs	50 (18.4%)	49 (17.8%)
Multivitamins	47 (17.3%)	51 (18.5%)
Vitamin C	42 (15.4%)	40 (14.5%)
Bronchodilators	42 (15.4%)	30 (10.9%)
Antihistamines (H1 blockers)	41 (15.1%)	37 (13.5%)
Zinc	40 (14.7%)	32 (11.6%)
Any Antibiotic*	33 (12.1%)	24 (8.7%)
Non-azithromycin antibiotic	18 (6.5%)	9 (3.3%)
Azithromycin	16 (5.9%)	15 (5.5%)
HMG CoA Reductase Inhibitors (statins)	23 (8.5%)	30 (10.9%)
Proton Pump Inhibitors	23 (8.5%)	18 (6.5%)
Corticosteroids: oral or Intravenous	21 (7.7%)	28 (10.2%)
Thyroid Hormones	20 (7.4%)	31 (11.3%)
ACE Inhibitors	19 (7.0%)	20 (7.3%)
Corticosteroids: Inhaled or Intranasal	19 (7.0%)	15 (5.5%)
Thiazide or Thiazide-like Diuretics	16 (5.9%)	21 (7.6%)
Angiotensin II Receptor Blockers	16 (5.9%)	17 (6.2%)
Calcium	16 (5.9%)	7 (2.5%)
Beta Blockers	15 (5.5%)	19 (6.9%)
Dextromethorphan	15 (5.5%)	11 (4.0%)
Miscellaneous Herbal Supplements	14 (5.1%)	21 (7.6%)
Calcium Channel Blockers	13 (4.8%)	16 (5.8%)
Omega-3 Fatty Acids	12 (4.4%)	14 (5.1%)
Guaifenesin	9 (3.3%)	22 (8.0%)
Metformin	9 (3.3%)	17 (6.2%)

Additional medications and supplements, reported by <5% of participants: Remdesivir, monoclonal antibodies, vaccine, blood transfusion, convalescent plasma, tociluzimab, colchicine, allopurinol, hydroxychloroquine, quinine, oral ivermectin, topical ivermectin, methotrexate, integrin receptor antagonist monoclonal antibody (vedolizumab), tumor necrosis factor blocker (adalimumab), mesalamine, sulfasalazine, unspecified disease-modifying antirheumatic drugs, bismuth subsalicylate, muscle relaxers, baclofen, topical corticosteroids, benzonatate, metoclopramide, odansetron, H2-blockers, montelukast, decongestants, fenofibrate, aldosterone receptor agonists, hydralazine, non-thiazide diuretic, alpha-blocker (tamsulosin or doxazosin), beta-3 adrenergic agonists (mirabegron), alpha adrenergic agonist (clonidine, guanfacine), isosorbide, clodipogrel, apixaban, coumadin, heparin, Sodium-glucose cotransporter-2 inhibitor, glucagon-like peptide analogues, insulin, sulphonylurea, dipeptidyl peptidase-4 inhibitor, antithyroid (methimazole-tapazole), denosumab, bisphosphosphonate, buspirone, benzodiazepines, gabapentin, pregabalin, lamotrigine, topiramate, lithium, modafinil, stimulant, dopamine agonist, triptans, Calcitonin gene-related peptide blocker, selective serotonin reuptake inhibitor, serotonin-norepinephrine reuptake inhibitor, atomoxetine, bupropion, amitriptyline, trazodone, melatonin, sleep aids, antipsychotics, anticholinergics, cholinesterase inhibitor, anticholinergic, opioids, loperamide, butalbital, cannabinoids, luteone/lutein, Retin-A, vitamin A, topical azelaic acid, vitamin E, vitamin K, magnesium, potassium, iron, selenium, folate, biotin, vitamin B12, vitamin B2, vitamin B3, vitamin B6, n-acetyl-cysteine, taurine, glycerol monolaurate, glucosamine, probiotics, coenzyme Q10, lactoferrin, turmeric/curcumin, quercetin, amino acids, ophthalmic drops or ointments, valacyclovir, HIV pre-exposure prophylaxis, HIV antiretrovirals, testosterone, anabolic steroid (stanozolol), 5α-reductase inhibitor, luteinizing hormone-releasing hormone agonist, aromatase inhibitor, contraceptive, anti-estrogen, estrogen, selective estrogen receptor modulator, progestins, DHEA, antifungal, polyethylene glycol 3350.

*One individual took 2 different antibiotics (azithromycin plus amoxicillin), which is why the total number in the fluvoxamine group who took antibiotics is 33 rather than 34.

References

1. Dodds MG, Doyle EB, Reiersen AM, Brown F, Rayner CR. Fluvoxamine for the treatment of COVID-19. *Lancet Glob Health*. Mar 2022;10(3):e332. doi:10.1016/S2214-109X(22)00006-7
2. Marcec R, Likic R. Could Fluvoxamine Dose De-escalation Increase Treatment Compliance Without Sacrificing Efficacy in COVID-19 Patients? *Clin Pharmacokinet*. Sep 2022;61(9):1321-1323. doi:10.1007/s40262-022-01154-x
