# Supplementary material for: The STOP COVID 2 Study: Fluvoxamine vs Placebo for Outpatients With Symptomatic COVID-19, a Fully Remote Randomized Controlled Trial
Source: Open Forum Infect Dis. 2023 Aug 8;10(8):ofad419. doi: 10.1093/ofid/ofad419 (PMC10445518; doi:10.1093/ofid/ofad419)
Supplement: ofad419_Supplementary_Data [file ofad419_supplementary_data.zip › SC-2_SAP_original, final, summary of changes.pdf]

# Statistical Analysis Plan

## 1. Administrative information

### 1.1. Title and Trial Registration

Full study title: Fluvoxamine for early treatment of COVID-19: A fully-remote, randomized placebo controlled trial

Acronym: STOP COVID Trial 2

ClinicalTrials.gov identifier: NCT04668950

Washington University in St. Louis IRB ID number: 202011101

Funding: COVID-19 Early Treatment Fund ("CETF")

SAP version: 1.0

Study protocol version: 5.0 (April 14th, 2021)

SAP version history: -

SAP version justification: -

SAP Revision timing: Revision will be conducted after reviewing by PI and biostatistician. This version is the primary draft of the detailed SAPs for each outcome.

### 1.2. Roles and Responsibility

Author: Lei Yang, Statistical Data Analyst

Biostatistician: J. Philip Miller

Principal investigator: Eric Lenze, MD

Angela Reiersen, MD

SAP contributors and roles: Angela Stevens, Study Coordinator

Affiliations: 1. Washington University in St. Louis, Missouri, USA

2. Northwestern University, Illinois, USA

3. University of Utah, Utah, USA

4. Fred Hutchinson Center Clinical Research Center, Washington, USA

5. McGill University Health Center, Qc, Canada

6. University of Toronto, Ont., Canada

# Table of contents

1. Administrative information ....1
1.1. Title and Trial Registration ....1
1.2. Roles and Responsibility ....1
Table of contents ....2
Abbreviation ....4
2. Introduction ....5
2.1. Background and Rationale: ....5
2.2. Objectives ....6
2.2.1. Objectives and study aims ....6
2.2.2. Hypothesis ....6
2.2.3. Scope ....6
3. Study Methods ....6
3.1. General Study Design and Plan: ....6
3.2. Randomization ....6
3.3. Sample Size ....7
3.4. Framework ....7
3.5. Statistical interim analyses and stopping guidance ....7
3.6. Timing of final analysis ....7
3.7. Timing of outcome assessment ....8
4. Statistical principles ....8
4.1. Confidence intervals and p values ....8
4.2. Adherence and protocol deviations ....8
4.2.1. Definitions of protocol deviations ....8
4.2.2. Adherence and protocol deviations ....8
4.2.3. Electronic data capture ....8
4.3. Analysis populations ....8
5. Trial Population ....8
5.1. Screening data ....8
5.2. Eligibility ....8
5.2.1. Inclusion Criteria ....9
5.2.2. Exclusion Criteria ....9
5.3. Recruitment ....10

**5.4. Withdrawal/follow-up** ........ 10
**5.4.1. General withdrawal rules** ........ 10
**5.5. Baseline participant characteristics** ........ 10
**5.5.1. List of baseline characteristics** ........ 10
**5.5.2. Descriptive summarization of baseline participant characteristics** ........ 11
**6. Analysis** ........ 11
**6.1. Outcome definitions** ........ 11
**6.1.1. Primary and secondary outcomes** ........ 11
**6.1.2. Outcome measurements** ........ 11
**6.2. Analysis methods** ........ 12
**6.3. Missing data** ........ 12
**6.4. Harms** ........ 12
**6.5. Statistical software** ........ 13

# Abbreviation

| | |
|---|---|
| AE | Adverse Event |
| AIDS | Acquired Immunodeficiency Syndrome |
| BMI | Body Mass Index |
| BMT | Bone Marrow Transplantation |
| COVID-19 | 2019 Novel Coronavirus Disease |
| DSMB | Data Safety and Monitoring Board |
| e-CRF | Electronic Case Report Form |
| GCP | Good Clinical Practice |
| ICU | Intensive Care Unit |
| IRB | Institutional Review board |
| mITT | Modified Intention-to-treat |
| PI | Principal Investigator |
| PROMIS | Patient Reported Outcome Measurement Information System |
| RCT | Randomized Controlled Trial |
| REDCap | Research Electronic Data Capture |
| S1R | Sigma-1 Receptor |
| SAE | Serious Adverse Event |

# 2. Introduction

## 2.1. Background and Rationale:

Coronavirus disease 2019 (COVID-19), caused by the novel severe acute respiratory syndrome coronavirus 2 (SARS-CoV-2), can cause serious illness including lung damage, hypoxia, and cardiac damage, which can lead to hospitalization, intensive care unit (ICU) admission, and death.

Clinical deterioration often occurs during the second week of illness. Early investigations of COVID-19 found that serious illness leading to hospital admission occurred a median of 8-10 days after initially mild to moderate symptoms.2-4 Evidence that many patients with COVID-19 develop lung damage from an excessive inflammatory response led to recommendations to repurpose immunomodulatory drugs to counter this hyperinflammation.5,6 More recent evidence supports the assertion that the inflammatory reaction, including elevations in cytokines such as IL-6, predicts clinical deterioration.

Many patients have debilitating persistent symptoms and loss of function, even three or more months after the initial stage of illness. The causes of this persistent illness are unclear and likely heterogeneous; they include persistence of the viral infection, immunological changes, and exacerbation of underlying medical conditions. As a result, both anti-viral and immunomodulatory drugs could potentially prevent or reduce this post-COVID functional impairment.

Fluvoxamine is unique among the SSRIs in that it strongly activates the sigma-1 receptor (S1R), and preclinical studies have suggested that by this mechanism, fluvoxamine could prevent the immunopathology seen in serious COVID-19. In 2019, it was shown that fluvoxamine, given early in treatment, reduced deterioration and mortality in two animal models of sepsis (bacterial peritonitis and lipopolysaccharide stimulation). The same study demonstrated that fluvoxamine was anti-inflammatory in human cells, reducing IL-6 and other cytokines in lipopolysaccharide-induced inflammation. This study is part of an increasing body of research supporting the S1R in protection from deleterious consequences of inflammation.

Fluvoxamine has one of the strongest S1R agonist effects of any existing medication. It is also highly lipophilic and has rapid, substantial intracellular uptake.16 Fluvoxamine also has many pragmatic advantages for repurposing, including ease of use, high safety margin, good tolerability, wide availability, and low cost. We determined that if it could be shown that fluvoxamine had a beneficial effect in reducing clinical deterioration of COVID-19, the drug (and other S1R agonists) could be an important addition to the armamentarium vs. this pandemic.

A preliminary single-site STOP COVID Trial (ClinicalTrials.gov Identifier: NCT04342663) aimed to test whether a course of fluvoxamine given early in COVID-19 illness could prevent clinical deterioration was conducted in Missouri and Illinois from April 10th to September 19th, 2020. The key finding from the preliminary study, 0% (0/80) of fluvoxamine-treated individuals vs. 8.3% (6/72) of those who received placebo suffered clinical deterioration, demonstrated that fluvoxamine prevented, rather than merely delayed, deterioration.

Therefore, we conducted a multi-site, double-blind, placebo-controlled RCT to test whether fluvoxamine, given as early treatment in individuals with mild COVID-19 illness, might prevent clinical deterioration.

## 2.2. Objectives

### 2.2.1. Objectives and study aims

The study aims to replicate the preliminary findings from the previous single-site trial in a larger confirmatory multi-site trial. First, we will examine whether fluvoxamine prevents clinical deterioration in early COVID-19. We will use the presence of dyspnea and decrease in $O_2$ saturation to determine clinical deterioration. Second, we will find out whether fluvoxamine mitigates the long-term impact of COVID-19. We will compare participants randomized to fluvoxamine to those randomized to placebo by comparing their post-COVID functional status and symptoms at day 15 and day 90. Third, we will explore ① whether the benefits of fluvoxamine for preventing deterioration vary as a function of baseline $O_2$ saturation; ② whether participants' co-prescribed medications vary between the fluvoxamine and placebo groups.

### 2.2.2. Hypothesis

H1a: Participants randomized to fluvoxamine will have a lower rate of clinical deterioration compared to placebo.

H1b: Participants with lower baseline $O_2$ saturation will have a larger treatment effect size (i.e., larger fluvoxamine-placebo difference in the rate of clinical deterioration) than those with higher $O_2$ saturation. We will use a median split on baseline $O_2$ saturation for a test of this hypothesis.

H2: Participants randomized to fluvoxamine will have higher post-COVID functional status at day 15 and day 90, compared to participants randomized to placebo.**2.2.3. Scope**

This Statistical Analysis Plan will be the guiding document for the quantitative analyses that will be conducted in the STOP COVID Trial 2.

# 3. Study Methods

## 3.1. General Study Design and Plan:

A fully-remote study in the US and Canada will be adopted for our trial. Participants will be screened and consented over the phone. Study drug fluvoxamine/placebo and other supplies will be delivered to (self-quarantined) participants, including thermometers, blood pressure cuffs, and O2 saturation monitors. Data will be collected via participants' phones/computers and medical records. National recruitment in the US and regional recruitment in Canada will be carried out, similar to the University of Minnesota COVID-19 hydroxychloroquine trials conducted in early 2020.

Participants will be recruited nationally in the US and regions in Canada by cooperating with 6 universities/centers. The six universities/centers are Washington University in St. Louis; Northwestern University; University of Utah; Fred Hutchinson; McGill University Health Center in Canada; and University of Toronto in Canada.

## 3.2. Randomization

Participants in the US are randomized 1:1 to fluvoxamine or matching placebo capsules. A study statistician will generate randomization schedules stratified by age and sex. Treatments will be randomly allocated using alternating blocks of sizes 2 and 4. Randomization allocation is conducted via REDCap, which displays randomization assignments to research team members who prepare study materials including drug or placebo but otherwise have no contact with participants. All individuals in contact with participants are blinded, including outcome assessors and investigators, as are participants themselves.

The same randomization strategy is performed at the Canada sites.

## 3.3. Sample Size

Our original proposal was to recruit 1100 participants in order to have 880 participants in the modified intention-to-treat sample. This sample will include all enrolled participants who were confirmed eligible at the baseline assessment and reporting taking at least one dose of study medication. A modified intention-to-treat strategy is required because participants will be shipped supplies and study medication (whereupon they are randomized) prior to their measuring oxygen saturation to confirm study eligibility.[1]

The planned recruitment number was adjusted to approximately 1100 participants in the mITT group mid-study and had led to an early stop due to slow recruitment rate and low event rate in the current mITT sample. This was because most at-risk adults in the US and Canada are now vaccinated and not only have cases dropped considerably but also the cases are mostly occurring among those either at low risk (who would not be eligible) or who are vaccine-hesitant (who may be less likely be willing to participate in medical research). The last enrollment date was May 21st, 2021, with a total of 669 participants randomized and 551 participants included in the mITT sample.

## 3.4. Framework

Hypothesis testing framework: $H_0$ and $H_1$ with confidence intervals for differences.

## 3.5. Statistical interim analyses and stopping guidance

A pre-planned interim analysis was conducted once the trial had recruited >50% of the originally planned number of participants for the planned main analysis. The DSMB met on May 10th, 2021. There had been 657 participants randomized, with 527 of the 880 participants in the mITT sample(data presented as of May 5th, 2021). The study team noted that recruitment had recently slowed, citing that the availability of vaccinations was likely the reason. No safety signals were noted after reviewing adverse events and serious adverse events. While the study was initially designed under the assumption of an overall 16.5% event rate (20% in the placebo arm and 13% in the fluvoxamine arm), the overall event rate based on the data presented as of May 5th, 2021, was 5%. Conditional power under the original design was presented at 22%. The team proposed updated assumptions of 8% for the placebo group and 4% for the fluvoxamine group. With those updated assumptions, the necessary mITT sample size would be approximately 550 participants per arm, a more than 20% increase. The conditional power under the updated assumptions would be approximately 5%. The DSMB recommended an early stop, and a decision was made on May 19th, 2021 to stop the study due to futility. Participants already enrolled through May 19, 2021 completed the 15-day trial as well as the planned 90-day post-trial questionnaires to assess longer term outcomes.

## 3.6. Timing of final analysis

H1a and H1b analyses have ensued after we decided to early stop recruitment on May 21st, 2021 and after all the active enrolled participants reach day 15 in the RCT phase. H2 analysis will ensue after all enrolled participant completes day 90 in the follow-up phase.

This statistical analysis plan was added to the study protocol at clinicaltrials.gov, before the closure of the database and before any analyses had been conducted. Independent study monitoring was conducted in adherence to the Good Clinical Practice(GCP) guidelines. The statistician will be blind to treatment assignments during the H1a and H1b analysis and unblind during H2 and exploratory analysis.

[1] In the preliminary study (STOP COVID Trial), approximately 20% of individuals who received study supplies were not in the intention-to-treat sample, mainly because study eligibility could not be confirmed.

### 3.7. Timing of outcome assessment

Outcome assessments will be conducted at the end of the RCT phase on day 15. During the follow-up phase, assessments will be conducted on day 90.

# 4. Statistical principles

## 4.1. Confidence intervals and p values

To test our primary hypothesis (H1) and secondary hypothesis (H2), a significance level of .05 will be considered statistically significant. Results will be presented with their values (e.g., coefficients, standard deviation, etc.) with 95% confidence intervals.

## 4.2. Adherence and protocol deviations

### 4.2.1. Definitions of protocol deviations

Any alteration or modification to the IRB-approved research will not be conducted without prospective IRB approval. The term research encompasses all IRB-approved materials and documents including the detailed protocol, IRB application, consent form, recruitment materials, questionnaires/data collection forms, and any other information relating to the research study.

### 4.2.2. Adherence and protocol deviations

As a pragmatic study, all levels of adherence are allowed. Compliance with the study-recommended medication and dosing will be followed during the trial. Reasons for non-compliance and protocol deviations will be documented.

### 4.2.3. Electronic data capture

This study will utilize REDCap-based electronic Case Report Forms (e-CRF's) for data capture and storage. All study-related materials will be stored electronically in REDCap or a secured university server. No physical source documents are anticipated.

## 4.3. Analysis populations

We will follow the modified intention-to-treat (mITT) principle. In other words, participants who stop randomized medication will continue to be followed. To confirm that mITT findings represent the "true" data on effectiveness, safety, and tolerability of management strategies, we will conduct a sensitivity analysis that will compare mITT to per-protocol findings.

# 5. Trial Population

## 5.1. Screening data

Potential participants identified through the IRB-approved recruitment plan will be screened for eligibility. Staff and PI's at each site are responsible for verifying that participants meet all eligibility criteria before being randomized to acute treatment. All sites in the US and Canada follow the same screening requirements.

## 5.2. Eligibility

All participants will meet the following eligibility criteria:

### 5.2.1. Inclusion Criteria

a) Men and women aged 30 and older.[2]

b) Not currently hospitalized.

c) Proven SARS-CoV-2 positive (per lab or physician report). A positive test[3] is within 3 days prior to or during the current symptomatic episode.

d) Currently symptomatic with one or more of the following symptoms: fever, cough, myalgia, mild dyspnea, chest pain, diarrhea, nausea, vomiting, anosmia (inability to smell), ageusia (inability to taste), sore throat, nasal congestion.

e) Expectation, on screening, that the participant will start study medication within 7 days of symptom onset.

f) Able to provide informed consent.

g) Upon initial screening, participant reports one of the following risk factors for clinical deterioration: age $\geqslant$40, racial/ethnic group African-American, Hispanic, South Asian, or Native American (including more than one race), or 1+ of the following medical conditions which increase risk for developing moderate-severe COVID illness: obesity, hypertension, diabetes, heart disease (coronary artery disease, history of myocardial infarction, or heart failure), lung disease (e.g., asthma, COPD), immune disorder (e.g., rheumatoid arthritis, lupus).

### 5.2.2. Exclusion Criteria

a) Illness severe enough to require hospitalization or already meeting study's primary endpoint for clinical worsening[4] (e.g., current O2 saturation <92% on room air, current use of supplemental oxygen to maintain O2 saturation $\geqslant$92%).

b) Unstable medical comorbidities (e.g., decompensated cirrhosis), per patient report and/or medical records.

c) Immunocompromised from the following: solid organ transplant, BMT, high dose steroids (>20mg prednisone per day), or tocilizumab.[5]

d) Already enrolled in another COVID 19 medication trial (not including vaccination or prophylaxis trials)

e) Unable to provide informed consent.

f) Unable to perform the study procedures.

g) Taking donepezil, or sertraline.

h) Taking warfarin-also known as Coumadin, phenytoin, clopidogrel, and St John's wort.

---

[2] Inclusion criteria was: Men and Women aged 18 and older. Adjusted after first DSMB call on Feb 8th,2021

[3] Due to the study nature, it is not possible to acquire a positive test confirmation from every participant at the time of the recruitment.

[4] participants who are randomized and then found to meet clinical deterioration criteria at baseline – or meet deterioration criteria prior to starting study medication - will (or may) remain in the study and be managed and assessed just as other randomized participants. They will not be included in the modified intention to treat sample during data analysis.

[5] Older version: Immunocompromised (solid organ transplant, BMT, AIDS, on high dose steroids (>20mg prednisone per day).

i) Taking SSRIs, SNRIs, or tricyclic antidepressants, unless these are at a low dose such that a study investigator concludes that a clinically significant interaction with fluvoxamine (i.e., either serotonin syndrome or TCA overdose) is unlikely.

j) Individuals who report they have bipolar disorder or are taking medication for bipolar disorder (lithium, valproate, high-dose antipsychotic) unless the investigator concludes that the risk for mania is unlikely.

k) Individuals who take alprazolam or diazepam and are unwilling to cut the medication by 25%.

l) Participants taking theophylline, tizanidine, clozapine, or olanzapine will be reviewed with a study investigator and excluded unless the investigator concludes that the risk to the participant is low.

m) Received vaccine for COVID-19.

n) Individuals who are already taking an approved or investigational COVID-19 treatment or have already received monoclonal antibody treatment or convalescent plasma.

## 5.3. Recruitment

A consort diagram will be used to visualize the flow of participants. We will report the number of eligible participants and the number of screened participants. The reasons for lack of eligibility will be enumerated. The number randomized to the mITT group will be reported as well as the reasons for non-randomized to mITT group. The number randomized into either arm (fluvoxamine vs. placebo) will be reported.

## 5.4. Withdrawal/follow-up

### 5.4.1. General withdrawal rules

Participants are free to withdraw from participation in the study at any time upon request.
The PI may discontinue or withdraw a participant from the study for the following reasons:

- The participant is not contributing data and/or appears to be lost to follow-up.
- If any clinical adverse event (AE), laboratory abnormality, or other medical condition or situation occurs such that continued participation in the study would not be in the best interest of the participant or interferes with the integrity of the study data.
- Disease progression which requires discontinuation of the study intervention
- If the participant meets an exclusion criterion (either newly developed or not previously recognized) that precludes further study participation

After completing day 15 in the RCT phase, participants will enter the follow-up phase till day 90.

## 5.5. Baseline participant characteristics

### 5.5.1. List of baseline characteristics

Participants will sign informed consent forms at baseline. Local research team assessments elements collected during baseline:

- Temperature
- Oxygen level
- Blood pressure
- Pulse
- Self-assessed symptoms
  - Fever
  - Cough

        - Shortness of breath
        - Fatigue/Weakness
        - Chills
        - Sore throat
        - Nasal congestion
        - Nausea
        - Body aches
        - Diarrhea
        - Loss of appetite
        - Loss of smell
        - Loss of taste
    - Medical history

The key variables being used in the study include:

- Age
- Gender
- Race
- Sites
- Days from symptom onset to start trial medication
- BMI
- Coexisting conditions(medical history)
- Self-assessed symptoms
- Oxygen level
- Randomization
- Endpoint for clinical worsening
- Days in RCT phase free of clinical deterioration

### 5.5.2. Descriptive summarization of baseline participant characteristics

We will list general patient characteristics in a baseline characteristics table. Continuous data will be presented as the mean with standard deviation (SD), median with interquartile range, and range(min-max). Dichotomous and categorical data will be presented in proportions.

# 6. Analysis

## 6.1. Outcome definitions

### 6.1.1. Primary and secondary outcomes

Primary: Participants who have a ① presence of dyspnea and/or hospitalization for shortness of breath or pneumonia, and ② $O_2$ saturation decrease (<92% on room air via the twice-daily at-home self-monitoring or via monitoring at a hospital/ER/urgent care center, and/or supplemental oxygen requirement to keep O2 saturation ⩾92%) will be clinical deteriorated.

Secondary: Secondary outcome will compare post-COVID functioning at day 15 and day 90 using the PROMIS 10-item Global Health Scale. It reflects the increasing concern regarding the persistence of COVID-19 illness and functional impairment in a substantial percentage of individuals with initially "mild" symptoms. We will also explore persistent symptoms of COVID-19 at these time points.

### 6.1.2. Outcome measurements

Primary outcome (clinical deterioration) will be measured by ① an SOB rating of 4+ on the twice-daily 0-10 scale and/or hospitalization for shortness of breath or pneumonia during study day 1- day 15 and ②

$O_2$ readings <92% on room air via the twice-daily at-home self-monitoring or monitoring at a hospital/ER/urgent care center, and/or supplemental oxygen requirement to keep $O_2$ saturation ⩾92% during study day 1- day 15.

Secondary outcome will be measured by a PROMIS 10-item Global Health Scale on study day 15 and day 90.

Two explanatory analyses will be performed. First, we will examine the outcomes of randomized participants but discovered to meet the study endpoint of clinical deterioration at baseline. We will examine whether those randomized to fluvoxamine have lower hospitalization rates, ventilator care, and mortality, and we will gather the same outcomes at day 15 and day 90 post-randomization. Second, we will explore co-prescribed medications to look for patterns of difference between the fluvoxamine and placebo groups.

## 6.2. Analysis methods

To test hypothesis H1a (clinical deterioration), survival analysis with the log-rank statistic stratified by US vs Canada, gender, and age group will be performed. Participants who meet the primary outcome or fill out the outcome assessment on the last day will be treated as the censored sample.

To test hypothesis H1b (treatment effect size), a Cox proportional hazard model will fit the data using strata as in H1a with factors as US vs. Canada, gender, and age group, $O_2$ level, and their interaction. The test for moderation would be a test of the interaction between treatment and baseline $O_2$ saturation (dichotomized with a median split). Given the decision for an early stop, this moderator analysis will not be done.

To test hypothesis H2 (post-COVID functioning), an ANOVA test stratified by US vs. Canada, gender, and age will be performed for day 15 and day 90. Pairwise comparison between treatment groups will be tested.

In terms of the exploratory outcomes, descriptive statistics will be performed. If the prevalence of persistent symptoms is adequate, we will explore post-hoc regression models which predict persistent symptoms based on the duration and severity of the particular symptom during the acute phase and other clinical features during the acute phase.

## 6.3. Missing data

Missing data can occur from patient dropout/withdrawal (unit nonresponse), meeting study endpoint, failure to provide data (item nonresponse), or administrative issues. We will minimize missing data by closely monitoring accumulating data for missingness and implement data quality control(QC) checks to reduce it when identified. Any missing data or anomalies will be communicated to the site(s) for clarification/resolution. When a patient drops out of the study, we will document the reason for dropout and who made the dropout decision. No imputation for missing data will be used for H1a and H1b analysis. We will use a multiple imputation analysis to estimate potential bias from missing data.

## 6.4. Harms

During the study, serious adverse events (SAE) and adverse events (AE) will be monitored continuously throughout the study and will be reported to the DSMB and IRB according to pre-specified requirements. SAE/AE rates and interim study results will be reviewed and discussed by the DSMB at the DSMB meetings.

## 6.5. Statistical software

Data cleaning and statistical analyses will be performed using SAS (SAS Institute, Cary NC) and R (The R Foundation for Statistical Computing; Vienna, Austria).

# Statistical Analysis Plan

## 1. Administrative information

### 1.1. Title and Trial Registration

Full study title: Fluvoxamine for early treatment of COVID-19: A fully-remote, randomized placebo controlled trial

Acronym: STOP COVID Trial 2

ClinicalTrials.gov identifier: NCT04668950

Washington University in St. Louis IRB ID number: 202011101

Funding: COVID-19 Early Treatment Fund ("CETF")

SAP version: 5.0 (11/15/2021)

Study protocol version: 5.0 (4/14/2021)

SAP version history: 1.0 (7/13/2021), 2.0 (9/16/2021), 3.0(9/29/2021), 4.0(10/20/2021)

SAP version justification: Each version is modified and edited after being reviewed by PIs and biostatistician.

SAP Revision timing: Revision will be conducted after reviewing by PIs and biostatistician. This version is the final version of the SAP.

### 1.2. Roles and Responsibility

Author: Lei Yang, Statistical Data Analyst

Biostatistician: J. Philip Miller

Principal investigator: Eric Lenze, MD

Angela Reiersen, MD

SAP contributors and roles: Angela Stevens, Study Coordinator

Affiliations: 1. Washington University in St. Louis, Missouri, USA

2. Northwestern University, Illinois, USA

3. University of Utah, Utah, USA

4. Fred Hutchinson Center Clinical Research Center, Washington, USA

5. McGill University Health Center, Qc, Canada

6. University of Toronto, Ont., Canada

# Table of contents

**1. Administrative information** ........ 1
**1.1. Title and Trial Registration** ........ 1
**1.2. Roles and Responsibility** ........ 1
**Table of contents** ........ 2
**Abbreviation** ........ 4
**2. Introduction** ........ 5
**2.1. Background and Rationale:** ........ 5
**2.2. Objectives** ........ 6
**2.2.1. Objectives and study aims** ........ 6
**2.2.2. Hypothesis** ........ 6
**2.2.3. Scope** ........ 6
**3. Study Methods** ........ 7
**3.1. General Study Design and Plan:** ........ 7
**3.2. Randomization** ........ 7
**3.3. Sample Size** ........ 7
**3.4. Framework** ........ 7
**3.5. Statistical interim analyses and stopping guidance** ........ 7
**3.6. Timing of final analysis** ........ 8
**3.7. Timing of outcome assessment** ........ 8
**4. Statistical principles** ........ 8
**4.1. Confidence intervals and p values** ........ 8
**4.2. Adherence and protocol deviations** ........ 8
**4.2.1. Definitions of protocol deviations** ........ 8
**4.2.2. Adherence and protocol deviations** ........ 8
**4.2.3. Electronic data capture** ........ 8
**4.3. Analysis populations** ........ 9
**5. Trial Population** ........ 9
**5.1. Screening data** ........ 9
**5.2. Eligibility** ........ 9
**5.2.1. Inclusion Criteria** ........ 9
**5.2.2. Exclusion Criteria** ........ 10
**5.3. Recruitment** ........ 10

**5.4. Withdrawal/follow-up** ........ 11
**5.4.1. General withdrawal rules** ........ 11
**5.5. Baseline participant characteristics** ........ 11
**5.5.1. List of baseline characteristics** ........ 11
**5.5.2. Descriptive summarization of baseline participant characteristics** ........ 12
**6. Analysis** ........ 12
**6.1. Outcome definitions** ........ 12
**6.1.1. Primary and secondary outcomes** ........ 12
**6.1.2. Outcome measurements** ........ 12
**6.2. Analysis methods** ........ 13
**6.3. Missing data** ........ 13
**6.4. Harms** ........ 13
**6.5. Statistical software** ........ 13

# Abbreviation

| | |
|---|---|
| AE | Adverse Event |
| AIDS | Acquired Immunodeficiency Syndrome |
| ANOVA | Analysis of Variance |
| BMI | Body Mass Index |
| BMT | Bone Marrow Transplantation |
| COPD | Chronic Obstructive Pulmonary Disease |
| COVID-19 | 2019 Novel Coronavirus Disease |
| DSMB | Data Safety and Monitoring Board |
| e-CRF | Electronic Case Report Form |
| GCP | Good Clinical Practice |
| ICU | Intensive Care Unit |
| IRB | Institutional Review board |
| mITT | Modified Intention-to-treat |
| PI | Principal Investigator |
| PROMIS | Patient Reported Outcome Measurement Information System |
| QC | Quality Control |
| RCT | Randomized Controlled Trial |
| REDCap | Research Electronic Data Capture |
| S1R | Sigma-1 Receptor |
| SAE | Serious Adverse Event |
| SD | Standard Deviation |
| SNRIs | Serotonin and Norepinephrine Reuptake Inhibitors |
| SOB | Shortness of Breath |
| SSRIs | Selective Serotonin Reuptake Inhibitors |
| TCA | Tricyclic Antidepressant |

# 2. Introduction

## 2.1. Background and Rationale:

Coronavirus disease 2019 (COVID-19), caused by the novel severe acute respiratory syndrome coronavirus 2 (SARS-CoV-2), can cause serious illness including lung damage, hypoxia, and cardiac damage, which can lead to hospitalization, intensive care unit (ICU) admission, and death.

Clinical deterioration often occurs during the second week of illness. Early investigations of COVID-19 found that serious illness leading to hospital admission occurred a median of 8-10 days after initially mild to moderate symptoms. Evidence that many patients with COVID-19 develop lung damage from an excessive inflammatory response led to recommendations to repurpose immunomodulatory drugs to counter this hyperinflammation. More recent evidence supports the assertion that the inflammatory reaction, including elevations in cytokines such as IL-6, predicts clinical deterioration.

Many patients have debilitating persistent symptoms and loss of function, even three or more months after the initial stage of illness. The causes of this persistent illness are unclear and likely heterogeneous; they include persistence of the viral infection, immunological changes, and exacerbation of underlying medical conditions. As a result, both anti-viral and immunomodulatory drugs could potentially prevent or reduce this post-COVID functional impairment.

Fluvoxamine is unique among the SSRIs in that it strongly activates the sigma-1 receptor (S1R), and preclinical studies have suggested that by this mechanism, fluvoxamine could prevent the immunopathology seen in serious COVID-19. In 2019, it was shown that fluvoxamine, given early in treatment, reduced deterioration and mortality in two animal models of sepsis (bacterial peritonitis and lipopolysaccharide stimulation). The same study demonstrated that fluvoxamine was anti-inflammatory in human cells, reducing IL-6 and other cytokines in lipopolysaccharide-induced inflammation. This study is part of an increasing body of research supporting the S1R in protection from deleterious consequences of inflammation.

Fluvoxamine has one of the strongest S1R agonist effects of any existing medication. It is also highly lipophilic and has rapid, substantial intracellular uptake.16 Fluvoxamine also has many pragmatic advantages for repurposing, including ease of use, high safety margin, good tolerability, wide availability, and low cost. Therefore, we determined that if it could be shown that fluvoxamine had a beneficial effect in reducing clinical deterioration of COVID-19, the drug (and other S1R agonists) could be an important addition to the armamentarium vs. this pandemic.

A preliminary single-site STOP COVID Trial (ClinicalTrials.gov Identifier: NCT04342663) aimed to test whether a course of fluvoxamine given early in COVID-19 illness could prevent clinical deterioration was conducted in Missouri and Illinois from April 10th to September 19th, 2020. The key finding from the preliminary study, 0% (0/80) of fluvoxamine-treated individuals vs. 8.3% (6/72) of those who received placebo suffered clinical deterioration, demonstrated that fluvoxamine prevented, rather than merely delayed, deterioration.

Therefore, we conducted a multi-site, double-blind, placebo-controlled RCT to test whether fluvoxamine, given as early treatment in individuals with mild COVID-19 illness, might prevent clinical deterioration.

## 2.2. Objectives

### 2.2.1. Objectives and study aims

The study aims to replicate the preliminary findings from the previous single-site trial in a larger confirmatory multi-site trial. First, we will examine whether fluvoxamine prevents clinical deterioration in early COVID-19. Next, we will use the presence of dyspnea and decrease in $O_2$ saturation to determine clinical deterioration. We will also use a modified version of the World Health Organization (WHO) COVID-19 Therapeutic Trial Synopsis 9-point scale as a measure of clinical severity at its worst. The modified scale is defined as:

- 0 = no clinical or virological evidence of infection (none in our study should have this rating);
- 1= no limitation of activities (re-defined for this study as no hospitalization and no clinical deterioration by our study definition),
- 2= limitation of activities (re-defined for this study as no hospitalization but did have clinical deterioration by our study definition),
- 3= hospitalized but did not require supplemental oxygen;
- 4= hospitalized and required supplemental oxygen;
- 5= hospitalized and required non-invasive ventilation;
- 6= required intubation & ventilation;
- 7= Ventilator plus organ support needed;
- 8=death

Second, we will find out whether fluvoxamine mitigates the long-term impact of COVID-19 by comparing their post-COVID functional status and symptoms at day 15 and day 90 between the fluvoxamine group and placebo group. Third, we will explore ① study medication adherence between the fluvoxamine and placebo group; ② whether participants' co-prescribed medications vary between the fluvoxamine and placebo groups; ③ difference of persistent clinical symptoms at day 15 and day 90 between fluvoxamine and placebo group; and ④ difference of symptom onset days (from symptom onset to start of medication) between fluvoxamine and placebo group, and between deteriorated and non-deteriorated group.

### 2.2.2. Hypothesis

H1a: Participants randomized to fluvoxamine will have a lower rate of clinical deterioration and a higher level of clinical severity as compared to placebo.

H1b: Participants with lower baseline $O_2$ saturation will have a larger treatment effect size (i.e., a larger fluvoxamine-placebo difference in the rate of clinical deterioration) than those with higher $O_2$ saturation. We will use a median split on baseline $O_2$ saturation for a test of this hypothesis.

H2: Participants randomized to fluvoxamine will have higher post-COVID functional status at day 15 and day 90, compared to participants randomized to placebo.

### 2.2.3. Scope

This Statistical Analysis Plan will be the guiding document for the quantitative analyses conducted in the STOP COVID Trial 2.

# 3. Study Methods

## 3.1. General Study Design and Plan:

A fully-remote study in the US and Canada will be adopted for our trial. Participants will be screened and consented over the phone. Study drug fluvoxamine/placebo and other supplies will be delivered to (self-quarantined) participants, including thermometers, blood pressure cuffs, and $O_2$ saturation monitors. Data will be collected via participants' phones/computers and medical records. National recruitment in the US and regional recruitment in Canada will be carried out, similar to the University of Minnesota COVID-19 hydroxychloroquine trials conducted in early 2020.

Participants will be recruited nationally in the US and regions in Canada by cooperating with 6 universities/centers. The six universities/centers are Washington University in St. Louis; Northwestern University; University of Utah; Fred Hutchinson Cancer Research Center; McGill University Health Center in Canada; and the University of Toronto in Canada.

## 3.2. Randomization

Participants in the US and Canada are randomized 1:1 to fluvoxamine or matching placebo capsules. A study statistician will generate randomization schedules stratified by age and sex. Separate randomization tables were prepared for the US and Canadian sites. Treatments will be randomly allocated using randomly alternating blocks of sizes 2 and 4. Randomization allocation is conducted via REDCap, which displays randomization assignments to research team members who prepare study materials including drug or placebo but otherwise have no contact with participants. All individuals in contact with participants are blinded, including outcome assessors and investigators, as are participants themselves.

## 3.3. Sample Size

Our original proposal was to recruit 1100 participants in order to have 880 participants in the modified intention-to-treat sample. This sample will include all enrolled participants who were confirmed eligible at the baseline assessment and reporting taking at least one dose of study medication. A modified intention-to-treat strategy is required because participants will be shipped supplies and study medication (whereupon they are randomized) prior to their measuring oxygen saturation to confirm study eligibility.[1]

The planned recruitment number was adjusted to approximately 1100 participants in the mITT group mid-study due to the slow recruitment rate and low event rate in the mITT sample. This was probably because many at-risk adults in the US and Canada were vaccinated. Not only did the case rate drop considerably, but also the cases were mostly occurring among those either at low risk (who would not be eligible) or who were vaccine-hesitant (who may less likely be willing to participate in medical research). The DSMB recommended stopping the study on May 19th, 2021. The last enrollment date was May 21st, 2021, with a total of 669 participants randomized and 551 participants included in the mITT sample.

## 3.4. Framework

Hypothesis testing framework: $H_0$ and $H_1$ with confidence intervals for differences.

## 3.5. Statistical interim analyses and stopping guidance

A pre-planned interim analysis was conducted once the trial had recruited >50% of the originally planned number of participants for the planned main analysis. On the 4th DSMB meeting on May 10th, 2021, there had been 657 participants randomized, with 527 of the 1100 participants in the mITT sample (data

[1] In the preliminary study (STOP COVID Trial), approximately 20% of individuals who received study supplies were not in the intention-to-treat sample, mainly because study eligibility could not be confirmed.

presented as of May 5th, 2021). No safety signals were noted after reviewing adverse events and serious adverse events. While the study was initially designed under the assumption of an overall 16.5% event rate (20% in the placebo arm and 13% in the fluvoxamine arm), the overall event rate based on the data presented as of May 5th, 2021, was 5%. Conditional power under the original design was presented as 22%. The team proposed updated assumptions of 8% for the placebo group and 4% for the fluvoxamine group. With those updated assumptions, the necessary mITT sample size would be approximately 550 participants per arm, a more than 20% increase. The conditional power under the updated assumptions would be approximately 5%. The DSMB recommended the early stop, and a decision was made on May 19th, 2021.

### 3.6. Timing of final analysis

H1a and H1b analyses have ensued after we decided to early stop recruitment on May 21st, 2021, and after all the active enrolled participants reach day 15 in the RCT phase. H2 analysis will ensue after all enrolled participants complete day 90 follow-up surveys in the follow-up phase.

This statistical analysis plan was added to the study protocol at clinicaltrials.gov, before the closure of the database and before any analyses had been conducted. Independent study monitoring was conducted in adherence to the Good Clinical Practice (GCP) guidelines. The statistician will be blind to treatment assignments during the H1a and H1b analysis and unblind during H2 and exploratory analysis.

### 3.7. Timing of outcome assessment

Outcome assessments will be conducted at the end of the RCT phase on day 15. During the follow-up phase, assessments will be conducted on day 90.

## 4. Statistical principles

### 4.1. Confidence intervals and p values

To test our primary hypothesis (H1) and secondary hypothesis (H2), a significance level of .05 will be considered statistically significant. Results will be presented with their values (e.g., coefficients, standard deviation, etc.) with 95% confidence intervals.

### 4.2. Adherence and protocol deviations

#### 4.2.1. Definitions of protocol deviations

Any alteration or modification to the IRB-approved research will not be conducted without prospective IRB approval. The term research encompasses all IRB-approved materials and documents, including the detailed protocol, IRB application, consent form, recruitment materials, questionnaires/data collection forms, and any other information relating to the research study.

#### 4.2.2. Adherence and protocol deviations

As a pragmatic study, all levels of adherence are allowed. Compliance with the study-recommended medication and dosing will be followed during the trial. Reasons for non-compliance and protocol deviations will be documented.

#### 4.2.3. Electronic data capture

This study will utilize REDCap-based electronic Case Report Forms (e-CRF's) for data capture and storage. All study-related materials will be stored electronically in REDCap or a secured university server. No physical source documents are anticipated.

### 4.3. Analysis populations

We will follow the modified intention-to-treat (mITT) principle. In other words, participants who stop randomized medication will continue to be followed. Participants who meet the modified intent-to-treat principle will be included in the analysis. A per-protocol analysis will be performed for participants taking ≥80% doses of study medication until the time of deterioration or end of RCT phase.

# 5. Trial Population

## 5.1. Screening data

Potential participants identified through the IRB-approved recruitment plan will be screened for eligibility. Staff and PI's at each site are responsible for verifying that participants meet all eligibility criteria before being randomized to acute treatment. All sites in the US and Canada follow the same screening requirements.

## 5.2. Eligibility

All participants will meet the following eligibility criteria:

### 5.2.1. Inclusion Criteria

a) Men and women aged 30 and older.[2]

b) Not currently hospitalized.

c) Proven SARS-CoV-2 positive (per lab or physician report). A positive test[3] is within 3 days prior to or during the current symptomatic episode.

d) Currently symptomatic with one or more of the following symptoms: fever, cough, myalgia, mild dyspnea, chest pain, diarrhea, nausea, vomiting, anosmia (inability to smell), ageusia (inability to taste), sore throat, nasal congestion.

e) Expectation, on screening, that the participant will start study medication within 7 days of symptom onset.

f) Able to provide informed consent.

g) Upon initial screening, participant reports one of the following risk factors for clinical deterioration: age⩾40, racial/ethnic group African-American, Hispanic, South Asian, or Native American (including more than one race), or 1+ of the following medical conditions which increase the risk for developing moderate-severe COVID illness: obesity, hypertension, diabetes, heart disease (coronary artery disease, history of myocardial infarction, or heart failure), lung disease (e.g., Asthma, COPD), immune disorder (e.g., rheumatoid arthritis, lupus).

---

[2] Inclusion criteria was: Men and Women aged 18 and older. Adjusted after first DSMB call on Feb 8th,2021

[3] Due to the study nature, it is not possible to acquire a positive test confirmation from every participant at the time of the recruitment.

### 5.2.2. Exclusion Criteria

a) Illness severe enough to require hospitalization or already meeting study's primary endpoint for clinical deterioration[4] (e.g., current O2 saturation <92% on room air, current use of supplemental oxygen to maintain O2 saturation ⩾92%).

b) Unstable medical comorbidities (e.g., decompensated cirrhosis), per patient report and/or medical records.

c) Immunocompromised from the following: solid organ transplant, BMT, high dose steroids (>20mg prednisone per day), or tocilizumab.[5]

d) Already enrolled in another COVID 19 medication trial (not including vaccination or prophylaxis trials)

e) Unable to provide informed consent.

f) Unable to perform the study procedures.

g) Taking donepezil, or sertraline.

h) Taking warfarin-also known as Coumadin, phenytoin, clopidogrel, and St John's wort.

i) Taking SSRIs, SNRIs, or tricyclic antidepressants, unless these are at a low dose such that a study investigator concludes that a clinically significant interaction with fluvoxamine (i.e., either serotonin syndrome or TCA overdose) is unlikely.

j) Individuals who report they have bipolar disorder or are taking medication for bipolar disorder (lithium, valproate, high-dose antipsychotic) unless the investigator concludes that the risk for mania is unlikely.

k) Individuals who take alprazolam or diazepam and are unwilling to cut the medication by 25%.

l) Participants taking theophylline, tizanidine, clozapine, or olanzapine will be reviewed with a study investigator and excluded unless the investigator concludes that the risk to the participant is low.

m) Received vaccine for COVID-19.

n) Individuals who are already taking an approved or investigational COVID-19 treatment or have already received monoclonal antibody treatment or convalescent plasma.

## 5.3. Recruitment

A consort diagram will be used to visualize the flow of participants. We will report the number of eligible participants and the number of screened participants. The reasons for lack of eligibility will be enumerated. The number randomized to the mITT group will be reported as well as the reasons for non-randomized to mITT group. The number randomized into either arm (fluvoxamine vs. placebo) will be reported.

[4] participants who are randomized and then found to meet clinical deterioration criteria at baseline – or meet deterioration criteria prior to starting study medication - will (or may) remain in the study and be managed and assessed just as other randomized participants. They will not be included in the modified intention to treat sample during data analysis.

[5] Older version: Immunocompromised (solid organ transplant, BMT, AIDS, on high dose steroids (>20mg prednisone per day).

## 5.4. Withdrawal/follow-up

### 5.4.1. General withdrawal rules

Participants are free to withdraw from participation in the study at any time upon request.
The PI may discontinue or withdraw a participant from the study for the following reasons:

- The participant is not contributing data and/or appears to be lost to follow-up.
- If any clinical adverse event (AE), laboratory abnormality, or other medical condition or situation occurs such that continued participation in the study would not be in the best interest of the participant or interfere with the integrity of the study data.
- Disease progression which requires discontinuation of the study intervention
- If the participant meets an exclusion criterion (either newly developed or not previously recognized) that precludes further study participation

After completing day 15 in the RCT phase, participants will enter the follow-up phase till day 90.

## 5.5. Baseline participant characteristics

### 5.5.1. List of baseline characteristics

Participants will sign informed consent forms at baseline. Local research team assessments elements collected during baseline:

- Temperature
- Oxygen level
- Blood pressure
- Pulse
- Self-assessed symptoms
  - Fever
  - Cough
  - Shortness of breath
  - Fatigue/Weakness
  - Chills
  - Sore throat
  - Nasal congestion
  - Nausea
  - Body aches
  - Diarrhea
  - Loss of appetite
  - Loss of smell
  - Loss of taste
- Medical history

The key variables being used in the study include:

- Age
- Gender
- Race
- Sites
- Days from symptom onset to start trial medication
- BMI
- Coexisting conditions(medical history)
- Self-assessed symptoms

- Oxygen level
- Randomization
- Endpoint for clinical worsening
- Days in RCT phase free of clinical deterioration
- WHO COVID-19 Therapeutic Trial Synopsis 9-point scale

### 5.5.2. Descriptive summarization of baseline participant characteristics

We will list general patient characteristics in a baseline characteristics table. Continuous data will be presented as the mean with standard deviation (SD), median with interquartile range, and range(min-max). Dichotomous and categorical data will be presented in proportions.

# 6. Analysis

## 6.1. Outcome definitions

### 6.1.1. Primary and secondary outcomes

Primary: Participants who have a ① presence of dyspnea and/or hospitalization for shortness of breath or pneumonia, and ② $O_2$ saturation decrease (<92% on room air via the twice-daily at-home self-monitoring or via monitoring at a hospital/ER/urgent care center, and/or supplemental oxygen requirement to keep O2 saturation ⩾92%) will be clinically deteriorated. The numerical score of the Modified WHO COVID-19 Therapeutic Trial Synopsis 9-point scale will be used for analysis.

Secondary: Secondary outcome will compare post-COVID functioning at day 15 and day 90 using the PROMIS 10-item Global Health Scale. It reflects the increasing concern regarding the persistence of COVID-19 illness and functional impairment in a substantial percentage of individuals with initially "mild" symptoms. We will also explore persistent symptoms of COVID-19 at these time points.

### 6.1.2. Outcome measurements

Primary outcome (clinical deterioration) will be measured by ① an SOB rating of 4+ on the twice-daily 0-10 scale and/or hospitalization for shortness of breath or pneumonia during study day 1- day 15 and ② $O_2$ readings <92% on room air via the twice-daily at-home self-monitoring or monitoring at a hospital/ER/urgent care center, and/or supplemental oxygen requirement to keep $O_2$ saturation ⩾92% during study day 1- day 15.

The secondary outcome will be measured by a PROMIS 10-item Global Health Scale on study day 15 and day 90.

Four exploratory analyses will be performed. First, we will examine the difference between the fluvoxamine group vs. placebo group and the deterioration group vs. non-deterioration group in study medication adherence. Second, we will explore co-prescribed medications to look for patterns of difference between the fluvoxamine and placebo groups. Third, we will examine whether the persistent symptoms reported on day 15 and day 90 vary between the fluvoxamine and placebo groups. Fourth, we will examine the difference of symptom onset days (from symptom onset to start of medication) between the fluvoxamine and placebo group, as well as between deteriorated and non-deteriorated groups.

In addition, we will examine the outcomes of randomized participants discovered to meet the study endpoint of clinical deterioration at baseline. We will examine whether those randomized to fluvoxamine have lower hospitalization rates, ventilator care, and mortality, and we will gather the same outcomes at day 15 and day 90 post-randomization.

## 6.2. Analysis methods

To test hypothesis H1a (clinical deterioration), survival analysis with the log-rank statistic stratified by the US vs. Canada, gender, and age group will be performed testing between the two treatment groups. Participants who meet the primary outcome will represent an event. The WHO COVID-19 Therapeutic Trial Synopsis 9-point scale will be compared between the two groups with a t-test. We will also summarize the distribution of subjects at each level of the scale.

To test hypothesis H1b (treatment effect size), a Cox proportional hazard model will fit the data using strata as in H1a with factors as the US vs. Canada, gender, and age group, baseline $O_2$ saturation, and their interaction. The test for moderation would be a test of the interaction between treatment and baseline $O_2$ saturation (dichotomized with a median split).

To test hypothesis H2 (post-COVID functioning), an ANOVA test stratified by the US vs. Canada, gender, and age will be performed for day 15 and day 90. Differences between treatment groups will be tested.

In terms of the exploratory outcomes, descriptive statistics with 95% confidence intervals will be computed. If the prevalence of persistent symptoms is adequate, we will explore post-hoc regression models which predict persistent symptoms based on the duration and severity of the particular symptom during the acute phase and other clinical features during the acute phase. If we find co-prescribed medication differences between two groups, a further examination on medication interaction effect will be performed. We will also conduct per-protocol analysis, using only those participants who took ≥80% of study drug up until the time of deterioration or the end of 15-day trial.

## 6.3. Missing data

Missing data can occur from participant dropout/withdrawal (unit non-response), meeting study endpoint, failure to provide data (item non-response), or administrative issues. We will minimize missing data by closely monitoring accumulating data for missingness and implementing data quality control (QC) checks to reduce it when identified. Any missing data or anomalies will be communicated to the site(s) for clarification/resolution. When a participant drops out of the study, we will document the reason for dropout and who made the dropout decision. No imputation for missing data will be used for H1a and H1b analysis. We will use a multiple imputation analysis to estimate potential bias from missing data.

## 6.4. Harms

During the study, serious adverse events (SAE) and adverse events (AE) will be monitored continuously throughout the study and will be reported to the DSMB and IRB according to pre-specified requirements. SAE/AE rates and interim study results will be reviewed and discussed by the DSMB at the DSMB meetings.

## 6.5. Statistical software

Data cleaning and statistical analyses will be performed using SAS (SAS Institute, Cary NC) and R (The R Foundation for Statistical Computing; Vienna, Austria).

**Amendments to Statistical Analysis Plan**

**Clarification re: stratification:** Throughout the SAP, it is indicated that the analyses will be stratified by country (e.g., US vs. Canada). Randomization stratification was based on a 5 site variable (i.e., Washington University, Northwestern University, University of Utah, Fred Hutchinson Center Clinical Research Center, plus Canada sites); given this, it was determined models will include site as a 5-level factor.

**Major Changes to the Statistical Analysis Plan Over the Course of the Study**
Below we summarize major changes and points of clarification made to the Statistical Analysis Plan over the course of the trial.

**2.2.1 Objectives and study aims**

- **World Health Organization (WHO) COVID-19 Therapeutic Trial Synopsis Scale**: Added the use of a modified version of the WHO 9-point scale as a measure of clinical severity at its worst.
- **Adherence, Persistence of Clinical Symptoms, and Difference of Symptom Onset Days**: Added that we will explore the following:
    - Study medication adherence between the fluvoxamine and placebo group
    - Difference of persistent clinical symptoms at day 15 and day 90 between fluvoxamine and placebo group
    - Difference of symptom onset days (from symptom onset to start of medication) between fluvoxamine and placebo group, and between deteriorated and non-deteriorated group.
- Note: Due to early stop of trial as a result of futility, we did not explore whether the benefits of fluvoxamine for preventing deterioration varies as a function of baseline O2 saturation as indicated in the original Statistical Analysis Plan.

**3.2. Randomization**

- **Randomization Tables:** Clarified that separate randomization tables were prepared for the US and Canadian sites.

**3.3 Sample Size and 3.5 Statistical interim analyses and stopping guidance**

- **Stop of Study**: Included the recommendation of the DSMB to stop the study.

**4.3 Analysis populations**

- **Sensitivity Analysis:** No sensitivity analysis was conducted as originally planned.
- **Per-Protocol Analysis:** Added that Participants who meet the modified intent-to-treat principle will be included in the analysis. A per-protocol analysis will be performed for participants taking ≥80% doses of study medication until the time of deterioration or end of RCT phase.

**6.1.2. Outcome measurements**

- **Exploratory Analyses:** Clarified the four exploratory analyses to be conducted. We state:
    - "Four exploratory analyses will be performed. First, we will examine the difference between the fluvoxamine group vs. placebo group and the deterioration group vs. non-deterioration group in study medication adherence. Second, we will explore co-prescribed medications to look for patterns of difference between the fluvoxamine and placebo groups. Third, we will examine whether the persistent symptoms reported on day 15 and day 90 vary between the fluvoxamine and placebo groups. Fourth, we will examine the difference of symptom onset days (from symptom onset to start of medication) between the fluvoxamine and placebo group, as well as between deteriorated and non-deteriorated groups."

**6.2 <u>Analysis methods</u>**

- **WHO COVID-19 Therapeutic Trial Synopsis 9-point scale:** Added analysis method. We state:
  - "The WHO COVID-19 Therapeutic Trial Synopsis 9-point scale will be compared between the two groups with a t-test. We will also summarize the distribution of subjects at each level of the scale."
- **Moderation test for the interaction between treatment and baseline (x saturation)**
  - Note: It was determined this was not necessary to do as there was not an effect of fluvoxamine overall; this interaction analysis would likely be underpowered.
- **H2 (post-COVID functioning):** Clarified that difference (not pairwise comparisons) would be tested.
- **Exploratory Analyses**
  - **Descriptive Statistics:** Added that 95% confidence intervals would be computed.
  - **Co-prescribed medications**: Added that if differences were found between the two groups, a further examination on medication interaction effect would be performed.
  - **Per-protocol Analysis**: Added that conduct per-protocol analysis, using only those participants who took ≥80% of study drug up until the time of deterioration or the end of 15-day trial.

**<u>Additional Amendments</u>**

- **Clarification re: stratification:** Throughout the SAP, it is indicated that the analyses will be stratified by country (e.g., US vs. Canada). Randomization stratification was based on a 5 site variable (i.e., Washington University, Northwestern University, University of Utah, Fred Hutchinson Center Clinical Research Center, plus Canada sites); given this, it was determined models will include site as a 5-level factor.
