# Supplementary material for: The STOP COVID 2 Study: Fluvoxamine vs Placebo for Outpatients With Symptomatic COVID-19, a Fully Remote Randomized Controlled Trial
Source: Open Forum Infect Dis. 2023 Aug 8;10(8):ofad419. doi: 10.1093/ofid/ofad419 (PMC10445518; doi:10.1093/ofid/ofad419)
Supplement: ofad419_Supplementary_Data [file ofad419_supplementary_data.zip › STOP COVID 2 Protocol_First, final, and summary of changes.pdf]

FLUVOXAMINE FOR EARLY TREATMENT OF COVID-19: A FULLY-REMOTE, RANDOMIZED PLACEBO CONTROLLED TRIAL

Principal Investigator: Eric Lenze, MD and Angela Reiersen, MD

Sponsor: Washington University School of Medicine

Funded by: COVID-19 Early Treatment Fund, NIAID (pending)

Version Number: v. 1

December 1, 2020

1 PROTOCOL SUMMARY

1.1 SYNOPSIS

Title: FLUVOXAMINE FOR EARLY TREATMENT OF COVID-19: A FULLY-REMOTE, RANDOMIZED PLACEBO CONTROLLED TRIAL

Study Description: This clinical trial will test a promising drug for early treatment of COVID-19 in 880 adults who are SARS-CoV-2 positive and recently infected. The trial will determine whether fluvoxamine (1) prevents clinical deterioration (defined as the combination of shortness of breath and hypoxia), and (2) improves short- and long-term functional outcome. Clinical deterioration is the primary endpoint. Fluvoxamine is an SSRI (FDA approved for OCD and Social Anxiety Disorder) that also activates the sigma-1 receptor (an immunomodulatory receptor). It prevented clinical deterioration in a preliminary RCT (called STOP COVID). Fluvoxamine (or placebo) will be given for approximately 15 days post-randomization, during which participants will self-report on shortness of breath and oxygen saturation via a twice-daily survey. At the end of approximately 15 days, fluvoxamine (or placebo) will be stopped. An additional survey at approximately day 15 and approximately day 90 will assess function.

Objectives:

The primary objective of this clinical trial is to test whether fluvoxamine prevents clinical deterioration in early COVID-19 illness.

The primary outcome will be clinical deterioration, defined as both of the following: (1) presence of dyspnea and/or hospitalization for shortness of breath or pneumonia, plus (2) decrease in O_2 saturation (<92% on room air and/or supplemental oxygen requirement to keep O_2 saturation ≥92%).

The hypotheses are:

Participants randomized to fluvoxamine will have a lower rate of clinical deterioration, compared to placebo.

A secondary objective of this clinical trial is to test whether fluvoxamine improves 15-day and 90-day functional outcome in early COVID-19 illness.

Secondary outcome will be post-COVID functioning at approximately 15 days and at approximately 90 days post-randomization using the PROMIS 10-item Global Health Scale. This secondary outcome reflects the increasing concern regarding persistence of COVID-19 illness and functional impairment in a substantial percentage of individuals with initially “mild” symptoms. We will also explore persistent symptoms of COVID-19 at these timepoints.

The hypotheses are:

Participants randomized to fluvoxamine will have higher post-COVID functional status at day 15 and day 90, compared to placebo.

Another secondary objective is to test whether baseline oxygen saturation moderates (modulates the drug-placebo difference) fluvoxamine's effect on clinical deterioration.

The hypothesis is:

Participants with baseline O_2 saturation ≤96% will have a larger treatment effect size (i.e., larger fluvoxamine-placebo difference in rate of clinical deterioration) than those with O_2 saturation ≥97%.

The basis for this hypothesis is an exploratory analysis of the preliminary RCT which found that all cases of clinical deterioration (26% of the placebo group) occurred in participants whose baseline oxygen saturation was 96% or less.

**Endpoints:**	Primary Endpoint: Clinical Deterioration<br>Secondary Endpoints: PROMIS Global Health Scale
**Study Population:**	Not currently hospitalized, participants age 18 and older who are SARS-CoV-2 positive and symptomatic with onset <7 days.
**Phase:**	Phase 3
**Description of Sites/Facilities Enrolling Participants:**	Washington University School of Medicine in St. Louis<br>Northwestern University<br>University of Utah<br>Fred Hutchinson Center Clinical Research Center
**Description of Study Intervention:**	Fluvoxamine Maleate, 100mg twice daily x 15 days
**Study Duration:**	Up to 3 months
**Participant Duration:**	Up to 3 months

1.2 SCHEMA

Diagram 1: Randomized Clinical Trial

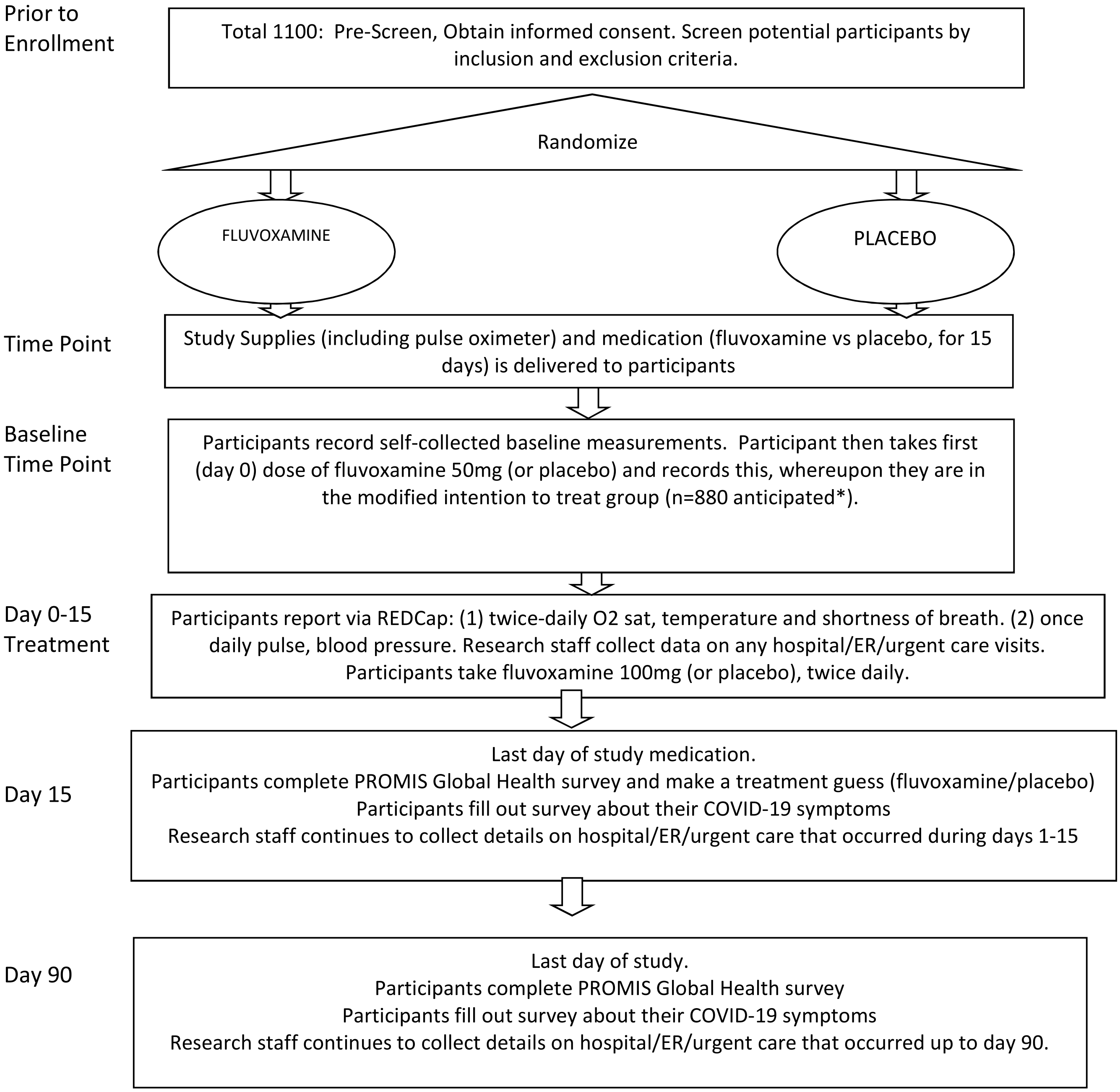

*Note this number (n=880) refers to the modified intent to treat sample: all participants for whom study eligibility is met at baseline and who take at least one dose of study medication. All data analyses will be conducted using this sample. The larger number n=1100 refers to the estimated number who will receive study medication (and therefore are considered randomized) in order to achieve n=880 in the modified intent to treat sample.

1.3 SCHEDULE OF ACTIVITIES (SOA)

**Procedures**	Screening Day -5 to 0	Enrollment/Baseline	Treatment days 0-15	Day 15 Treatment	Day 90 Post Treatment
Informed consent	X				
Demographics	X				
Medical history	X				
Randomization	X				
Administer study intervention		X			X
Twice-daily REDCap survey to assess oxygen saturation, self-reported dyspnea, vital signs, and treatment adherence	X---X				
Assessment of details of COVID-19 care received (e.g.. hospitalization, ER, or urgent care visit; length of stay; requirement for supplemental oxygen, ventilator, or other organ support; mortality)	X--X				X
Adverse event review and evaluation	X--X				X
Treatment guess (patient guesses whether they took fluvoxamine vs placebo)				X	

2 INTRODUCTION

2.1 STUDY RATIONALE

2.2 BACKGROUND

Coronavirus disease 2019 (COVID-19), caused by the novel severe acute respiratory syndrome coronavirus 2 (SARS-CoV-2), can cause serious illness including lung damage, hypoxia, and cardiac damage, which can lead to hospitalization, intensive care unit (ICU) admission, and death.[1]

Clinical deterioration often occurs during the second week of illness. Early investigations of COVID-19 found that serious illness leading to hospital admission occurred a median of 8-10 days after initially mild to moderate symptoms.[2-4] Evidence that many patients with COVID-19 develop lung damage from an excessive inflammatory response led to recommendations to repurpose immunomodulatory drugs to counter this hyperinflammation.[5,6] More recent evidence supports the assertion that the inflammatory reaction, including elevations in cytokines such as IL-6, predicts clinical deterioration.[7,8]

Many patients have debilitating persistent symptoms and loss of function, even three or more months after the initial stage of illness. The causes of this persistent illness are unclear and likely heterogeneous; they include persistence of the viral infection, immunological changes, and exacerbation of underlying medical conditions.[9] As a result, both anti-viral and immunomodulatory drugs could potentially prevent or reduce this post-COVID functional impairment.

Fluvoxamine, an SSRI, repurposed for early treatment of COVID-19

Fluvoxamine is unique among the SSRIs in that it strongly activates the sigma-1 receptor (S1R), and preclinical studies have suggested that by this mechanism fluvoxamine could prevent the immunopathology seen in serious COVID-19. In 2019, it was shown that fluvoxamine given early in treatment, reduced deterioration and mortality in two animal models of sepsis (bacterial peritonitis and lipopolysaccharide stimulation).[10] The same study demonstrated that fluvoxamine was anti-inflammatory in human cells, reducing IL-6 and other cytokines in lipopolysaccharide-induced inflammation. This study is part of an increasing body of research supporting the S1R in protection from deleterious consequences of inflammation.[11-13]

Fluvoxamine has one of the strongest S1R agonist effects of any existing medication.[14,15] It is also highly lipophilic and has rapid, substantial intracellular uptake.[16] Fluvoxamine also has many pragmatic advantages for repurposing, including ease of use, high safety margin, good tolerability, wide availability, and low cost. We determined that, if it could be shown that fluvoxamine had a beneficial effect in reducing clinical deterioration of COVID-19, the drug (and other S1R agonists) could be an important addition to the armamentarium vs. this pandemic.

Therefore, we conducted a double-blind, placebo-controlled RCT to test whether fluvoxamine, given as early treatment in individuals with mild COVID-19 illness, might prevent clinical deterioration. [49]

From April 10-Sept 19, 2020, we conducted the STOP COVID trial (ClinicalTrials.gov Identifier: NCT04342663), a preliminary single-site trial conducted in Missouri and Illinois, to test whether a course of fluvoxamine given early in COVID-19 illness could prevent clinical deterioration. Participants were recruited mainly from review of electronic medical records to identify individuals with a recent positive SARS-CoV-2 PCR test. Based on review of the medical record, potentially eligible individuals were contacted to confirm eligibility criteria and offer participation in the study. The study was fully-remote, with no in-person contact with participants.

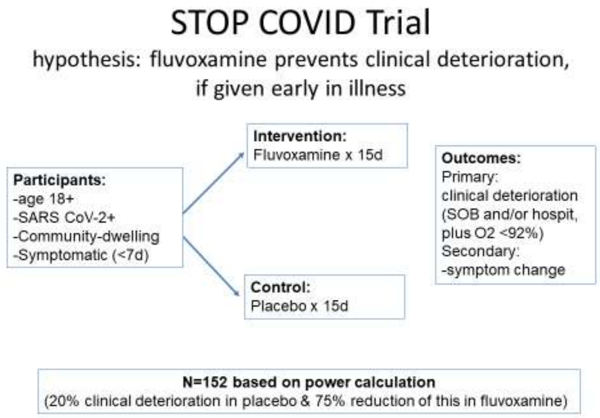

The STOP COVID participant flow diagram is shown along with participant baseline characteristics (*see Figure and Tables on next page*). Participants were randomized to receive fluvoxamine 100 mg vs. placebo up to three times daily. Study medication and monitoring equipment (fingertip pulse oximeter, blood pressure cuff, thermometer) were delivered directly to participant homes. Participants entered their own O_2 saturation, vital signs, and symptom rating data twice daily for 15 days online or via phone into REDCap surveys, which were monitored daily by study staff. Participants were contacted as needed to clarify symptom and vitals/ O_2 saturation (e.g., when participants reported an out-of-bounds value or one indicating hypoxia). This monitoring included study staff, under study physician supervision, advising participants to contact their physician or visit an emergency room when appropriate. The primary study endpoint was time to clinical worsening, which was defined as 1) dyspnea or hospitalization for shortness of breath or pneumonia and 2) O_2 saturation <92% or requirement for supplemental O_2 to keep saturation ≥92%.

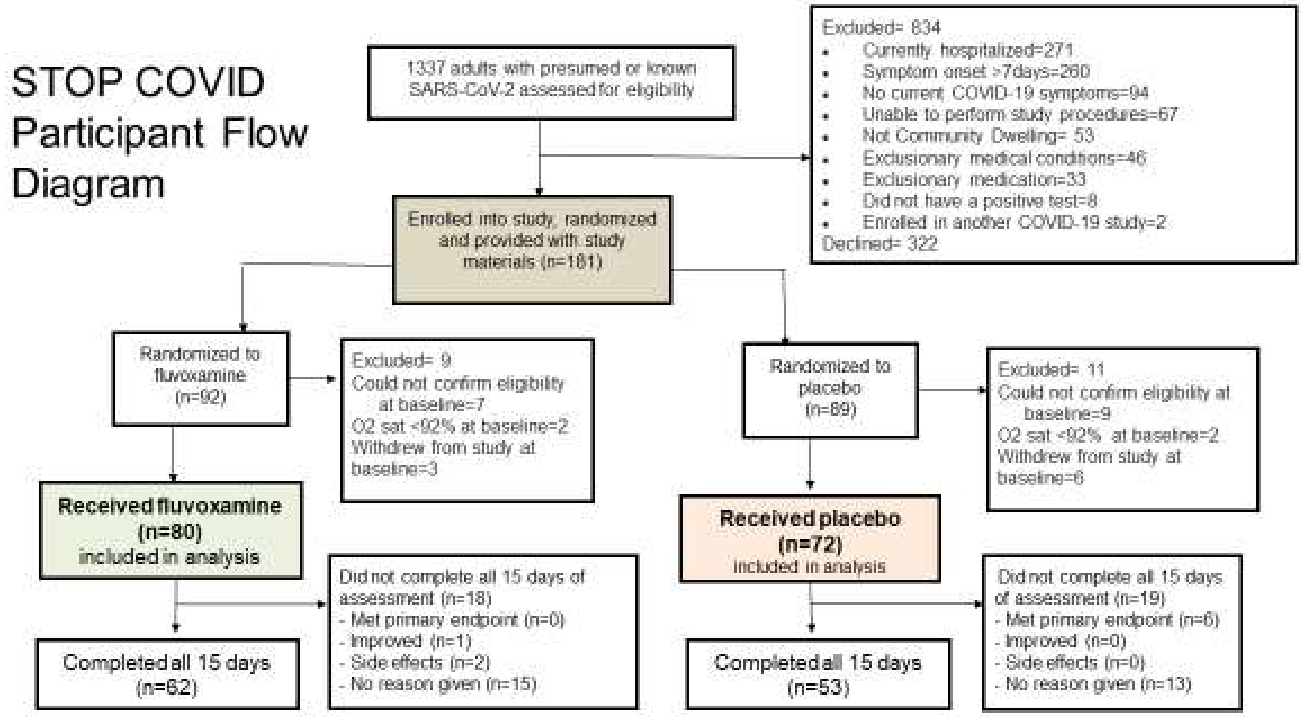

STOP COVID DEMOGRAPHICS

Characteristics	Fluvoxamine (n=80)	Placebo (n=72)
Sex at birth, No (%)		
Female	56 (70%)	53 (73.6%)
Male	24 (30%)	19 (26.4%)
Race, No (%)		
White/Caucasian	56 (70%)	50 (69.44%)
Black/African American	18 (23.1%)	20 (27.0%)
Asian	3 (3.9%)	1 (1.4%)
Other	2 (2.6%)	1 (1.4%)
Unknown	1 (1.3%)	0
American Indian/Alaska Native	0 (0%)	1 (1.4%)
Ethnicity, No (%)		
Non-Hispanic/Non-Latino	75 (93.8%)	66 (91.7%)
Hispanic/Latino	3 (3.9%)	2 (2.8%)
Unknown/not reported	2 (2.6%)	4 (5.4%)

STOP COVID BASELINE CHARACTERISTICS

Characteristics	Fluvoxamine (n=80)	Placebo (n=72)
Age(year)		
Median (IQR)	46 (35-58)	45 (36-54)
Range	20 – 75	21 – 69
Duration of COVID-19 symptoms*, days		
Median (IQR)	4 (3-5)	4 (3-5)
Range	1 - 7	1 - 7
Most severe COVID-19 symptom at baseline*, No (%)		
Loss of smell	26 (32.5%)	18 (25%)
Fatigue	17 (21.3%)	18 (25%)
Body aches	9 (11.3%)	13 (18.1%)
Cough	9 (11.3%)	1 (1.4%)
Subjective fever	8 (10%)	4 (5.6%)
Loss of appetite	3 (3.8%)	8 (11.1%)
Chills	3 (3.8%)	6 (8.3%)
Shortness of breath	2 (2.5%)	1 (1.4%)
Loss of taste	2 (2.5%)	2 (2.8%)
Nausea	1 (1.3%)	1 (1.4%)
Oxygen saturation		
Median (IQR)	97% (96-98%)	97% (96%-98%)
Range	93% - 99%	92% - 99%

The key finding from STOP COVID was that 0% (0/80) of fluvoxamine-treated individuals suffered clinical deterioration, vs. 8.3% (6/72) of those who received placebo (*Figure, right*). In this study, fluvoxamine was generally well-tolerated. The fluvoxamine group had 1 serious adverse event and 11 other adverse events, while the placebo group had 6 serious and 12 other adverse events. Further, we collected 30-day follow-up data (after the 15-day RCT phase): 1 individual in each group received ER or hospital care (1 placebo for chest pain, 1 fluvoxamine for headache) and no individuals clinically deteriorated. This demonstrated that fluvoxamine prevented, rather than merely delayed, deterioration.

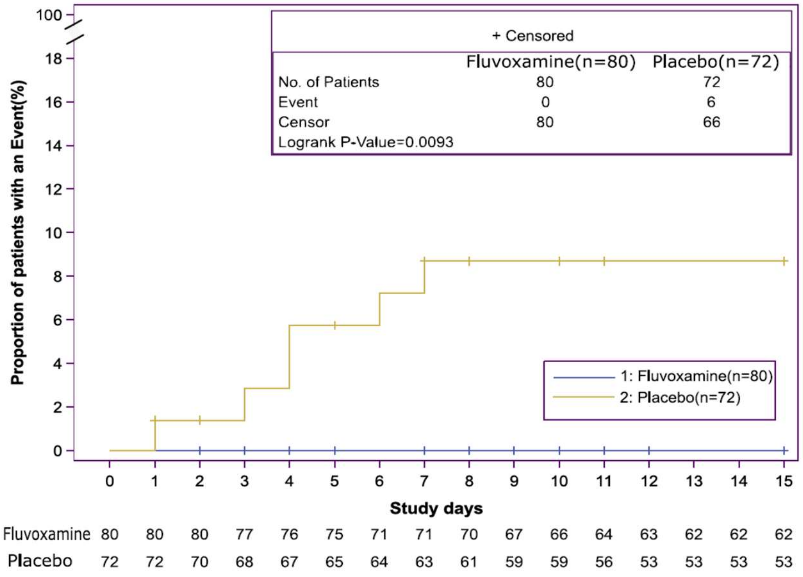

This preliminary study demonstrates the feasibility of conducting a fully-remote COVID-19 early treatment trial, with first dose of medication an average of 4 days after onset of symptoms. A fully-remote design has been demonstrated to be an efficient, pragmatic technique for conducting COVID-19 early treatment trials, as demonstrated by this study as well as the hydroxychloroquine studies led by University of Minnesota.

Baseline O_2 may be a moderator of fluvoxamine's treatment effect size. As the figure to the right shows, we found that low-normal baseline O_2 saturation may be a risk factor for clinical deterioration and a moderator of fluvoxamine's treatment effect size. In post-hoc analyses, we examined baseline (pre-treatment) O_2 saturations in the 152 participants. *All* of the clinical deterioration occurred in individuals with baseline O_2 saturation of ≤96%: 6/23 (26%), compared to 0/49 (0%) in those with baseline O_2 saturation ≥97%. This is important because it suggests that fluvoxamine could be given to individuals with early COVID-19 only if and when their O_2 saturation falls to 96% or less.

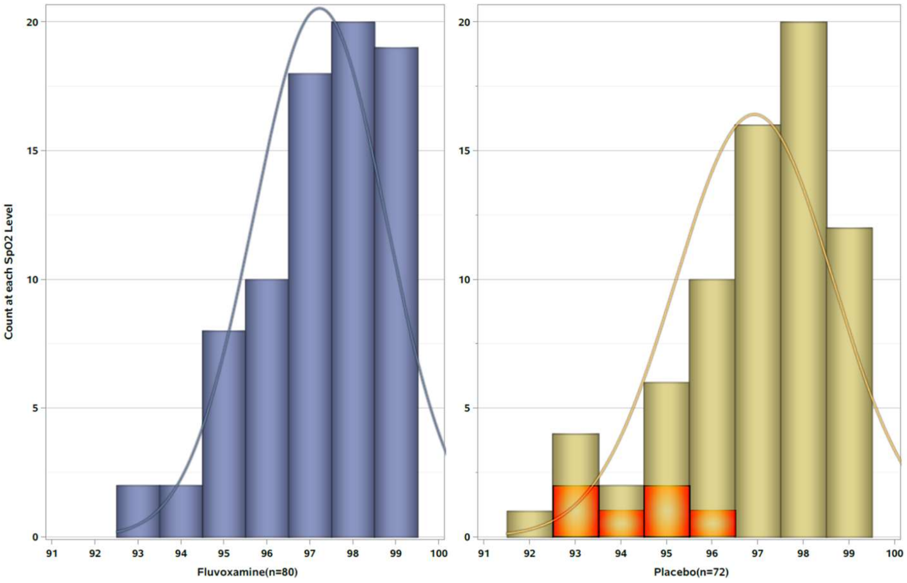

Anti-inflammatory (cytokine reduction) actions resulting from S1R activation would fit with recent findings of benefits of other anti-inflammatory drugs for COVID-19, including colchicine and corticosteroids.[17,18] However, a recent study found lower levels of cytokines in severe COVID-19 compared to bacterial sepsis.[19] If fluvoxamine's benefit is a S1R-mediated effect, this drug could be utilized not only to reduce morbidity and mortality in COVID-19, but also in other medical conditions in which treatments to dampen immunopathology are relevant – including future pandemics.

There were, however, several limitations to this study. First, this was a single-site study with a small sample size; the small sample size also results in wide confidence intervals (i.e., fluvoxamine appeared 100% effective at preventing clinical deterioration, but true effect size may be much lower than this). Second, we did not enrich the sample for risk factors for deterioration (such as older age); as a result, the rate of deterioration was low (8.3%) in the placebo group; this led to a fragile study finding.

Third, we attempted to measure symptoms of COVID-19 as a treatment outcome, by asking about most severe symptom at baseline and then measuring change in that symptom. This method proved to be invalid for several reasons. First, the presenting symptoms were highly heterogeneous as demonstrated in the Table above. Second, we conducted several advanced data analytic techniques focusing on latent trajectory modelling, and we found that the temporal dynamics of COVID-19 symptoms were highly heterogeneous. In other words, while symptoms overall decelerated (declined non-linearly over time), the trajectories of symptoms varied greatly between participants. This great heterogeneity of symptoms and their time course presents a challenge for the assessment of symptom change in clinical trials.[20] A validated measure of functional status may be more robust for assessing COVID-19 persistence; however, we did not include such a measure in STOP COVID. Additionally, a longer-term outcome is needed (beyond 15 days) to assess persistence of COVID-19.

Fluvoxamine Possible Mechanisms of Action

Fluvoxamine is a selective serotonin reuptake inhibitor (SSRI) and a S1R agonist.[21] The preliminary study was motivated by a hypothesis that S1R agonists could dampen excessive inflammation in COVID-19. This and other potential mechanisms by which fluvoxamine might improve outcomes in COVID-19 are summarized below.

1) Anti-inflammatory effects though the S1R-IRE pathway. The S1R is an endoplasmic reticulum (ER) chaperone protein involved in many cellular functions, including regulation of the ER stress response/unfolded protein response (UPR) and inflammation.[22] S1R has been shown to inhibit ER stress sensor inositol-requiring enzyme 1α (IRE1) mediated splicing of XBP1[23], a master regulator of cytokine production. These anti-inflammatory effects may be the most likely explanation for fluvoxamine's beneficial effects. In COVID-19, an excessive inflammatory process known as "cytokine storm" may contribute to worsening of symptoms and cardiopulmonary complications, which can sometimes occur around the second week of illness. Fluvoxamine may dampen this excessive inflammatory response.

In a 2019 study by Rosen and colleagues, fluvoxamine showed benefit in preclinical models of inflammation and sepsis.[10] In one model, mice were exposed to the Toll-like receptor 4 (TLR4) ligand lipopolysaccharide (LPS), which can trigger an inflammatory response. In another model, a fecal slurry was injected, which triggers a usually sub-lethal infection and an inflammatory response. Mice lacking the S1R showed excessive increases in cytokine levels and greatly reduced survival under either of these conditions, suggesting the S1R inhibits excessive inflammatory responses. Wildtype mice exposed to the same inflammatory triggers showed reduced cytokine levels and increased survival when treated with fluvoxamine (a S1R agonist). When investigating the underlying mechanism of this effect, the authors demonstrated that S1R inhibits activity of IRE1, which in turn prevents the excessive cytokine production. In an experiment using human peripheral blood, they also showed that fluvoxamine can reduce LPS-induced cytokine production by human cells. In the case of COVID-19, fluvoxamine's S1R agonist action may have a similar ability to reduce the excessive inflammatory response induced by the viral infection, thus reducing inflammation-mediated organ damage.

2) Anti-viral action through effects on lysosomes, autophagy and/or endocytosis. Coronaviruses utilize cathepsin proteases present in the late endosome to facilitate cell entry[24] and re-model ER and phagosomal membranes into sites of viral replication.[25] Both of these processes require stimulating the endocytosis and phagosome arms of autophagy and then terminating autophagy before lysosomal fusion. SARS-CoV-2 proteins Nsp6, Nsp2, Orf7b, and Orf9b have been shown to localize with and modulate components of the autophagy pathway.[26,27] Further Nsp6 has been shown to physically associate with S1R.[28] Critically, S1R not only drives early stage autophagy through the IRE1/UPR pathway, but is also essential for lysosomal fusion to complete autophagy, likely by chaperoning components of the SNARE complex.[29] We hypothesize activation of S1R with fluvoxamine may overcome Nsp6 inhibition of S1R to allow autophagy to clear SARS-CoV-2. Others have also recognized targeting the autophagy pathway as a promising strategy to treat SARS-CoV-2.[30,31]

Chemically, fluvoxamine is a cationic amphiphilic drug (CAD) with a log P 3.1 and a pKa 9.4 and, along with a range of antipsychotic and antihistamine drugs, preferentially accumulates in the lysosome. Perhaps due to this, fluvoxamine achieves higher concentrations in the lungs (which are lysosome-rich) than in the brain.[32] In the case of COVID-19, this may enhance treatment effects in the airway epithelium.[33] At high doses (10 uM), CADs including fluvoxamine, have

been shown to inhibit lysosomal acid sphingomyelinase[34] to cause drug-induced phospholipidosis. This non-specific activity can globally dysregulate lipid homeostasis, which in turn modulates autophagy through the mTOR nutrient sensing pathway.[35]

3) Antiviral effects and prevention of organ damage through regulation of the ER stress response/UPR. Some viruses hijack the ER stress response/UPR to achieve viral functions[36,37], and a number of studies have suggested drugs targeting the ER stress response/UPR may be beneficial in treating COVID-19.[28,38] S1R agonists (such as fluvoxamine) regulate ER stress. S1R ligand effects on ER stress and other ER functions may reduce organ dysfunction/damage.[39,40]

4) Antiplatelet Effects (common to all SSRIs). Hyperactivity of platelets may contribute to pathophysiological processes leading to thrombotic complications in COVID-19.[41] SSRIs can inhibit platelet activation, which may reduce the risk of thrombosis, and these anti-platelet effects may be cardioprotective.[42]

5) Elevation of melatonin levels in the body. The SARS-CoV-2 virus may activate the NLRP3 inflammasome[43], which may contribute to cytokine storm.[44] Melatonin may act on this NLRP3 pathway to reduce inflammation.[45,46] Fluvoxamine inhibits the metabolism of melatonin, so can increase the melatonin level in the body[47], which may be beneficial in COVID-19.

In summary: We need to replicate (or refute) these preliminary findings in a larger confirmatory trial. We need to find out: (1) can fluvoxamine prevent clinical deterioration in early COVID-19? (2) Can fluvoxamine mitigate the long-term impact of COVID-19? (4) Do the benefits of fluvoxamine for preventing deterioration vary as a function of baseline oxygen saturation?

2.3 RISK/BENEFIT ASSESSMENT

2.3.1 KNOWN POTENTIAL RISKS

Potential risks associated with study assessments

There are no risks associated with any of the assessments.

POTENTIAL RISKS ASSOCIATED WITH STUDY MEDICATION

Fluvoxamine:

General comments: Fluvoxamine is an antidepressant drug that functions as a selective serotonin reuptake inhibitor (e.g., similar to escitalopram (Lexapro), fluoxetine (Prozac), sertraline (Zoloft), etc – among the most commonly prescribed drugs in the US). Its risk profile below is for chronic use in a psychiatrically ill population; the risks for short-term use in a non-psychiatric population are likely lower, as our initial STOP COVID trial demonstrated (18 total AEs in 80 fluvoxamine-randomized participants, compared to 19 AEs in 72 placebo participants). The research team will carefully evaluate co-prescribed drugs as well as OTC medications and will instruct participants to minimize caffeine (which has its metabolism inhibited by fluvoxamine), to mitigate drug-drug interactions.

Likely risks: none.

Less likely (1-10%): Nausea, Vomiting, Weight loss, Yawning, Loss of appetite, Agitation/Nervousness/Anxiety, Insomnia, *Somnolence*, *Tremor*, Headache, Dizziness, *Palpitations*, *Tachycardia* (high heart rate), Abdominal pain,

Dyspepsia (indigestion), *Diarrhea*, *Constipation*, *Hyperhidrosis* (excess sweating), *Asthenia* (weakness), *Malaise*, *Sexual dysfunction* (including delayed ejaculation, erectile dysfunction, decreased libido, etc.), *Xerostomia* (dry mouth).

Rare (<1%): *Arthralgia*, Hallucination, Confusional state, Extrapyramidal side effects (e.g. dystonia, parkinsonism, tremor, etc.), *Orthostatic hypotension*, Cutaneous hypersensitivity reactions (e.g. edema [buildup of fluid in the tissues], rash, pruritus), *Mania* (elevated mood together with reduced need for sleep and increased energy), seizures, Abnormal hepatic (liver) function, Photosensitivity (being abnormally sensitive to light), Galactorrhea (expulsion of breast milk unrelated to pregnancy or breastfeeding).

Minimizing risk of fluvoxamine in this study:

Fluvoxamine has been used for the treatment of depression and anxiety disorders worldwide since the 1990s. Its safety profile is well-known from millions of patient-years of use. It is not fatal even in overdose[48] and it does not prolong the QTc interval unlike other SSRIs (especially citalopram). It requires no therapeutic drug level monitoring or safety labs for its use. Its use in this study is within FDA-approved dosing; and it is only used for approximately 15 days. As well, all participants receive an initial dose of 50mg, and based on tolerability with this initial dose and in the first day(s) of intervention, the dose can be adjusted as needed to minimize side effects. Further, monitoring will include oversight by physicians with expertise in this drug class. Study exclusions will minimize the risk of clinically significant drug:drug interactions or other problems.

Other risks

There may also be unknown risks**.** We will adjust the informed consent process if new risks are identified.

2.3.2 KNOWN POTENTIAL BENEFITS

Although the study hopes to prevent serious symptoms of COVID-19, there is no guarantee that the study participants will receive any clinical benefits by participating in the study. All participants regardless of study randomization, will receive increased monitoring of their COVID-19 illness, potentially leading to management (e.g., being told to contact their provider or go to the ER) that would not have occurred otherwise or as promptly without this monitoring.

Importance of the Knowledge to be Gained

COVID-19 is a US-wide and worldwide public health emergency. This study may result in effective treatment that reduces its morbidity.

2.3.3 ASSESSMENT OF POTENTIAL RISKS AND BENEFITS

RECRUITMENT AND CONSENT

All personnel involved in the design and conduct of the research involving human participants will receive the required education on the protection of human research participants prior to the start of this project. Procedures to recruit participants for the protocol and obtain their informed consent or assent are conducted and supervised by the PI. Trained study staff will discuss the study, including the risks and benefits of participation, with potential participants and relevant members of their treatment team as needed to provide informed consent for interested individuals. Informed consent will be obtained from all participants before any study procedures are initiated. The consent form, which incorporates HIPAA authorization, contains a description of the purpose and procedures, risks, procedures to minimize them, and possible benefits. Participants will be assured that participation in the study in completely voluntary, and that they are free to withdraw consent at any time and discontinue participation without prejudice to their current or future

medical care. The objectives of the project, all of the requirements for participation, and any possible discomforts and risks will be clearly explained to the participants orally and in writing. All participants must sign a consent document (e-consent or paper), indicating their consent, approved by the Washington University School of Medicine Institutional Review Board, before they can participate in the study.

PREVENTING BREACH OF CONFIDENTIALITY

Electronic records (computer files, electronic databases, etc.): The risks of breaching confidentiality will be strictly limited by the use of locked and restricted access to data, as well as the use of participant ID numbers rather than names in the survey database. All other data will be collected/entered in the secured, centralized REDCap database.

Paper/hard copy records (hard copy surveys, questionnaires, consent, etc.): The majority of data for this study will be collected and maintained electronically. For participants without access to internet, we will collect consent and data using paper documents. The risks of breaching confidentiality for paper documents will be strictly limited by the use of locked and restricted access to data, as well as the use of participant ID numbers rather than names whenever possible. Medical records containing PHI and research records are stored using the two-lock rule.

Data and Safety Monitoring Plan:

Serious adverse events (SAE) and adverse evets will be monitored continuously throughout the study and will be reported to the DSMB and IRB according to pre-specified requirements. SAE/AE rates and interim study results will be reviewed and discussed by the DSMB at the DSMB meetings.

3 OBJECTIVES AND ENDPOINTS

OBJECTIVES	ENDPOINTS	JUSTIFICATION FOR ENDPOINTS
Primary		
**(a)** Participants randomized to fluvoxamine will have a lower rate of clinical deterioration, compared to their respective placebos. **(b)** Participants with baseline O_2 saturation ≤96% will have a larger treatment effect size with fluvoxamine (i.e., larger fluvoxamine-placebo difference in rate of clinical deterioration) than those with O_2 saturation ≥97%.	**The primary outcome will be clinical deterioration**, defined as both of the following: (1) presence of dyspnea (SOB rating of 4+ on the twice daily 0-10 scale, and/or hospitalization for shortness of breath or pneumonia, plus (2) decrease in O_2 saturation (<92% on room air via the twice-daily at-home self-monitoring or via monitoring at a hospital/ER/urgent care center, and/or supplemental oxygen requirement to keep O_2 saturation ≥92%).<br><br>Two blinded research staff will adjudicate all potential cases of clinical deterioration to confirm these criteria are (or are not) met.	The goal of the study is to test prevention of clinical deterioration. We chose a clinical outcome including a laboratory-confirmed (ie SpO2) endpoint, consistent with World Health Organization clinical trial recommendations (https://www.who.int/blueprint/15-01-2020-nfr-bp-wg-clinical-trials-ncov.pdf?ua=1). In our previous trial (STOP COVID), 6 participants met this endpoint; 5/6 went to the hospital and 4/6 were hospitalized for an average of 10 days, demonstrating the clinical relevance of this endpoint. Although we saw no mortality and only 1 ventilator, mortality from COVID-19 is largely

OBJECTIVES	ENDPOINTS	JUSTIFICATION FOR ENDPOINTS
		due to a generalized inflammatory response with a deterioration in the respiratory status being the largest contributor to death. A focus on clinical deterioration from a respiratory standpoint, commonly observed in COVID-19 patients early on prior to ventilator need, would be in line with WHO's scale and would be consistent with an early deterioration observed in the clinical course of COVID-19 as described in the symptomatology literature<br><br>Regarding baseline O_2 saturation potential moderator of treatment effect on outcome, this is based on observations made in our preliminary study: all 6 cases of clinical deterioration (in the placebo group) were among those with baseline O_2 saturation 96% or below.
Secondary		
Participants randomized to fluvoxamine will have higher post-COVID functional status at day 15 and day 90, compared to placebo.	**Secondary outcome will be post-COVID functioning** at 15 days and at 90 days using the PROMIS Global Health Scale. This secondary outcome reflects the increasing concern regarding persistence of COVID-19 illness and functional impairment in a substantial percentage of individuals with initially "mild" symptoms. We will also explore persistent symptoms of COVID-19 at these timepoints.	It is increasingly clear that COVID-19 is a persistent illness for some. Measuring this persistence is challenging because the symptoms are highly heterogeneous and temporally dynamic. An alternative is to use a validated measure of functional status to assess whether fluvoxamine results in an increased level of function and quality of life. The 10-item PROMIS Global Health Scale is a validated measure of function and quality of life.
Tertiary/Exploratory		
We will also explore symptoms of COVID-19 at approximately day 15 and day 90.	We will ask each participant about symptoms of COVID-19 on a 0-10 scale for greatest severity ever of the symptom, and a 1-10 scale for current severity of the symptom.	The long-term course of symptoms of COVID-19 is still poorly understood. These data will provide additional information about COVID-19 symptoms and whether their persistence is influenced by the study medications.

4 STUDY DESIGN

4.1 OVERALL DESIGN

Summary: We propose a fully-remote US study: we will screen, consent, deliver fluvoxamine/placebo and other supplies to (self-quarantined) patients, including thermometers, blood pressure cuffs, and O_2 saturation monitors. We will collect data via participants' phones/computers and via medical records. We will carry out national recruitment, similar to the University of Minnesota COVID-19 hydroxychloroquine trials carried out in early 2020.

Hypotheses:

Primary:

Participants randomized to fluvoxamine will have a lower rate of clinical deterioration, compared to placebo.

Secondary:

Participants randomized to fluvoxamine will have higher post-COVID functional status at day 15 and day 90, compared to placebo.

Participants with baseline O_2 saturation ≤96% will have a larger treatment effect size (i.e., larger fluvoxamine-placebo difference in rate of clinical deterioration) than those with O_2 saturation ≥97%.

Phase: 3

Design: randomized, placebo-controlled, double-blinded, parallel design, nationally-recruiting, fully-remote, multi-site.

Methods to minimize bias: placebo control, blinding, randomization.

Interim analyses planned: none.

Stratification planned: sex, age.

Study team: The complementary expertise of our group will assure the success of this clinical trial, as already evidenced by the successful conduct of the preliminary trial supporting this application.

Eric Lenze, M.D., is a clinical trialist and geriatric psychiatrist. He has successfully conducted numerous clinical trials, including large-scale trials. He and his research team led the STOP COVID trial that randomized 152 individuals to fluvoxamine or placebo for early treatment of COVID-19 and demonstrated fluvoxamine's efficacy at preventing clinical deterioration. He will lead this clinical trial along with his study team, overseeing all aspects of the research.

Angela Reiersen, M.D., M.P.E., is a child psychiatrist and epidemiologist with experience conducting studies of comorbidities, longitudinal course, and genetics (including pharmacogenetics) of various childhood-onset psychiatric disorders and a rare genetic disorder of endoplasmic reticulum (ER) stress (Wolfram Syndrome). She developed the hypothesis regarding fluvoxamine's potential anti-inflammatory benefits in the treatment of COVID-19. Dr. Reiersen hypothesized the potential anti-inflammatory effect may occur through fluvoxamine's known actions on the S1R-Inositol-requireing enzyme 1α (IRE1) pathway, which is a component of the ER stress response. She initially became interested in S1R ligands years ago due to observed differences in anxiety/depression treatment response to SSRIs with varying S1R actions, which she saw among study participants with Wolfram Syndrome. Along with Dr. Lenze, she developed and oversaw the STOP COVID trial. Her role in the proposed trial will include study design (with emphasis on development of appropriate symptom questionnaires and other outcome measures), supervision of study staff, interpretation of findings based on knowledge of drug mechanisms, supervision of data analysis, and publication of results.

Caline Mattar, M.D., is an infectious disease physician and global health researcher. She helped design and oversee the STOP COVID trial. She will continue to advise the conduct of this new study and will help interpret, publish, and disseminate the results.

William Powderly, M.D., is an infectious disease physician. He is the Director of the Institute for Public Health at Washington University in St. Louis. He also directs the Institute for Clinical and Translational Sciences (ICTS) and is co-director of the Division of Infectious Diseases at the Washington University School of Medicine. He is an expert on the immune pathologies present in severe COVID-19. He will provide advice on the conduct of this study, and will help interpret, publish, and disseminate the results.

Phil Miller, biostatistician, has directed data management and analysis for numerous clinical trials, including many large-scale efforts, and has worked with Dr. Lenze for the past 5 years on several successful clinical trials, including STOP COVID.

Michael Avidan, M.D., is a critical care and anesthesiology specialist. He has successfully conducted several large pragmatic clinical trials in critical care populations, including the use of intra-operative ketamine to prevent post-operative delirium. He will provide advice on the conduct of this study, and will help interpret, publish, and disseminate the results.

John W. Newcomer M.D., is a psychiatrist and the President and CEO of South Florida Behavioral Health Network in Miami, Florida. He has previously a professor in psychiatry at Washington University in St. Louis. He will act as a consultant on this project.

Mike Rich, M.D., is a cardiologist at Washington University. He is known for his work on clinical trials, including studies of antidepressant medication in medically ill populations. He is also the PI of a new study on long-term post-COVID-19 functioning.

Jeffrey Rado, M.D., is a psychiatrist and internist at Northwestern University. He is a clinical trialist who will lead the study at the Northwestern site.

Rachel Hess, M.D., is an internist and PI of the CTSA at University of Utah. She is an experienced clinical trialist who has carried out COVID-19 trials.

Adam Spivak, M.D., is an infectious disease specialist at University of Utah. He and Dr. Hess will lead the University of Utah site for this trial.

Joshua Schiffer, M.D., is an infectious disease specialist at Fred Hutchinson Cancer Research Center in Seattle, Washington. He is an experienced COVID-19 clinical trialist.

Rachel Bender Ignacio, M.D., is an infectious disease specialist at Fred Hutchinson Cancer Research Center in Seattle, Washington. She is an experienced COVID-19 clinical trialist. She and Dr. Schiffer will lead the Fred Hutch site for this trial.

Internal consultant Charles Zorumski, M.D., is a neuroscientist and is chair of Psychiatry at Washington University. He has been a member of the STOP COVID team and has provided scientific direction and will continue to do so.

Consultant David Boulware, M.D., is an infectious disease specialist. He has successfully conducted fully-remote clinical trials with national (and Canada) recruitment, including the hydroxychloroquine early treatment trial which randomized 491 patients with early COVID-19 in approximately 6 weeks. Dr. Boulware and his team has been advising the investigators on the development of this new trial and will continue to do so, to ensure successful recruitment, screening and remote assessment and treatment of a large national sample.

Consultant Matthew Pullen, M.D., is an infectious disease specialist. He worked with Dr. Boulware as a key team member in the hydroxychloroquine study. He will work with this team to ensure successful recruitment, screening and remote assessment and treatment of a large national sample.

4.2 SCIENTIFIC RATIONALE FOR STUDY DESIGN

Rationale for study design: placebo control provides the highest level of rigor for efficacy and safety analyses. A placebo control is both necessary and safe/ethical because of the lack of a widely-available standard of care treatment for early COVID-19 illness.

4.3 JUSTIFICATION FOR DOSE

Fluvoxamine: Participants receive a day zero dose of 50mg fluvoxamine (or matching placebo) in the evening immediately after the baseline assessment and confirmation of eligibility, and 100mg twice daily as tolerated, on days 1-15. This dose range was determined based on fluvoxamine's binding affinity for the S1R. After 15 days, this study drug is stopped; there is no need to taper fluvoxamine (i.e., to prevent SSRI withdrawal) after only a brief course.

The dosing target of 200mg (100mg twice daily) in the proposed study is also justified by experience from the STOP COVID trial. That preliminary study used a target of 100mg three times daily based on the FDA maximum dose of 300mg/day, and based on a review of the S1R agonist activity at various doses of fluvoxamine (*https://pubmed.ncbi.nlm.nih.gov/17662961/*). However, in STOP COVID almost all participants (86/90; 96%) in the fluvoxamine group achieved a dose of 200mg/day, but only 50% increased up to 300mg/day and of those that did, this was only after 5-6 days' treatment, which may have already been outside the period of greatest risk for clinical deterioration. In other words, it did not appear necessary to reach 300mg/d. We also reviewed fluvoxamine's pharmacokinetics and binding affinity at the S1R with several experts in pharmacology, who agreed that a maximum of 200mg/d was sufficient for adequate binding of the S1R.

4.4 END OF STUDY DEFINITION

The end of the study is day 90, when the participant has completed the day 90 survey; or when the participant has withdrawn consent or was withdrawn by the PI.

5 STUDY POPULATION

5.1 INCLUSION CRITERIA

Inclusion criteria:

1) Men and woman age 18 and older;

2) Not currently hospitalized

3) Proven SARS-CoV-2 positive (per lab or physician report).

4) Currently symptomatic with one or more of the following symptoms: fever, cough, myalgia, mild dyspnea, chest pain, diarrhea, nausea, vomiting, anosmia (inability to smell), ageusia (inability to taste), sore throat, nasal congestion.

5) Able to provide informed consent.

6) Upon initial screening, participant reports one of the following risk factors for clinical deterioration: age≥40, racial/ethnic group African-American, Hispanic, or Native American (including more than one race), or 1+ of the following medical conditions which increase risk for developing moderate-severe COVID illness: obesity, hypertension,

diabetes, heart disease (coronary artery disease, history of myocardial infarction, or heart failure), lung disease (eg asthma, COPD), immune disorder (eg rheumatoid arthritis, lupus).

5.2 EXCLUSION CRITERIA

Note: exclusions are same as in previous STOP COVID trial, but with additional exclusions for Coumadin and several other medications, based on recommendations of DSMB and co-investigators.

1) Illness severe enough to require hospitalization or already meeting study's primary endpoint for clinical worsening (eg current O_2 saturation <92% on room air, current use of supplemental oxygen to maintain O_2 saturation ≥92%).

2) Unstable medical comorbidities (eg decompensated cirrhosis), per patient report and/or medical records.

3) Immunocompromised (solid organ transplant, BMT, AIDS, on immunological biologics and/or high dose steroids (>20mg prednisone per day)

4) Already enrolled in another COVID 19 medication trial (not including vaccination or prophylaxis trials)

5) Unable to provide informed consent

6) Unable to perform the study procedures

7) Taking donepezil (rationale: donepezil is a S1R agonist), or sertraline (rationale: sertraline is a strong sigma-1 antagonist).

8) Taking warfarin-also known as Coumadin (rationale: increased risk of bleeding), phenytoin (rationale: fluvoxamine inhibits its metabolism), clopidogrel (rationale: fluvoxamine inhibits its metabolism from pro-drug to active drug which raises risk of cardiovascular events), and St John's wort (rationale: fluvoxamine + St John's wort are considered contraindicated because of the risk of serotonin syndrome)

9) Taking SSRIs, SNRIs, or tricyclic antidepressants, unless these are at a low dose such that a study investigator concludes that a clinically significant interaction with fluvoxamine (ie either serotonin syndrome or TCA overdose) is unlikely (examples: participant takes escitalopram but only at 5-10mg daily; that dose plus 200mg fluvoxamine would be insufficient to cause serotonin syndrome; or, participant takes amitriptyline but only at 25mg nightly; even if fluvoxamine inhibits its metabolism, it would be an insufficient dose to cause QTc prolongation or problematic side effects).

10) Individuals who report they have bipolar disorder or are taking medication for bipolar disorder (lithium, valproate, high-dose antipsychotic), unless the investigator concludes that the risk for mania is unlikely (ie it is doubtful that the patient actually has bipolar disorder).

11) Individuals who take alprazolam or diazepam and are unwilling to cut the medication by 25% (rationale: fluvoxamine modestly inhibits the metabolism of these drugs).

12) Participants taking theophylline, tizanidine, clozapine, or olanzapine (drugs with a narrow therapeutic index that are primarily metabolized by CYP 1A2, which is inhibited by fluvoxamine) will be reviewed with a study investigator and excluded unless the investigator concludes that the risk to the participant is low (this would be unlikely; example: participant takes tizanidine only as needed and is willing to avoid it for the 15 days of the study).

13) Received vaccine for COVID-19. Rationale: COVID-19 vaccines may prevent serious illness. Note that participants in vaccine trials are eligible, unless they know that they received the active vaccine.

5.3 SCREEN FAILURES

Screen failures are defined as participants who consent to participate in the clinical trial and subsequently randomly assigned to the study intervention but do not meet inclusion criteria prior to taking their first dose of study medication. Individuals who do not meet the criteria for participation in this trial (screen failure) will be rescreened if they subsequently meet inclusion criteria. The same subject number will be used.

5.4 STRATEGIES FOR RECRUITMENT AND RETENTION

This proposed remote clinical study will be conducted both locally through Washington University in St Louis, and nationally. The catchment reflects a diversity of settings including urban and rural settings, creating a diverse sample.

Study patients will be identified via a number of strategies, chiefly those shown effective in the fully-remote hydroxychloroquine studies: advertisements, media, and social media. As well, we will use strategies that were successful in the STOP COVID trial: using EHR, self-referral or through referrals from individual physicians. The research team, using interview with the potential participant and medical records review or discussion with the patient's physician, will then screen for eligibility as per the inclusion/exclusion criteria below.

Pre-screening and consent are conducted via REDCap surveys and telephone. No in person visits are required.

6 STUDY INTERVENTION

6.1 STUDY INTERVENTION(S) ADMINISTRATION

6.1.1 STUDY INTERVENTION DESCRIPTION

Participants are randomized to approximately 15 days of fluvoxamine or placebo. Study medications are matching oral capsules (taken twice daily). The study medication is stopped after approximately 15 days. The observation period for measuring the primary endpoint (clinical deterioration) is days 1-15 (beginning as soon as the first dose of the study medication and ending on day 15).

Sample size: We propose to enroll approximately n=1,100 participants, in order to have 880 in the modified intention to treat sample or analyzed sample. This sample will include all enrolled participants who were confirmed eligible at the baseline assessment and reporting taking at least one dose of study medication. A modified intention-to-treat strategy is required because participants are shipped supplies and study medication (whereupon they are randomized) prior to their measuring oxygen saturation to confirm study eligibility. In the preliminary study (STOP COVID), approximately 20% of individuals who received study supplies were not in the intention to treat sample, mainly because study eligibility could not be confirmed.

Randomization: Patients are randomized 1:1 to fluvoxamine or matching placebo capsules. A study statistician will generate randomization schedules stratified by age and sex. Treatments will be randomly allocated using alternating blocks of sizes 2 and 4. Randomization allocation is conducted via REDCap which displays randomization assignment to research team members who prepare study materials including drug or placebo but otherwise have no contact with

participants. All individuals who are in contact with participants are blinded, including outcome assessors and investigators, as are participants themselves.

6.1.2 DOSING AND ADMINISTRATION

Participants receive a day zero dose of 50mg fluvoxamine (or matching placebo) in the evening immediately after the baseline assessment and confirmation of eligibility (as is feasible; sometimes this dose is not taken until the next day), and fluvoxamine 100mg (or placebo) twice daily as tolerated, on days 1-15. Fluvoxamine will be self-administered.

6.2 MEASURES TO MINIMIZE BIAS: RANDOMIZATION AND BLINDING

Participants and all study staff (except for the study statistician running the randomization, and the study staff putting study medication into the package to deliver to participant) are blinded. This blinding is kept throughout the study until analyses of the primary endpoint are preliminarily conducted.

Blocked randomization will also reduce the chance of bias. Another strategy to reduce bias is that the primary endpoint, clinical deterioration, includes an objective component, oxygen saturation, which may be less prone to an expectancy effect (vs. self-reported measures). Blinded study staff will not have access to randomization assignment for participants. Study investigators will also be blinded.

Treatment guess: Participants will make a treatment guess at the end of the 15-day double-blind RCT phase, as is standard for double-blinded trials to test the adequacy of the blind.

6.3 STUDY INTERVENTION COMPLIANCE

Participant data, including IP compliance, will be collected via REDCap daily surveys. Each day the survey will ask the participant how many fluvoxamine (vs placebo) capsules they took.

7 STUDY INTERVENTION DISCONTINUATION AND PARTICIPANT DISCONTINUATION/WITHDRAWAL

7.1 DISCONTINUATION OF STUDY INTERVENTION

Management of participants who clinically deteriorate and/or are hospitalized or visit an emergency room or urgent care: We will make every effort to continue the study intervention throughout the 15-day period if patients are not hospitalized, or if they are hospitalized but do not meet criteria for clinical deterioration. However, we will not exclude hospitalized participants from other physician-directed treatments (e.g., remdesivir, dexamethasone, convalescent plasma). For patients who are hospitalized and show deterioration, such patients will discontinue fluvoxamine/placebo, with the rationale that this study's main goal is test whether fluvoxamine prevents deterioration, and in such cases, the participant will have met this study's primary endpoint (i.e., the prevention has "failed"). They would be eligible for any studies of treatments for serious COVID. We will continue to collect data including day-15 and day-90 data under the intention-to-treat principle, as well as the rationale that fluvoxamine may improve longer-term outcomes even in cases that it fails to prevent hospitalization.56

Discontinuation from fluvoxamine or placebo does not mean discontinuation from the study, and remaining study procedures should be completed as indicated by the study protocol whenever possible. If a clinically significant finding is identified (including, but not limited to changes from baseline) after enrollment, the investigator or qualified designee will determine if any change in participant management is needed. Any new clinically relevant finding will be reported as an adverse event (AE).

The data that we will attempt to collect at the time of study intervention discontinuation will include the following:

- Vital signs, including O2 saturation, pulse, blood pressure and temperature.
- Hospitalization and supplemental oxygen status.

7.2 PARTICIPANT DISCONTINUATION/WITHDRAWAL FROM THE STUDY

Participants are free to withdraw from participation in the study at any time upon request.

The PI may discontinue or withdraw a participant from the study for the following reasons:

- Participant is not contributing data and/or appears to be lost to follow-up.
- If any clinical adverse event (AE), laboratory abnormality, or other medical condition or situation occurs such that continued participation in the study would not be in the best interest of the participant or interferes with the integrity of the study data.
- Disease progression which requires discontinuation of the study intervention
- If the participant meets an exclusion criterion (either newly developed or not previously recognized) that precludes further study participation

8 STUDY ASSESSMENTS AND PROCEDURES

8.1 EFFICACY ASSESSMENTS

Outcome Assessments: The primary endpoint will be clinical deterioration of COVID-19 illness. The secondary endpoint will be day 15 and day 90 functioning.

Clinical deterioration is defined meeting both of the following: (1) presence of dyspnea and/or hospitalization for shortness of breath or pneumonia, plus (2) decrease in O_2 saturation (<92%) on room air and/or supplemental oxygen requirement in order to keep O_2 saturation ≥92%.

The primary method of assessment for these outcomes is participants' reports on twice-daily surveys throughout the 15-day randomized period, corroborated by research staff (e.g., calling participants when low oxygen saturation was reported). For participants who stop answering the surveys prior to day 15, as well as those who appear to be meeting the primary endpoint, we will use, as is feasible, medical records and subsequent calls to these participants and their family to determine whether they sought medical care for shortness of breath or hypoxia. We will also confirm medical care needed (e.g., supplemental oxygen, length of stay, ventilator support) as is feasible.

We will collect sufficient data on all participants to also measure the clinical deterioration on the World Health Administration COVID-19 clinical trial outcome scale: 0-1: ambulatory (no clinical deterioration during RCT phase), 2: limitation of activities but no hospitalization; 3: hospitalization but no O_2 needed; 4: hospitalization, O_2 needed; 5: non-invasive ventilation or high-flow oxygen; 6: ventilator needed; 7: ventilator plus organ support needed; 8: death. Scale can be found on p6 of: *https://www.who.int/blueprint/priority-diseases/key-action/COVID-19_Treatment_Trial_Design_Master_Protocol_synopsis_Final_18022020.pdf*. Since ordinal scales have proved useful in studies of hospitalized patients with respiratory illnesses, this measure will be particularly helpful as an outcome measure for the subset of study participants who require hospitalization. This cross-walk with the WHO scale is also useful given that our primary outcome is a study-specific scale.

The secondary endpoint will be functioning at day 15 and day 90, measured by the 10-item PROMIS Global Health Scale ("Global-10"). This scale's items assess general domains of health and functioning including overall physical health, mental health, social health, pain, fatigue, and overall perceived quality of life. The 10 questions of the Global-10 have

largely been adapted from older measures such as the *SF-36* and *EQ-5D*, with modifications that resulted in greater sensitivity and precision than the questions as originally worded.

We will also measure persistence of COVID-19 symptoms as an exploratory measure, asking about all symptoms that have been reported in the literature to date on a 0-10 scale (0= absent; 10=severe).

Data collection and participant interaction specifics: All interactions with participants will be fully-remote via phone/text/email/videoconference. Data collection is done by twice-daily REDCap surveys sent to patients via email, with telephone-based data collection as a back-up to ensure that individuals without internet access are able to participate. The surveys record oxygen saturation, vital signs, medication adherence, and COVID-19 symptoms. Fixed race and ethnicity categories are collected by interviewers as part of the demographic information to characterize the sample. The surveys measure dyspnea (shortness of breath) using a continuous 0-10 scale (0= symptom not present, 10= symptom is very severe) via the technique of Ecological Momentary Assessment (i.e., "how bad is your symptom right now?"). Phone contact is attempted daily during the first three days of the trial to address participants' questions and concerns such as medication-related issues, and throughout the trial to remind them to complete assessments as necessary. Additional phone calls are conducted on a case-by-case basis when participants' survey data indicate out of range values (e.g., low O_2 saturation) and to recheck and confirm these as necessary. Also, for participants who appear to be having worsening COVID-19 illness, study staff recommend that they seek medical attention from their primary care provider or via a local emergency room.

8.2 SAFETY AND OTHER ASSESSMENTS

The first assessment of safety will be conducted at screening. Research staff will, via telephone, determine whether participants meet eligibility criteria and what are their medical conditions and medications. This information will be provided to the study physician investigator to concur with enrollment, prior to starting any study medication. For example, if participants are taking an SSRI or a medication of concern for interaction with fluvoxamine (e.g., olanzapine), this will need to be reviewed by the physician to determine whether the participant could safely participate in the study.

The second assessment of safety is conducted when study materials (including pulse oximeter, blood pressure cuff, and thermometer). Participants will report on their O2 saturation, vital signs, and willingness to proceed, via telephone with study staff, prior to starting study medication.

The third assessment of safety is throughout days 1-15 of the study. Participants will report on their O2 saturation, vital signs, symptoms, and any adverse events, via the twice-daily survey, with telephone corroboration with study staff as necessary.

All participants will, as is feasible, have a phone visit with study staff daily on days 1-3. Additional phone calls will be as needed (e.g., to confirm out of bounds values on O2 saturation or vitals, and to advise participants whether to call their provider or go to an emergency room).

Medical records, if available, will be used to supplement each of these assessments.

9 STATISTICAL CONSIDERATIONS

9.1 STATISTICAL HYPOTHESES

A formal Statistical Analysis Plan (SAP) will be completed prior to database lock and unblinding of the study data. It will include any additional details regarding the strategies described in this section.

The primary hypotheses (superiority hypotheses) are:

Participants randomized to fluvoxamine will have a lower rate of clinical deterioration, compared to placebo. (Null: participants randomized to fluvoxamine will not have a lower rate of clinical deterioration, compared to placebo)

- Primary Efficacy Endpoint:

Participants will meet the primary endpoint of clinical deterioration at the point that they meet both of the following: (1) presence of dyspnea and/or hospitalization for shortness of breath or pneumonia, plus (2) decrease in O_2 saturation (<92% on room air and/or supplemental oxygen requirement to keep O_2 saturation ≥92%). This will be measured during study days 1-15. The primary source of information to ascertain this endpoint is the twice-daily survey which is carried out on study days 1-15; this survey includes participant self-report of their shortness of breath (dyspnea) rating on a 0-10 scale (where a score of 4 or greater, indicating at least moderate dyspnea, meeting the first part of this endpoint) and of their pulse oximeter reading (where a reading of <92% meets the second part of this endpoint). Study staff will confirm the pulse oximeter readings with participants via phone when possible, asking them to re-check, and will call participants to collect these data via telephone if surveys have been skipped. Other data sources for this endpoint include participant and/or family report, or medical records data, regarding medical care received, during study days 1-15, in a hospital/ER/urgent care center.

The secondary hypotheses (also superiority hypotheses) are:

Participants randomized to fluvoxamine will have higher post-COVID functional status at day 15 and day 90, compared to placebo.

- Secondary Efficacy Endpoint(s):

Secondary outcome will be post-COVID functioning measured at 15 days and at 90 days post-randomization using the PROMIS 10-item Global Health Scale. The source of this data will be a survey sent to participants on these days, with data collection via telephone if the survey is skipped.

9.2 SAMPLE SIZE DETERMINATION

Software used for power calculations was power.prop.test in R.

With a sample size of 880 in the modified intention to treat sample, we will have 80% power to detect a treatment effect of 35% reduction (20% in placebo vs. 13% in fluvoxamine). Two-tailed alpha=0.05 for this power analysis, as time to clinical deterioration is the primary analysis and endpoint.

The basis for the assumption of 20% meeting the clinical deterioration endpoint (in the placebo group) is the enrichment strategy for this trial, in which participants must indicate, on screening, at least one risk factor for clinical deterioration (e.g., age, medical condition).

The basis for the assumption of 35% reduction: (1) 35% would be a clinically meaningful relative risk reduction in a high-risk group; (2) a 35% relative risk reduction is what has been reported with corticosteroids such as dexamethasone when given in more serious COVID-19.

To achieve n=880 in the modified intention to treat sample, we anticipate enrolling 1100 individuals, because we expect 20% dropout/ineligible from the time of shipping/couriering study supplies to the first dose of medication, as in the preliminary clinical trial. The main reason for dropout/ineligibility in the preliminary study was that we were unable to confirm study entry criteria, which included a baseline O_2 saturation of 92% or greater. This level of dropout/withdrawal after consent and shipping supplies is also consistent with the hydroxychloroquine early treatment trial conducted by University of Minnesota.

This sample size has suitable power for the secondary endpoint analysis (function at day 15 and day 90). For a simple t-test between two groups, n=400 each (allowing for dropout), we can detect a difference of 0.2 standard deviations (Fleiss' d) with a power of 80%; i.e., a small effect size.

The examination of persistent symptoms at day 15 and day 90 will be exploratory only; the analytic plan will depend on the structure of the data collected. Therefore no power analysis was conducted for this exploratory examination.

The moderator hypothesis is: Participants with baseline O_2 saturation ≤96% will have a larger treatment effect size (i.e., larger fluvoxamine-placebo difference in rate of clinical deterioration) than those with O_2 saturation ≥97%.

This moderator hypothesis is consistent with the strategy of prognostic enrichment, and our methodology and analytic strategy follows the FDA guidance on prognostic enrichment, found in *https://www.fda.gov/regulatory-information/search-fda-guidance-documents/enrichment-strategies-clinical-trials-support-approval-human-drugs-and-biological-products*.

The test for moderation is a test of the interaction between treatment and baseline O_2 saturation (dichotomized as ≤96% vs. ≥97%).

Power for this moderator hypothesis: We have at least 80% power at p=0.016 to find an effect size modulation such that in the low-risk group (baseline O_2 saturation ≥97%) there was a treatment odds ratio of 1 (e.g., equal proportions of deterioration in both fluvoxamine and placebo groups), while in the high-risk group (baseline O_2 saturation≤96%) there was a treatment odds ratio of 0.4 (i.e., 12% deterioration in the fluvoxamine group vs. 26% in the placebo group).

This is not a power calculation for the moderation per se, but is an example of a moderation effect which could be detected. The odds ratios and the standard error of the log odds ratio are based on the usual formulas, e.g.
OR = *x11*x22)/(x21*x12)
SE(log(OR)) = sqrt(1/x11 + 1/x22 + 1/x21 + 1/x12)
as is a simple Z test of the difference between the odds ratios and its P value.

9.3 STATISTICAL ANALYSES

.

9.3.1 GENERAL APPROACH

- For descriptive statistics, categorical data will be presented as n (percentage), and continuous data will be presented as means with standard deviations, or median with interquartile range.
- For all inferential tests, p-value will be 0.05 with 95% confidence intervals reported, all two-tailed.
- Any covariates will be specified at the time of conducting analyses, but prior to unblinding.
- The primary analysis requires no checks for normality; the secondary endpoint analysis will require a check of normality and corrective procedures will be applied (e.g., transformation or nonparametric tests).
- We will use SAS 9.4 for analyses.

9.3.2 ANALYSIS OF THE PRIMARY EFFICACY ENDPOINT(S)

The primary analysis is survival analysis of the primary outcome (clinical deterioration). This analysis treats participants as censored on the day that they meet the primary outcome, or on the last day that they fill out an outcome

assessment. It is superior to an exact test of proportions because of the ability to handle missing data (due to dropout or meeting endpoint).

Findings will be reported as the rates of clinical deterioration in groups (i.e., fluvoxamine vs. placebo), the absolute difference in the endpoint, the logrank chi square, and the respective p-value.

No imputation for missing data will be used. However, for all participants who do not complete surveys through day 15, we will use follow-up phone calls and medical records to ascertain whether they met the endpoint (e.g., were hospitalized and received supplemental oxygen).

9.3.3 ANALYSIS OF THE SECONDARY ENDPOINT(S)

Because of the potential for type 1 error due to multiple comparisons, findings for analyses of secondary endpoint (global function at day 15 and 90) should be interpreted as exploratory. The analyses will proceed regardless of findings of primary endpoint.

The measurement is the summary of the 10-item scale which is expected to have a normal distribution. It will be examined separately at days 15 and 90 rather than as a repeated measure. The scale is not measured at baseline, so the comparison is a t-test or ANCOVA (if covariates are used) between intervention and placebo at these time points (day 15, day 90).

No imputation for missing data will be used.

10 SUPPORTING DOCUMENTATION AND OPERATIONAL CONSIDERATIONS

10.1 REGULATORY, ETHICAL, AND STUDY OVERSIGHT CONSIDERATIONS

10.1.1 FUTURE USE OF STORED SPECIMENS AND DATA

Data collected for this study will be analyzed and stored at Washington University. After the study is completed, the de-identified, archived data will be stored at Washington University for use by other researchers including those outside of the study. Permission to transmit data to other researchers will be included in the informed consent.

10.1.2 SAFETY OVERSIGHT

We will appoint DSMB members who are not involved in the study (i.e., as investigators). The board will include individuals with expertise important to this trial who will be responsible for overseeing the safety of participants and overseeing that the trial's conduct.

The DSMB will meet, at intervals determined by the DSMB, during the study (e.g., prior to start of recruitment, various times during recruitment phase, and as needed). They will review study processes such as screening rate, enrollment rate, follow-up rate, dropout rate, demographics of enrolled sample, baseline clinical data, and participant safety. The primary safety measure will be AE reports.

10.1.3 QUALITY ASSURANCE AND QUALITY CONTROL

Each clinical site will perform internal quality management of study conduct, data collection, documentation and completion.

Quality control (QC) procedures will be implemented beginning with the data entry system and data QC checks that will be run on the database will be generated. Any missing data or data anomalies will be communicated to the site(s) for clarification/resolution.

The investigational site will provide direct access to all trial related sites, source data/documents, and reports for the purpose of monitoring and auditing by the sponsor, and inspection by local and regulatory authorities.

11 REFERENCES

1. Wiersinga WJ, Rhodes A, Cheng AC, Peacock SJ, Prescott HC. Pathophysiology, Transmission, Diagnosis, and Treatment of Coronavirus Disease 2019 (COVID-19): A Review. *JAMA.* 2020;324(8):782-793.
2. Guan WJ, Ni ZY, Hu Y, et al. Clinical Characteristics of Coronavirus Disease 2019 in China. *N Engl J Med.* 2020;382(18):1708-1720.
3. Huang C, Wang Y, Li X, et al. Clinical features of patients infected with 2019 novel coronavirus in Wuhan, China. *Lancet.* 2020;395(10223):497-506.
4. Lescure FX, Bouadma L, Nguyen D, et al. Clinical and virological data of the first cases of COVID-19 in Europe: a case series. *Lancet Infect Dis.* 2020;20(6):697-706.
5. Mehta P, McAuley DF, Brown M, et al. COVID-19: consider cytokine storm syndromes and immunosuppression. *Lancet.* 2020;395(10229):1033-1034.
6. Prasad A, Prasad M. Single Virus Targeting Multiple Organs: What We Know and Where We Are Heading? *Front Med (Lausanne).* 2020;7:370.
7. Del Valle DM, Kim-Schulze S, Huang H-H, et al. An inflammatory cytokine signature predicts COVID-19 severity and survival. *Nat Med.* 2020;26(10):1636-1643.
8. Laing AG, Lorenc A, del Molino del Barrio I, et al. A dynamic COVID-19 immune signature includes associations with poor prognosis. *Nat Med.* 2020;26(10):1623-1635.
9. Rubin R. As Their Numbers Grow, COVID-19 “Long Haulers” Stump Experts. *JAMA.* 2020.
10. Rosen DA, Seki SM, Fernandez-Castaneda A, et al. Modulation of the sigma-1 receptor-IRE1 pathway is beneficial in preclinical models of inflammation and sepsis. *Sci Transl Med.* 2019;11(478).
11. Bourrie B, Bribes E, Derocq JM, Vidal H, Casellas P. Sigma receptor ligands: applications in inflammation and oncology. *Current opinion in investigational drugs (London, England : 2000).* 2004;5(11):1158-1163.
12. Lewis R, Li J, McCormick PJ, C LHH, Jeevaratnam K. Is the sigma-1 receptor a potential pharmacological target for cardiac pathologies? A systematic review. *International journal of cardiology Heart & vasculature.* 2020;26:100449.
13. Oxombre B, Lee-Chang C, Duhamel A, et al. High-affinity σ1 protein agonist reduces clinical and pathological signs of experimental autoimmune encephalomyelitis. *Br J Pharmacol.* 2015;172(7):1769-1782.
14. Hashimoto K. Activation of sigma-1 receptor chaperone in the treatment of neuropsychiatric diseases and its clinical implication. *J Pharmacol Sci.* 2015;127(1):6-9.
15. Cobos EJ, Entrena JM, Nieto FR, Cendan CM, Del Pozo E. Pharmacology and therapeutic potential of sigma(1) receptor ligands. *Curr Neuropharmacol.* 2008;6(4):344-366.
16. Hallifax D, Houston JB. Saturable Uptake of Lipophilic Amine Drugs into Isolated Hepatocytes: Mechanisms and Consequences for Quantitative Clearance Prediction. *Drug Metabolism and Disposition.* 2007;35(8):1325.
17. Deftereos SG, Giannopoulos G, Vrachatis DA, et al. Effect of Colchicine vs Standard Care on Cardiac and Inflammatory Biomarkers and Clinical Outcomes in Patients Hospitalized With Coronavirus

Disease 2019: The GRECCO-19 Randomized Clinical Trial. *JAMA Network Open.* 2020;3(6):e2013136-e2013136.
18. Group TWREAfC-TW. Association Between Administration of Systemic Corticosteroids and Mortality Among Critically Ill Patients With COVID-19: A Meta-analysis. *JAMA.* 2020.
19. Kox M, Waalders NJB, Kooistra EJ, Gerretsen J, Pickkers P. Cytokine Levels in Critically Ill Patients With COVID-19 and Other Conditions. *JAMA.* 2020.
20. Mofsen AM, Rodebaugh TL, Nicol GE, Depp CA, Miller JP, Lenze EJ. When All Else Fails, Listen to the Patient: A Viewpoint on the Use of Ecological Momentary Assessment in Clinical Trials. *JMIR Ment Health.* 2019;6(5):e11845.
21. Narita N, Hashimoto K, Tomitaka S, Minabe Y. Interactions of selective serotonin reuptake inhibitors with subtypes of sigma receptors in rat brain. *Eur J Pharmacol.* 1996;307(1):117-119.
22. Delprat B, Crouzier L, Su TP, Maurice T. At the Crossing of ER Stress and MAMs: A Key Role of Sigma-1 Receptor? *Adv Exp Med Biol.* 2020;1131:699-718.
23. Mori T, Hayashi T, Hayashi E, Su TP. Sigma-1 receptor chaperone at the ER-mitochondrion interface mediates the mitochondrion-ER-nucleus signaling for cellular survival. *PLoS One.* 2013;8(10):e76941.
24. Hoffmann M, Kleine-Weber H, Schroeder S, et al. SARS-CoV-2 Cell Entry Depends on ACE2 and TMPRSS2 and Is Blocked by a Clinically Proven Protease Inhibitor. *Cell.* 2020;181(2):271-280 e278.
25. Knoops K, Kikkert M, Worm SH, et al. SARS-coronavirus replication is supported by a reticulovesicular network of modified endoplasmic reticulum. *PLoS Biol.* 2008;6(9):e226.
26. Gassen NC, Papies J, Bajaj T, et al. Analysis of SARS-CoV-2-controlled autophagy reveals spermidine, MK-2206, and niclosamide as putative antiviral therapeutics. *bioRxiv.* 2020:2020.2004.2015.997254.
27. Laurent EMN, Sofianatos Y, Komarova A, et al. Global BioID-based SARS-CoV-2 proteins proximal interactome unveils novel ties between viral polypeptides and host factors involved in multiple COVID19-associated mechanisms. *bioRxiv.* 2020:2020.2008.2028.272955.
28. Gordon DE, Jang GM, Bouhaddou M, et al. A SARS-CoV-2 protein interaction map reveals targets for drug repurposing. *Nature.* 2020;583(7816):459-468.
29. Yang H, Shen H, Li J, Guo LW. SIGMAR1/Sigma-1 receptor ablation impairs autophagosome clearance. *Autophagy.* 2019;15(9):1539-1557.
30. Gorshkov K, Chen CZ, Bostwick R, et al. The SARS-CoV-2 cytopathic effect is blocked with autophagy modulators. *bioRxiv : the preprint server for biology.* 2020:2020.2005.2016.091520.
31. Homolak J, Kodvanj I. Widely available lysosome targeting agents should be considered as potential therapy for COVID-19. *Int J Antimicrob Agents.* 2020;56(2):106044.
32. Daniel WA, Wojcikowski J. Contribution of lysosomal trapping to the total tissue uptake of psychotropic drugs. *Pharmacol Toxicol.* 1997;80(2):62-68.
33. Fung TS, Liu DX. The ER stress sensor IRE1 and MAP kinase ERK modulate autophagy induction in cells infected with coronavirus infectious bronchitis virus. *Virology.* 2019;533:34-44.
34. Kornhuber J, Tripal P, Gulbins E, Muehlbacher M. Functional inhibitors of acid sphingomyelinase (FIASMAs). *Handb Exp Pharmacol.* 2013(215):169-186.
35. Breiden B, Sandhoff K. Emerging mechanisms of drug-induced phospholipidosis. *Biol Chem.* 2019;401(1):31-46.
36. Chan SW. The unfolded protein response in virus infections. *Front Microbiol.* 2014;5:518.
37. Jheng JR, Ho JY, Horng JT. ER stress, autophagy, and RNA viruses. *Front Microbiol.* 2014;5:388.
38. Nabirotchkin S, Peluffo AE, Bouaziz J, Cohen D. Focusing on the Unfolded Protein Response and Autophagy Related Pathways to Reposition Common Approved Drugs against COVID-19. *Preprints.* 2020.
39. Hosszu A, Antal Z, Lenart L, et al. sigma1-Receptor Agonism Protects against Renal Ischemia-Reperfusion Injury. *J Am Soc Nephrol.* 2017;28(1):152-165.
40. Tagashira H, Bhuiyan MS, Fukunaga K. Diverse regulation of IP3 and ryanodine receptors by pentazocine through sigma1-receptor in cardiomyocytes. *Am J Physiol Heart Circ Physiol.* 2013;305(8):H1201-1212.
41. Manne BK, Denorme F, Middleton EA, et al. Platelet gene expression and function in patients with COVID-19. *Blood.* 2020;136(11):1317-1329.

42. Schlienger RG, Meier CR. Effect of selective serotonin reuptake inhibitors on platelet activation: can they prevent acute myocardial infarction? *Am J Cardiovasc Drugs.* 2003;3(3):149-162.
43. van den Berg DF, Te Velde AA. Severe COVID-19: NLRP3 Inflammasome Dysregulated. *Front Immunol.* 2020;11:1580.
44. Ratajczak MZ, Kucia M. SARS-CoV-2 infection and overactivation of Nlrp3 inflammasome as a trigger of cytokine "storm" and risk factor for damage of hematopoietic stem cells. *Leukemia.* 2020;34(7):1726-1729.
45. Garcia JA, Volt H, Venegas C, et al. Disruption of the NF-kappaB/NLRP3 connection by melatonin requires retinoid-related orphan receptor-alpha and blocks the septic response in mice. *FASEB J.* 2015;29(9):3863-3875.
46. Volt H, Garcia JA, Doerrier C, et al. Same molecule but different expression: aging and sepsis trigger NLRP3 inflammasome activation, a target of melatonin. *J Pineal Res.* 2016;60(2):193-205.
47. Hartter S, Wang X, Weigmann H, et al. Differential effects of fluvoxamine and other antidepressants on the biotransformation of melatonin. *J Clin Psychopharmacol.* 2001;21(2):167-174.
48. Henry JA. Overdose and safety with fluvoxamine. *Int Clin Psychopharmacol.* 1991;6 Suppl 3:41-45; discussion 45-47.
49. Lenze EJ, Mattar C, Zorumski CF, et al. Fluvoxamine vs Placebo and Clinical Deterioration in Outpatients With Symptomatic COVID-19: A randomized clinical trial. *JAMA. Published online November 12, 2020.* doi:10.1001/jama.2020.22760.

FLUVOXAMINE FOR EARLY TREATMENT OF COVID-19: A FULLY-REMOTE, RANDOMIZED PLACEBO CONTROLLED TRIAL

Principal Investigator: Eric Lenze, MD and Angela Reiersen, MD

Sponsor: Washington University School of Medicine

Funded by: COVID-19 Early Treatment Fund

Version Number: v. 5

April 14, 2021

1 PROTOCOL SUMMARY

1.1 SYNOPSIS

Title: FLUVOXAMINE FOR EARLY TREATMENT OF COVID-19: A FULLY-REMOTE, RANDOMIZED PLACEBO CONTROLLED TRIAL

Study Description: This clinical trial will test a promising drug for early treatment of COVID-19 in 880 adults who are SARS-CoV-2 positive and recently infected. The trial will determine whether fluvoxamine (1) prevents clinical deterioration (defined as the combination of shortness of breath and hypoxia), and (2) improves short- and long-term functional outcome. Clinical deterioration is the primary endpoint. Fluvoxamine is an SSRI (FDA approved for OCD and Social Anxiety Disorder) that also activates the sigma-1 receptor (an immunomodulatory receptor). It prevented clinical deterioration in a preliminary RCT (called STOP COVID). Fluvoxamine (or placebo) will be given for approximately 15 days post-randomization, during which participants will self-report on shortness of breath and oxygen saturation via a twice-daily survey. At the end of approximately 15 days, fluvoxamine (or placebo) will be stopped. An additional survey at approximately day 15 and approximately day 90 will assess function.

Objectives:

The primary objective of this clinical trial is to test whether fluvoxamine prevents clinical deterioration in early COVID-19 illness.

The primary outcome will be clinical deterioration, defined as both of the following: (1) presence of dyspnea and/or hospitalization for shortness of breath or pneumonia, plus (2) decrease in O_2 saturation (<92% on room air and/or supplemental oxygen requirement to keep O_2 saturation ≥92%).

The hypotheses are:

Participants randomized to fluvoxamine will have a lower rate of clinical deterioration, compared to placebo.

A secondary objective of this clinical trial is to test whether fluvoxamine improves 15-day and 90-day functional outcome in early COVID-19 illness.

Secondary outcome will be post-COVID functioning at approximately 15 days and at approximately 90 days post-randomization using the PROMIS 10-item Global Health Scale. This secondary outcome reflects the increasing concern regarding persistence of COVID-19 illness and functional impairment in a substantial percentage of individuals with initially “mild” symptoms. We will also explore persistent symptoms of COVID-19 at these timepoints.

The hypotheses are:

Participants randomized to fluvoxamine will have higher post-COVID functional status at day 15 and day 90, compared to placebo.

Another secondary objective is to test whether baseline oxygen saturation moderates (modulates the drug-placebo difference) fluvoxamine's effect on clinical deterioration.

The hypothesis is:

Participants with baseline O_2 saturation ≤96% will have a larger treatment effect size (i.e., larger fluvoxamine-placebo difference in rate of clinical deterioration) than those with O_2 saturation ≥97%.

The basis for this hypothesis is an exploratory analysis of the preliminary RCT which found that all cases of clinical deterioration (26% of the placebo group) occurred in participants whose baseline oxygen saturation was 96% or less.

**Endpoints:**	Primary Endpoint: Clinical Deterioration<br>Secondary Endpoints: PROMIS Global Health Scale
**Study Population:**	Not currently hospitalized, participants age 30 and older who are SARS-CoV-2 positive and symptomatic with onset <7 days.
**Phase:**	Phase 3
**Description of Sites/Facilities Enrolling Participants:**	Washington University School of Medicine in St. Louis<br>Northwestern University<br>University of Utah<br>Fred Hutchinson Center Clinical Research Center<br>Icahn School of Medicine at Mount Sinai
**Description of Study Intervention:**	Fluvoxamine Maleate, 100mg twice daily x 15 days
**Study Duration:**	Up to 3 months
**Participant Duration:**	Up to 3 months

1.2 SCHEMA

Diagram 1: Randomized Clinical Trial

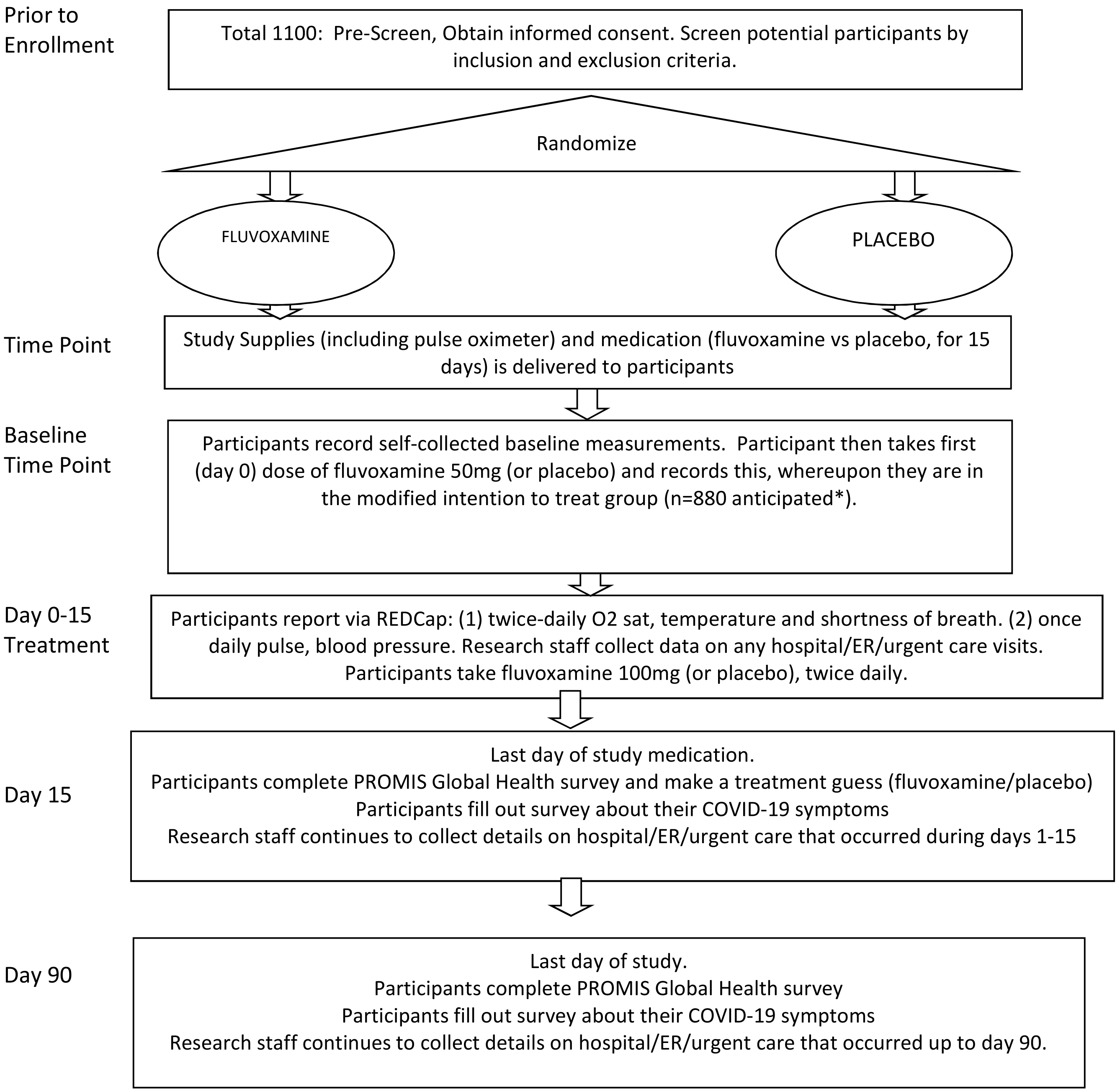

*Note this number (n=880) refers to the modified intent to treat sample: all participants for whom study eligibility is met at baseline and who take at least one dose of study medication. All data analyses will be conducted using this sample. The larger number n=1100 refers to the estimated number who will receive study medication (and therefore are considered randomized) in order to achieve n=880 in the modified intent to treat sample.

1.3 SCHEDULE OF ACTIVITIES (SOA)

**Procedures**	Screening Day -5 to 0	Enrollment/Baseline	Treatment days 0-15	Day 15 Treatment	Day 90 Post Treatment
Informed consent	X				
Demographics	X				
Medical history	X				
Randomization	X				
Administer study intervention		X			X
Twice-daily REDCap survey to assess oxygen saturation, self-reported dyspnea, vital signs, and treatment adherence	X--X				
Assessment of details of COVID-19 care received (e.g.. hospitalization, ER, or urgent care visit; length of stay; requirement for supplemental oxygen, ventilator, or other organ support; mortality)	X--X				X
Adverse event review and evaluation	X--X				X
Treatment guess (patient guesses whether they took fluvoxamine vs placebo)				X	

2 INTRODUCTION

2.1 STUDY RATIONALE

2.2 BACKGROUND

Coronavirus disease 2019 (COVID-19), caused by the novel severe acute respiratory syndrome coronavirus 2 (SARS-CoV-2), can cause serious illness including lung damage, hypoxia, and cardiac damage, which can lead to hospitalization, intensive care unit (ICU) admission, and death.[1]

Clinical deterioration often occurs during the second week of illness. Early investigations of COVID-19 found that serious illness leading to hospital admission occurred a median of 8-10 days after initially mild to moderate symptoms.[2-4] Evidence that many patients with COVID-19 develop lung damage from an excessive inflammatory response led to recommendations to repurpose immunomodulatory drugs to counter this hyperinflammation.[5,6] More recent evidence supports the assertion that the inflammatory reaction, including elevations in cytokines such as IL-6, predicts clinical deterioration.[7,8]

Many patients have debilitating persistent symptoms and loss of function, even three or more months after the initial stage of illness. The causes of this persistent illness are unclear and likely heterogeneous; they include persistence of the viral infection, immunological changes, and exacerbation of underlying medical conditions.[9] As a result, both anti-viral and immunomodulatory drugs could potentially prevent or reduce this post-COVID functional impairment.

Fluvoxamine, an SSRI, repurposed for early treatment of COVID-19

Fluvoxamine is unique among the SSRIs in that it strongly activates the sigma-1 receptor (S1R), and preclinical studies have suggested that by this mechanism fluvoxamine could prevent the immunopathology seen in serious COVID-19. In 2019, it was shown that fluvoxamine given early in treatment, reduced deterioration and mortality in two animal models of sepsis (bacterial peritonitis and lipopolysaccharide stimulation).[10] The same study demonstrated that fluvoxamine was anti-inflammatory in human cells, reducing IL-6 and other cytokines in lipopolysaccharide-induced inflammation. This study is part of an increasing body of research supporting the S1R in protection from deleterious consequences of inflammation.[11-13]

Fluvoxamine has one of the strongest S1R agonist effects of any existing medication.[14,15] It is also highly lipophilic and has rapid, substantial intracellular uptake.[16] Fluvoxamine also has many pragmatic advantages for repurposing, including ease of use, high safety margin, good tolerability, wide availability, and low cost. We determined that, if it could be shown that fluvoxamine had a beneficial effect in reducing clinical deterioration of COVID-19, the drug (and other S1R agonists) could be an important addition to the armamentarium vs. this pandemic.

Therefore, we conducted a double-blind, placebo-controlled RCT to test whether fluvoxamine, given as early treatment in individuals with mild COVID-19 illness, might prevent clinical deterioration. [49]

From April 10-Sept 19, 2020, we conducted the STOP COVID trial (ClinicalTrials.gov Identifier: NCT04342663), a preliminary single-site trial conducted in Missouri and Illinois, to test whether a course of fluvoxamine given early in COVID-19 illness could prevent clinical deterioration. Participants were recruited mainly from review of electronic medical records to identify individuals with a recent positive SARS-CoV-2 PCR test. Based on review of the medical record, potentially eligible individuals were contacted to confirm eligibility criteria and offer participation in the study. The study was fully-remote, with no in-person contact with participants.

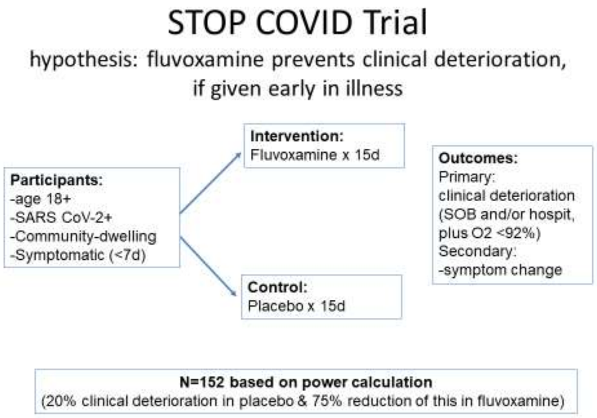

The STOP COVID participant flow diagram is shown along with participant baseline characteristics (*see Figure and Tables on next page*). Participants were randomized to receive fluvoxamine 100 mg vs. placebo up to three times daily. Study medication and monitoring equipment (fingertip pulse oximeter, blood pressure cuff, thermometer) were delivered directly to participant homes. Participants entered their own O_2 saturation, vital signs, and symptom rating data twice daily for 15 days online or via phone into REDCap surveys, which were monitored daily by study staff. Participants were contacted as needed to clarify symptom and vitals/ O_2 saturation (e.g., when participants reported an out-of-bounds value or one indicating hypoxia). This monitoring included study staff, under study physician supervision, advising participants to contact their physician or visit an emergency room when appropriate. The primary study endpoint was time to clinical worsening, which was defined as 1) dyspnea or hospitalization for shortness of breath or pneumonia and 2) O_2 saturation <92% or requirement for supplemental O_2 to keep saturation ≥92%.

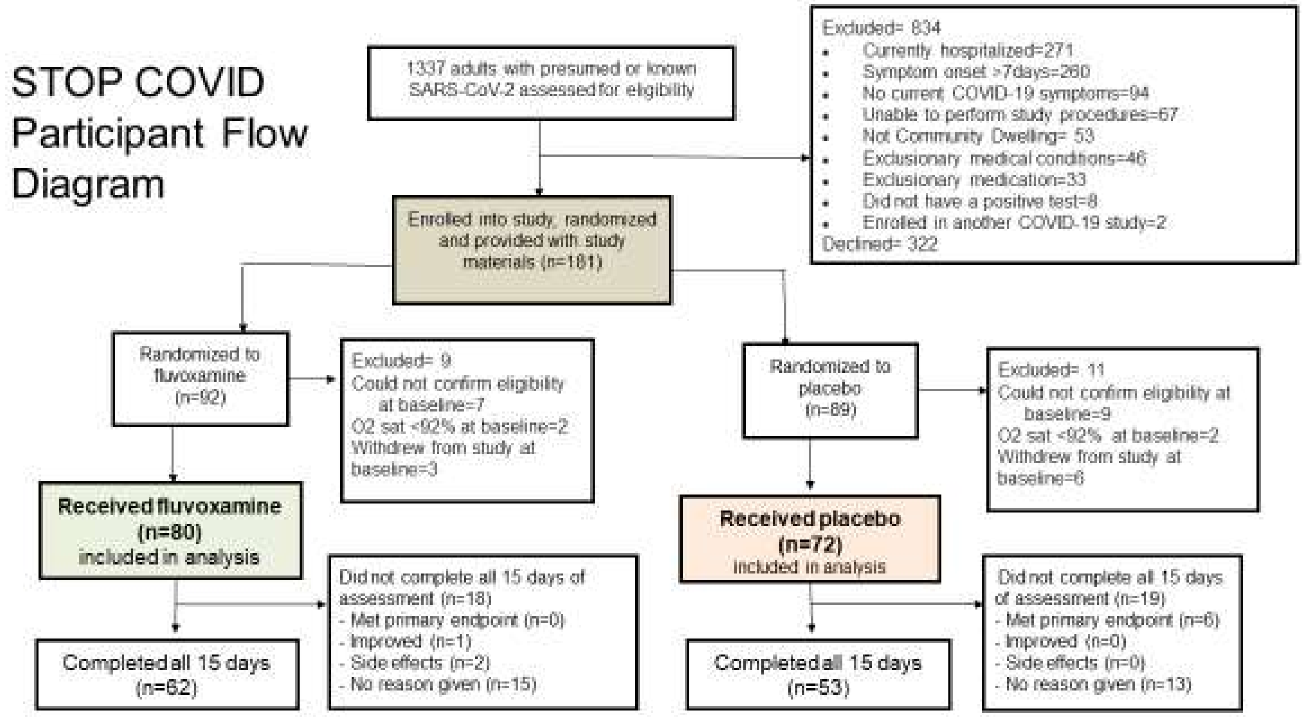

STOP COVID DEMOGRAPHICS

Characteristics	Fluvoxamine (n=80)	Placebo (n=72)
Sex at birth, No (%)		
Female	56 (70%)	53 (73.6%)
Male	24 (30%)	19 (26.4%)
Race, No (%)		
White/Caucasian	56 (70%)	50 (69.44%)
Black/African American	18 (23.1%)	20 (27.0%)
Asian	3 (3.9%)	1 (1.4%)
Other	2 (2.6%)	1 (1.4%)
Unknown	1 (1.3%)	0
American Indian/Alaska Native	0 (0%)	1 (1.4%)
Ethnicity, No (%)		
Non-Hispanic/Non-Latino	75 (93.8%)	66 (91.7%)
Hispanic/Latino	3 (3.9%)	2 (2.8%)
Unknown/not reported	2 (2.6%)	4 (5.4%)

STOP COVID BASELINE CHARACTERISTICS

Characteristics	Fluvoxamine (n=80)	Placebo (n=72)
Age(year)		
Median (IQR)	46 (35-58)	45 (36-54)
Range	20 – 75	21 – 69
Duration of COVID-19 symptoms*, days		
Median (IQR)	4 (3-5)	4 (3-5)
Range	1 - 7	1 - 7
Most severe COVID-19 symptom at baseline*, No (%)		
Loss of smell	26 (32.5%)	18 (25%)
Fatigue	17 (21.3%)	18 (25%)
Body aches	9 (11.3%)	13 (18.1%)
Cough	9 (11.3%)	1 (1.4%)
Subjective fever	8 (10%)	4 (5.6%)
Loss of appetite	3 (3.8%)	8 (11.1%)
Chills	3 (3.8%)	6 (8.3%)
Shortness of breath	2 (2.5%)	1 (1.4%)
Loss of taste	2 (2.5%)	2 (2.8%)
Nausea	1 (1.3%)	1 (1.4%)
Oxygen saturation		
Median (IQR)	97% (96-98%)	97% (96%-98%)
Range	93% - 99%	92% - 99%

The key finding from STOP COVID was that 0% (0/80) of fluvoxamine-treated individuals suffered clinical deterioration, vs. 8.3% (6/72) of those who received placebo (*Figure, right*). In this study, fluvoxamine was generally well-tolerated. The fluvoxamine group had 1 serious adverse event and 11 other adverse events, while the placebo group had 6 serious and 12 other adverse events. Further, we collected 30-day follow-up data (after the 15-day RCT phase): 1 individual in each group received ER or hospital care (1 placebo for chest pain, 1 fluvoxamine for headache) and no individuals clinically deteriorated. This demonstrated that fluvoxamine prevented, rather than merely delayed, deterioration.

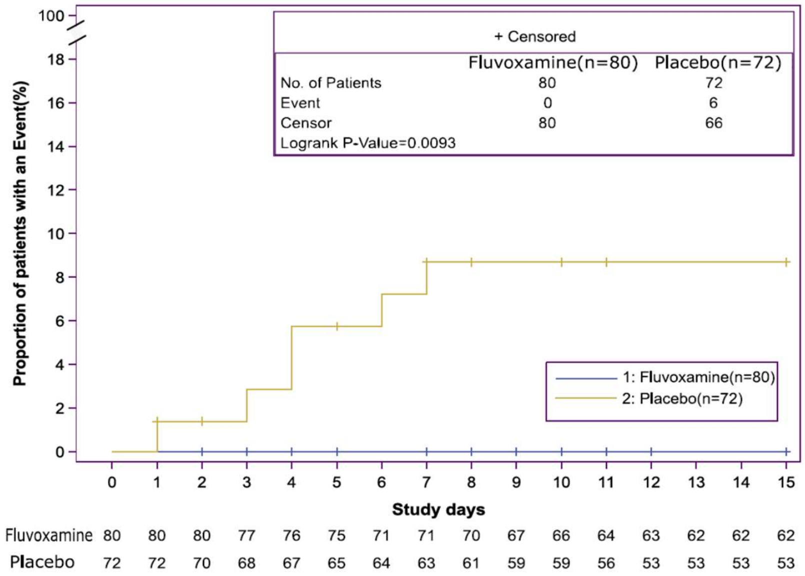

This preliminary study demonstrates the feasibility of conducting a fully-remote COVID-19 early treatment trial, with first dose of medication an average of 4 days after onset of symptoms. A fully-remote design has been demonstrated to be an efficient, pragmatic technique for conducting COVID-19 early treatment trials, as demonstrated by this study as well as the hydroxychloroquine studies led by University of Minnesota.

Baseline O_2 may be a moderator of fluvoxamine's treatment effect size. As the figure to the right shows, we found that low-normal baseline O_2 saturation may be a risk factor for clinical deterioration and a moderator of fluvoxamine's treatment effect size. In post-hoc analyses, we examined baseline (pre-treatment) O_2 saturations in the 152 participants. *All* of the clinical deterioration occurred in individuals with baseline O_2 saturation of ≤96%: 6/23 (26%), compared to 0/49 (0%) in those with baseline O_2 saturation ≥97%. This is important because it suggests that fluvoxamine could be given to individuals with early COVID-19 only if and when their O_2 saturation falls to 96% or less.

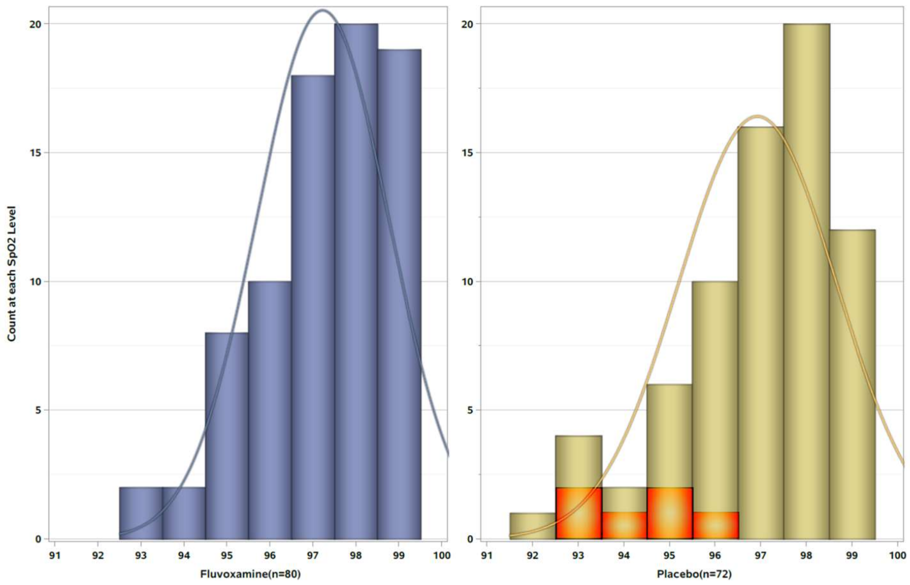

Anti-inflammatory (cytokine reduction) actions resulting from S1R activation would fit with recent findings of benefits of other anti-inflammatory drugs for COVID-19, including colchicine and corticosteroids.[17,18] However, a recent study found lower levels of cytokines in severe COVID-19 compared to bacterial sepsis.[19] If fluvoxamine's benefit is a S1R-mediated effect, this drug could be utilized not only to reduce morbidity and mortality in COVID-19, but also in other medical conditions in which treatments to dampen immunopathology are relevant – including future pandemics.

There were, however, several limitations to this study. First, this was a single-site study with a small sample size; the small sample size also results in wide confidence intervals (i.e., fluvoxamine appeared 100% effective at preventing clinical deterioration, but true effect size may be much lower than this). Second, we did not enrich the sample for risk factors for deterioration (such as older age); as a result, the rate of deterioration was low (8.3%) in the placebo group; this led to a fragile study finding.

Third, we attempted to measure symptoms of COVID-19 as a treatment outcome, by asking about most severe symptom at baseline and then measuring change in that symptom. This method proved to be invalid for several reasons. First, the presenting symptoms were highly heterogeneous as demonstrated in the Table above. Second, we conducted several advanced data analytic techniques focusing on latent trajectory modelling, and we found that the temporal dynamics of COVID-19 symptoms were highly heterogeneous. In other words, while symptoms overall decelerated (declined non-linearly over time), the trajectories of symptoms varied greatly between participants. This great heterogeneity of symptoms and their time course presents a challenge for the assessment of symptom change in clinical trials.[20] A validated measure of functional status may be more robust for assessing COVID-19 persistence; however, we did not include such a measure in STOP COVID. Additionally, a longer-term outcome is needed (beyond 15 days) to assess persistence of COVID-19.

Fluvoxamine Possible Mechanisms of Action

Fluvoxamine is a selective serotonin reuptake inhibitor (SSRI) and a S1R agonist.[21] The preliminary study was motivated by a hypothesis that S1R agonists could dampen excessive inflammation in COVID-19. This and other potential mechanisms by which fluvoxamine might improve outcomes in COVID-19 are summarized below.

1) Anti-inflammatory effects though the S1R-IRE pathway. The S1R is an endoplasmic reticulum (ER) chaperone protein involved in many cellular functions, including regulation of the ER stress response/unfolded protein response (UPR) and inflammation.[22] S1R has been shown to inhibit ER stress sensor inositol-requiring enzyme 1α (IRE1) mediated splicing of XBP1[23], a master regulator of cytokine production. These anti-inflammatory effects may be the most likely explanation for fluvoxamine's beneficial effects. In COVID-19, an excessive inflammatory process known as "cytokine storm" may contribute to worsening of symptoms and cardiopulmonary complications, which can sometimes occur around the second week of illness. Fluvoxamine may dampen this excessive inflammatory response.

In a 2019 study by Rosen and colleagues, fluvoxamine showed benefit in preclinical models of inflammation and sepsis.[10] In one model, mice were exposed to the Toll-like receptor 4 (TLR4) ligand lipopolysaccharide (LPS), which can trigger an inflammatory response. In another model, a fecal slurry was injected, which triggers a usually sub-lethal infection and an inflammatory response. Mice lacking the S1R showed excessive increases in cytokine levels and greatly reduced survival under either of these conditions, suggesting the S1R inhibits excessive inflammatory responses. Wildtype mice exposed to the same inflammatory triggers showed reduced cytokine levels and increased survival when treated with fluvoxamine (a S1R agonist). When investigating the underlying mechanism of this effect, the authors demonstrated that S1R inhibits activity of IRE1, which in turn prevents the excessive cytokine production. In an experiment using human peripheral blood, they also showed that fluvoxamine can reduce LPS-induced cytokine production by human cells. In the case of COVID-19, fluvoxamine's S1R agonist action may have a similar ability to reduce the excessive inflammatory response induced by the viral infection, thus reducing inflammation-mediated organ damage.

2) Anti-viral action through effects on lysosomes, autophagy and/or endocytosis. Coronaviruses utilize cathepsin proteases present in the late endosome to facilitate cell entry[24] and re-model ER and phagosomal membranes into sites of viral replication.[25] Both of these processes require stimulating the endocytosis and phagosome arms of autophagy and then terminating autophagy before lysosomal fusion. SARS-CoV-2 proteins Nsp6, Nsp2, Orf7b, and Orf9b have been shown to localize with and modulate components of the autophagy pathway.[26,27] Further Nsp6 has been shown to physically associate with S1R.[28] Critically, S1R not only drives early stage autophagy through the IRE1/UPR pathway, but is also essential for lysosomal fusion to complete autophagy, likely by chaperoning components of the SNARE complex.[29] We hypothesize activation of S1R with fluvoxamine may overcome Nsp6 inhibition of S1R to allow autophagy to clear SARS-CoV-2. Others have also recognized targeting the autophagy pathway as a promising strategy to treat SARS-CoV-2.[30,31]

Chemically, fluvoxamine is a cationic amphiphilic drug (CAD) with a log P 3.1 and a pKa 9.4 and, along with a range of antipsychotic and antihistamine drugs, preferentially accumulates in the lysosome. Perhaps due to this, fluvoxamine achieves higher concentrations in the lungs (which are lysosome-rich) than in the brain.[32] In the case of COVID-19, this may enhance treatment effects in the airway epithelium.[33] At high doses (10 uM), CADs including fluvoxamine, have

been shown to inhibit lysosomal acid sphingomyelinase[34] to cause drug-induced phospholipidosis. This non-specific activity can globally dysregulate lipid homeostasis, which in turn modulates autophagy through the mTOR nutrient sensing pathway.[35]

3) Antiviral effects and prevention of organ damage through regulation of the ER stress response/UPR. Some viruses hijack the ER stress response/UPR to achieve viral functions[36,37], and a number of studies have suggested drugs targeting the ER stress response/UPR may be beneficial in treating COVID-19.[28,38] S1R agonists (such as fluvoxamine) regulate ER stress. S1R ligand effects on ER stress and other ER functions may reduce organ dysfunction/damage.[39,40]

4) Antiplatelet Effects (common to all SSRIs). Hyperactivity of platelets may contribute to pathophysiological processes leading to thrombotic complications in COVID-19.[41] SSRIs can inhibit platelet activation, which may reduce the risk of thrombosis, and these anti-platelet effects may be cardioprotective.[42]

5) Elevation of melatonin levels in the body. The SARS-CoV-2 virus may activate the NLRP3 inflammasome[43], which may contribute to cytokine storm.[44] Melatonin may act on this NLRP3 pathway to reduce inflammation.[45,46] Fluvoxamine inhibits the metabolism of melatonin, so can increase the melatonin level in the body[47], which may be beneficial in COVID-19.

In summary: We need to replicate (or refute) these preliminary findings in a larger confirmatory trial. We need to find out: (1) can fluvoxamine prevent clinical deterioration in early COVID-19? (2) Can fluvoxamine mitigate the long-term impact of COVID-19? (4) Do the benefits of fluvoxamine for preventing deterioration vary as a function of baseline oxygen saturation?

2.3 RISK/BENEFIT ASSESSMENT

2.3.1 KNOWN POTENTIAL RISKS

Potential risks associated with study assessments

There are no risks associated with any of the assessments.

POTENTIAL RISKS ASSOCIATED WITH STUDY MEDICATION

Fluvoxamine:

General comments: Fluvoxamine is an antidepressant drug that functions as a selective serotonin reuptake inhibitor (e.g., similar to escitalopram (Lexapro), fluoxetine (Prozac), sertraline (Zoloft), etc – among the most commonly prescribed drugs in the US). Its risk profile below is for chronic use in a psychiatrically ill population; the risks for short-term use in a non-psychiatric population are likely lower, as our initial STOP COVID trial demonstrated (18 total AEs in 80 fluvoxamine-randomized participants, compared to 19 AEs in 72 placebo participants). The research team will carefully evaluate co-prescribed drugs as well as OTC medications and will instruct participants to minimize caffeine (which has its metabolism inhibited by fluvoxamine), to mitigate drug-drug interactions.

Likely risks: none.

Less likely (1-10%): Nausea, Vomiting, Weight loss, Yawning, Loss of appetite, Agitation/Nervousness/Anxiety, Insomnia, *Somnolence*, *Tremor*, Headache, Dizziness, *Palpitations*, *Tachycardia* (high heart rate), Abdominal pain,

Dyspepsia (indigestion), *Diarrhea*, *Constipation*, *Hyperhidrosis* (excess sweating), *Asthenia* (weakness), *Malaise*, *Sexual dysfunction* (including delayed ejaculation, erectile dysfunction, decreased libido, etc.), *Xerostomia* (dry mouth).

Rare (<1%): *Arthralgia*, Hallucination, Confusional state, Extrapyramidal side effects (e.g. dystonia, parkinsonism, tremor, etc.), *Orthostatic hypotension*, Cutaneous hypersensitivity reactions (e.g. edema [buildup of fluid in the tissues], rash, pruritus), *Mania* (elevated mood together with reduced need for sleep and increased energy), seizures, Abnormal hepatic (liver) function, Photosensitivity (being abnormally sensitive to light), Galactorrhea (expulsion of breast milk unrelated to pregnancy or breastfeeding).

Minimizing risk of fluvoxamine in this study:

Fluvoxamine has been used for the treatment of depression and anxiety disorders worldwide since the 1990s. Its safety profile is well-known from millions of patient-years of use. It is not fatal even in overdose[48] and it does not prolong the QTc interval unlike other SSRIs (especially citalopram). It requires no therapeutic drug level monitoring or safety labs for its use. Its use in this study is within FDA-approved dosing; and it is only used for approximately 15 days. As well, all participants receive an initial dose of 50mg, and based on tolerability with this initial dose and in the first day(s) of intervention, the dose can be adjusted as needed to minimize side effects. Further, monitoring will include oversight by physicians with expertise in this drug class. Study exclusions will minimize the risk of clinically significant drug:drug interactions or other problems.

Other risks

There may also be unknown risks**.** We will adjust the informed consent process if new risks are identified.

2.3.2 KNOWN POTENTIAL BENEFITS

Although the study hopes to prevent serious symptoms of COVID-19, there is no guarantee that the study participants will receive any clinical benefits by participating in the study. All participants regardless of study randomization, will receive increased monitoring of their COVID-19 illness, potentially leading to management (e.g., being told to contact their provider or go to the ER) that would not have occurred otherwise or as promptly without this monitoring.

Importance of the Knowledge to be Gained

COVID-19 is a US-wide and worldwide public health emergency. This study may result in effective treatment that reduces its morbidity.

2.3.3 ASSESSMENT OF POTENTIAL RISKS AND BENEFITS

RECRUITMENT AND CONSENT

All personnel involved in the design and conduct of the research involving human participants will receive the required education on the protection of human research participants prior to the start of this project. Procedures to recruit participants for the protocol and obtain their informed consent or assent are conducted and supervised by the PI. Trained study staff will discuss the study, including the risks and benefits of participation, with potential participants and relevant members of their treatment team as needed to provide informed consent for interested individuals. Informed consent will be obtained from all participants before any study procedures are initiated. The consent form, which incorporates HIPAA authorization, contains a description of the purpose and procedures, risks, procedures to minimize them, and possible benefits. Participants will be assured that participation in the study in completely voluntary, and that they are free to withdraw consent at any time and discontinue participation without prejudice to their current or future

medical care. The objectives of the project, all of the requirements for participation, and any possible discomforts and risks will be clearly explained to the participants orally and in writing. All participants must sign a consent document (e-consent or paper), indicating their consent, approved by the Washington University School of Medicine Institutional Review Board, before they can participate in the study.

PREVENTING BREACH OF CONFIDENTIALITY

Electronic records (computer files, electronic databases, etc.): The risks of breaching confidentiality will be strictly limited by the use of locked and restricted access to data, as well as the use of participant ID numbers rather than names in the survey database. All other data will be collected/entered in the secured, centralized REDCap database.

Paper/hard copy records (hard copy surveys, questionnaires, consent, etc.): The majority of data for this study will be collected and maintained electronically. For participants without access to internet, we will collect consent and data using paper documents. The risks of breaching confidentiality for paper documents will be strictly limited by the use of locked and restricted access to data, as well as the use of participant ID numbers rather than names whenever possible. Medical records containing PHI and research records are stored using the two-lock rule.

Data and Safety Monitoring Plan:

Serious adverse events (SAE) and adverse evets will be monitored continuously throughout the study and will be reported to the DSMB and IRB according to pre-specified requirements. SAE/AE rates and interim study results will be reviewed and discussed by the DSMB at the DSMB meetings.

3 OBJECTIVES AND ENDPOINTS

OBJECTIVES	ENDPOINTS	JUSTIFICATION FOR ENDPOINTS
Primary		
**(a)** Participants randomized to fluvoxamine will have a lower rate of clinical deterioration, compared to their respective placebos. **(b)** Participants with baseline O_2 saturation ≤96% will have a larger treatment effect size with fluvoxamine (i.e., larger fluvoxamine-placebo difference in rate of clinical deterioration) than those with O_2 saturation ≥97%.	**The primary outcome will be clinical deterioration**, defined as both of the following: (1) presence of dyspnea (SOB rating of 4+ on the twice daily 0-10 scale, and/or hospitalization for shortness of breath or pneumonia, plus (2) decrease in O_2 saturation (<92% on room air via the twice-daily at-home self-monitoring or via monitoring at a hospital/ER/urgent care center, and/or supplemental oxygen requirement to keep O_2 saturation ≥92%).<br><br>Two blinded research staff will adjudicate all potential cases of clinical deterioration to confirm these criteria are (or are not) met.	The goal of the study is to test prevention of clinical deterioration. We chose a clinical outcome including a laboratory-confirmed (ie SpO2) endpoint, consistent with World Health Organization clinical trial recommendations (https://www.who.int/blueprint/15-01-2020-nfr-bp-wg-clinical-trials-ncov.pdf?ua=1). In our previous trial (STOP COVID), 6 participants met this endpoint; 5/6 went to the hospital and 4/6 were hospitalized for an average of 10 days, demonstrating the clinical relevance of this endpoint. Although we saw no mortality and only 1 ventilator, mortality from COVID-19 is largely

OBJECTIVES	ENDPOINTS	JUSTIFICATION FOR ENDPOINTS
		due to a generalized inflammatory response with a deterioration in the respiratory status being the largest contributor to death. A focus on clinical deterioration from a respiratory standpoint, commonly observed in COVID-19 patients early on prior to ventilator need, would be in line with WHO's scale and would be consistent with an early deterioration observed in the clinical course of COVID-19 as described in the symptomatology literature<br><br>Regarding baseline O_2 saturation potential moderator of treatment effect on outcome, this is based on observations made in our preliminary study: all 6 cases of clinical deterioration (in the placebo group) were among those with baseline O_2 saturation 96% or below.
Secondary		
Participants randomized to fluvoxamine will have higher post-COVID functional status at day 15 and day 90, compared to placebo.	**Secondary outcome will be post-COVID functioning** at 15 days and at 90 days using the PROMIS Global Health Scale. This secondary outcome reflects the increasing concern regarding persistence of COVID-19 illness and functional impairment in a substantial percentage of individuals with initially "mild" symptoms. We will also explore persistent symptoms of COVID-19 at these timepoints.	It is increasingly clear that COVID-19 is a persistent illness for some. Measuring this persistence is challenging because the symptoms are highly heterogeneous and temporally dynamic. An alternative is to use a validated measure of functional status to assess whether fluvoxamine results in an increased level of function and quality of life. The 10-item PROMIS Global Health Scale is a validated measure of function and quality of life.
Tertiary/Exploratory		
We will also explore symptoms of COVID-19 at approximately day 15 and day 90.	We will ask each participant about symptoms of COVID-19 on a 0-10 scale for greatest severity ever of the symptom, and a 1-10 scale for current severity of the symptom.	The long-term course of symptoms of COVID-19 is still poorly understood. These data will provide additional information about COVID-19 symptoms and whether their persistence is influenced by the study medications.

4 STUDY DESIGN

4.1 OVERALL DESIGN

Summary: We propose a fully-remote US study: we will screen, consent, deliver fluvoxamine/placebo and other supplies to (self-quarantined) patients, including thermometers, blood pressure cuffs, and O_2 saturation monitors. We will collect data via participants' phones/computers and via medical records. We will carry out national recruitment, similar to the University of Minnesota COVID-19 hydroxychloroquine trials carried out in early 2020.

Hypotheses:

Primary:

Participants randomized to fluvoxamine will have a lower rate of clinical deterioration, compared to placebo.

Secondary:

Participants randomized to fluvoxamine will have higher post-COVID functional status at day 15 and day 90, compared to placebo.

Participants with baseline O_2 saturation ≤96% will have a larger treatment effect size (i.e., larger fluvoxamine-placebo difference in rate of clinical deterioration) than those with O_2 saturation ≥97%.

Phase: 3

Design: randomized, placebo-controlled, double-blinded, parallel design, nationally-recruiting, fully-remote, multi-site.

Methods to minimize bias: placebo control, blinding, randomization.

Interim analyses planned: none.

Stratification planned: sex, age.

Study team: The complementary expertise of our group will assure the success of this clinical trial, as already evidenced by the successful conduct of the preliminary trial supporting this application.

Eric Lenze, M.D., is a clinical trialist and geriatric psychiatrist. He has successfully conducted numerous clinical trials, including large-scale trials. He and his research team led the STOP COVID trial that randomized 152 individuals to fluvoxamine or placebo for early treatment of COVID-19 and demonstrated fluvoxamine's efficacy at preventing clinical deterioration. He will lead this clinical trial along with his study team, overseeing all aspects of the research.

Angela Reiersen, M.D., M.P.E., is a child psychiatrist and epidemiologist with experience conducting studies of comorbidities, longitudinal course, and genetics (including pharmacogenetics) of various childhood-onset psychiatric disorders and a rare genetic disorder of endoplasmic reticulum (ER) stress (Wolfram Syndrome). She developed the hypothesis regarding fluvoxamine's potential anti-inflammatory benefits in the treatment of COVID-19. Dr. Reiersen hypothesized the potential anti-inflammatory effect may occur through fluvoxamine's known actions on the S1R-Inositol-requireing enzyme 1α (IRE1) pathway, which is a component of the ER stress response. She initially became interested in S1R ligands years ago due to observed differences in anxiety/depression treatment response to SSRIs with varying S1R actions, which she saw among study participants with Wolfram Syndrome. Along with Dr. Lenze, she developed and oversaw the STOP COVID trial. Her role in the proposed trial will include study design (with emphasis on development of appropriate symptom questionnaires and other outcome measures), supervision of study staff, interpretation of findings based on knowledge of drug mechanisms, supervision of data analysis, and publication of results.

Caline Mattar, M.D., is an infectious disease physician and global health researcher. She helped design and oversee the STOP COVID trial. She will continue to advise the conduct of this new study and will help interpret, publish, and disseminate the results.

William Powderly, M.D., is an infectious disease physician. He is the Director of the Institute for Public Health at Washington University in St. Louis. He also directs the Institute for Clinical and Translational Sciences (ICTS) and is co-director of the Division of Infectious Diseases at the Washington University School of Medicine. He is an expert on the immune pathologies present in severe COVID-19. He will provide advice on the conduct of this study, and will help interpret, publish, and disseminate the results.

Phil Miller, biostatistician, has directed data management and analysis for numerous clinical trials, including many large-scale efforts, and has worked with Dr. Lenze for the past 5 years on several successful clinical trials, including STOP COVID.

Michael Avidan, M.D., is a critical care and anesthesiology specialist. He has successfully conducted several large pragmatic clinical trials in critical care populations, including the use of intra-operative ketamine to prevent post-operative delirium. He will provide advice on the conduct of this study, and will help interpret, publish, and disseminate the results.

John W. Newcomer M.D., is a psychiatrist and the President and CEO of South Florida Behavioral Health Network in Miami, Florida. He has previously a professor in psychiatry at Washington University in St. Louis. He will act as a consultant on this project.

Mike Rich, M.D., is a cardiologist at Washington University. He is known for his work on clinical trials, including studies of antidepressant medication in medically ill populations. He is also the PI of a new study on long-term post-COVID-19 functioning.

Jeffrey Rado, M.D., is a psychiatrist and internist at Northwestern University. He is a clinical trialist who will lead the study at the Northwestern site.

Rachel Hess, M.D., is an internist and PI of the CTSA at University of Utah. She is an experienced clinical trialist who has carried out COVID-19 trials.

Adam Spivak, M.D., is an infectious disease specialist at University of Utah. He and Dr. Hess will lead the University of Utah site for this trial.

Joshua Schiffer, M.D., is an infectious disease specialist at Fred Hutchinson Cancer Research Center in Seattle, Washington. He is an experienced COVID-19 clinical trialist.

Rachel Bender Ignacio, M.D., is an infectious disease specialist at Fred Hutchinson Cancer Research Center in Seattle, Washington. She is an experienced COVID-19 clinical trialist. She and Dr. Schiffer will lead the Fred Hutch site for this trial.

Sean Liu, M.D., Ph.D., is an infectious disease specialist at Icahn School of Medicine at Mount Sinai Hospital in New York, New York.

Internal consultant Charles Zorumski, M.D., is a neuroscientist and is chair of Psychiatry at Washington University. He has been a member of the STOP COVID team and has provided scientific direction and will continue to do so.

Consultant David Boulware, M.D., is an infectious disease specialist. He has successfully conducted fully-remote clinical trials with national (and Canada) recruitment, including the hydroxychloroquine early treatment trial which randomized 491 patients with early COVID-19 in approximately 6 weeks. Dr. Boulware and his team has been advising the investigators on the development of this new trial and will continue to do so, to ensure successful recruitment, screening and remote assessment and treatment of a large national sample.

Consultant Matthew Pullen, M.D., is an infectious disease specialist. He worked with Dr. Boulware as a key team member in the hydroxychloroquine study. He will work with this team to ensure successful recruitment, screening and remote assessment and treatment of a large national sample.

4.2 SCIENTIFIC RATIONALE FOR STUDY DESIGN

Rationale for study design: placebo control provides the highest level of rigor for efficacy and safety analyses. A placebo control is both necessary and safe/ethical because of the lack of a widely-available standard of care treatment for early COVID-19 illness.

4.3 JUSTIFICATION FOR DOSE

Fluvoxamine: Participants receive a day zero dose of 50mg fluvoxamine (or matching placebo) in the evening immediately after the baseline assessment and confirmation of eligibility, and 100mg twice daily as tolerated, on days 1-15. This dose range was determined based on fluvoxamine's binding affinity for the S1R. After 15 days, this study drug is stopped; there is no need to taper fluvoxamine (i.e., to prevent SSRI withdrawal) after only a brief course.

The dosing target of 200mg (100mg twice daily) in the proposed study is also justified by experience from the STOP COVID trial. That preliminary study used a target of 100mg three times daily based on the FDA maximum dose of 300mg/day, and based on a review of the S1R agonist activity at various doses of fluvoxamine (*https://pubmed.ncbi.nlm.nih.gov/17662961/*). However, in STOP COVID almost all participants (86/90; 96%) in the fluvoxamine group achieved a dose of 200mg/day, but only 50% increased up to 300mg/day and of those that did, this was only after 5-6 days' treatment, which may have already been outside the period of greatest risk for clinical deterioration. In other words, it did not appear necessary to reach 300mg/d. We also reviewed fluvoxamine's pharmacokinetics and binding affinity at the S1R with several experts in pharmacology, who agreed that a maximum of 200mg/d was sufficient for adequate binding of the S1R.

4.4 END OF STUDY DEFINITION

The end of the study is day 90, when the participant has completed the day 90 survey; or when the participant has withdrawn consent or was withdrawn by the PI.

5 STUDY POPULATION

5.1 INCLUSION CRITERIA

Inclusion criteria:

1) Men and woman age 30 and older;

2) Not currently hospitalized

3) Proven SARS-CoV-2 positive (per lab or physician report). Positive test is within 3 days prior to or during the current symptomatic episode.

Note: Test positivity can be via participant self-report initially; however, staff will also attempt to get confirmation (eg forwarded text from lab, photo of positive report, etc). For randomized participants for who there is no confirmation, a pre-planned secondary analysis of outcome will be conducted without them.

4) Currently symptomatic with one or more of the following symptoms: fever, cough, myalgia, mild dyspnea, chest pain, diarrhea, nausea, vomiting, anosmia (inability to smell), ageusia (inability to taste), sore throat, nasal congestion.

5) Expectation, on screening, that the participant will start study medication within 7 days of symptom onset.

> *Note: participants who are randomized and then fail to start study medication by day 7 of symptoms will (or may) remain in the study and be managed and assessed just as other randomized participants. They will not be included in the modified intention to treat sample during data analysis.*

5) Able to provide informed consent.

6) Upon initial screening, participant reports one of the following risk factors for clinical deterioration: age≥40, racial/ethnic group African-American, Hispanic, South Asian, or Native American (including more than one race), or 1+ of the following medical conditions which increase risk for developing moderate-severe COVID illness: obesity, hypertension, diabetes, heart disease (coronary artery disease, history of myocardial infarction, or heart failure), lung disease (eg asthma, COPD), immune disorder (eg rheumatoid arthritis, lupus).

5.2 EXCLUSION CRITERIA

Note: exclusions are same as in previous STOP COVID trial, but with additional exclusions for Coumadin and several other medications, based on recommendations of DSMB and co-investigators.

1) Illness severe enough to require hospitalization or already meeting study's primary endpoint for clinical worsening (eg current O_2 saturation <92% on room air, current use of supplemental oxygen to maintain O_2 saturation ≥92%).

> *Note: participants who are randomized and then found to meet clinical deterioration criteria at baseline – or meet deterioration criteria prior to starting study medication - will (or may) remain in the study and be managed and assessed just as other randomized participants. They will not be included in the modified intention to treat sample during data analysis.*

2) Unstable medical comorbidities (eg decompensated cirrhosis), per patient report and/or medical records.

3) Immunocompromised from the following: solid organ transplant, BMT, high dose steroids (>20mg prednisone per day), or tocilizumab

4) Already enrolled in another COVID 19 medication trial (not including vaccination or prophylaxis trials)

5) Unable to provide informed consent

6) Unable to perform the study procedures

7) Taking donepezil (rationale: donepezil is a S1R agonist), or sertraline (rationale: sertraline is a strong sigma-1 antagonist).

8) Taking warfarin-also known as Coumadin (rationale: increased risk of bleeding), phenytoin (rationale: fluvoxamine inhibits its metabolism), clopidogrel (rationale: fluvoxamine inhibits its metabolism from pro-drug to active drug which raises risk of cardiovascular events), and St John's wort (rationale: fluvoxamine + St John's wort are considered contraindicated because of the risk of serotonin syndrome)

9) Taking SSRIs, SNRIs, or tricyclic antidepressants, unless these are at a low dose such that a study investigator concludes that a clinically significant interaction with fluvoxamine (ie either serotonin syndrome or TCA overdose) is unlikely (examples: participant takes escitalopram but only at 5-10mg daily; that dose plus 200mg fluvoxamine would be insufficient to cause serotonin syndrome; or, participant takes amitriptyline but only at 25mg nightly; even if fluvoxamine inhibits its metabolism, it would be an insufficient dose to cause QTc prolongation or problematic side effects).

10) Individuals who report they have bipolar disorder or are taking medication for bipolar disorder (lithium, valproate, high-dose antipsychotic), unless the investigator concludes that the risk for mania is unlikely (ie it is doubtful that the patient actually has bipolar disorder).

11) Individuals who take alprazolam or diazepam and are unwilling to cut the medication by 25% (rationale: fluvoxamine modestly inhibits the metabolism of these drugs).

12) Participants taking theophylline, tizanidine, clozapine, or olanzapine (drugs with a narrow therapeutic index that are primarily metabolized by CYP 1A2, which is inhibited by fluvoxamine) will be reviewed with a study investigator and excluded unless the investigator concludes that the risk to the participant is low (this would be unlikely; example: participant takes tizanidine only as needed and is willing to avoid it for the 15 days of the study).

13) Received vaccine for COVID-19. Rationale: COVID-19 vaccines may prevent serious illness. Note that participants in vaccine trials are eligible, unless they know that they received the active vaccine.

14) Individuals who are already taking an approved or investigational COVID-19 treatment, or have already received monoclonal antibody treatment or convalescent plasma. (rationale: these individuals are unlikely to develop serious COVID-19).

5.3 SCREEN FAILURES

Screen failures are defined as participants who consent to participate in the clinical trial and subsequently randomly assigned to the study intervention but do not meet inclusion criteria prior to taking their first dose of study medication. Individuals who do not meet the criteria for participation in this trial (screen failure) will be rescreened if they subsequently meet inclusion criteria. The same subject number will be used.

5.4 STRATEGIES FOR RECRUITMENT AND RETENTION

This proposed remote clinical study will be conducted both locally through Washington University in St Louis, and nationally. The catchment reflects a diversity of settings including urban and rural settings, creating a diverse sample.

Study patients will be identified via a number of strategies, chiefly those shown effective in the fully-remote hydroxychloroquine studies: advertisements, media, and social media. As well, we will use strategies that were successful in the STOP COVID trial: using EHR, self-referral or through referrals from individual physicians. The research team, using interview with the potential participant and medical records review or discussion with the patient's physician, will then screen for eligibility as per the inclusion/exclusion criteria below.

Pre-screening and consent are conducted via REDCap surveys and telephone. No in person visits are required.

6 STUDY INTERVENTION

6.1 STUDY INTERVENTION(S) ADMINISTRATION

6.1.1 STUDY INTERVENTION DESCRIPTION

Participants are randomized to approximately 15 days of fluvoxamine or placebo. Study medications are matching oral capsules (taken twice daily). The study medication is stopped after approximately 15 days. The observation period for measuring the primary endpoint (clinical deterioration) is days 1-15 (beginning as soon as the first dose of the study medication and ending on day 15).

Sample size: We propose to enroll approximately n=1,100 participants, in order to have 880 in the modified intention to treat sample or analyzed sample. This sample will include all enrolled participants who were confirmed eligible at the baseline assessment and reporting taking at least one dose of study medication. A modified intention-to-treat strategy is required because participants are shipped supplies and study medication (whereupon they are randomized) prior to their measuring oxygen saturation to confirm study eligibility. In the preliminary study (STOP COVID), approximately 20% of individuals who received study supplies were not in the intention to treat sample, mainly because study eligibility could not be confirmed.

Randomization: Patients are randomized 1:1 to fluvoxamine or matching placebo capsules. A study statistician will generate randomization schedules stratified by age and sex. Treatments will be randomly allocated using alternating blocks of sizes 2 and 4. Randomization allocation is conducted via REDCap which displays randomization assignment to research team members who prepare study materials including drug or placebo but otherwise have no contact with participants. All individuals who are in contact with participants are blinded, including outcome assessors and investigators, as are participants themselves.

6.1.2 DOSING AND ADMINISTRATION

Participants receive a day zero dose of 50mg fluvoxamine (or matching placebo) in the evening immediately after the baseline assessment and confirmation of eligibility (as is feasible; sometimes this dose is not taken until the next day), and fluvoxamine 100mg (or placebo) twice daily as tolerated, on days 1-15. Fluvoxamine will be self-administered.

6.2 MEASURES TO MINIMIZE BIAS: RANDOMIZATION AND BLINDING

Participants and all study staff (except for the study statistician running the randomization, and the study staff putting study medication into the package to deliver to participant) are blinded. This blinding is kept throughout the study until analyses of the primary endpoint are preliminarily conducted.

Blocked randomization will also reduce the chance of bias. Another strategy to reduce bias is that the primary endpoint, clinical deterioration, includes an objective component, oxygen saturation, which may be less prone to an expectancy effect (vs. self-reported measures). Blinded study staff will not have access to randomization assignment for participants. Study investigators will also be blinded.

Treatment guess: Participants will make a treatment guess at the end of the 15-day double-blind RCT phase, as is standard for double-blinded trials to test the adequacy of the blind.

6.3 STUDY INTERVENTION COMPLIANCE

Participant data, including IP compliance, will be collected via REDCap daily surveys. Each day the survey will ask the participant how many fluvoxamine (vs placebo) capsules they took.

7 STUDY INTERVENTION DISCONTINUATION AND PARTICIPANT DISCONTINUATION/WITHDRAWAL

7.1 DISCONTINUATION OF STUDY INTERVENTION

Management of participants who clinically deteriorate and/or are hospitalized or visit an emergency room or urgent care: We will make every effort to continue the study intervention throughout the 15-day period if patients are not hospitalized, or if they are hospitalized but do not meet criteria for clinical deterioration. However, we will not exclude hospitalized participants from other physician-directed treatments (e.g., remdesivir, dexamethasone, convalescent plasma). For patients who are hospitalized and show deterioration, such patients will discontinue fluvoxamine/placebo, with the rationale that this study's main goal is test whether fluvoxamine prevents deterioration, and in such cases, the participant will have met this study's primary endpoint (i.e., the prevention has "failed"). They would be eligible for any studies of treatments for serious COVID. We will continue to collect data including day-15 and day-90 data under the intention-to-treat principle, as well as the rationale that fluvoxamine may improve longer-term outcomes even in cases that it fails to prevent hospitalization.56

Discontinuation from fluvoxamine or placebo does not mean discontinuation from the study, and remaining study procedures should be completed as indicated by the study protocol whenever possible. If a clinically significant finding is identified (including, but not limited to changes from baseline) after enrollment, the investigator or qualified designee will determine if any change in participant management is needed. Any new clinically relevant finding will be reported as an adverse event (AE).

The data that we will attempt to collect at the time of study intervention discontinuation will include the following:

- Vital signs, including O2 saturation, pulse, blood pressure and temperature.
- Hospitalization and supplemental oxygen status.

7.2 PARTICIPANT DISCONTINUATION/WITHDRAWAL FROM THE STUDY

Participants are free to withdraw from participation in the study at any time upon request.

The PI may discontinue or withdraw a participant from the study for the following reasons:

- Participant is not contributing data and/or appears to be lost to follow-up.
- If any clinical adverse event (AE), laboratory abnormality, or other medical condition or situation occurs such that continued participation in the study would not be in the best interest of the participant or interferes with the integrity of the study data.
- Disease progression which requires discontinuation of the study intervention
- If the participant meets an exclusion criterion (either newly developed or not previously recognized) that precludes further study participation

8 STUDY ASSESSMENTS AND PROCEDURES

8.1 EFFICACY ASSESSMENTS

Outcome Assessments: The primary endpoint will be clinical deterioration of COVID-19 illness. The secondary endpoint will be day 15 and day 90 functioning.

Clinical deterioration is defined meeting both of the following: (1) presence of dyspnea and/or hospitalization for shortness of breath or pneumonia, plus (2) decrease in O_2 saturation (<92%) on room air and/or supplemental oxygen requirement in order to keep O_2 saturation ≥92%.

The primary method of assessment for these outcomes is participants' reports on twice-daily surveys throughout the 15-day randomized period, corroborated by research staff (e.g., calling participants when low oxygen saturation was reported). For participants who stop answering the surveys prior to day 15, as well as those who appear to be meeting the primary endpoint, we will use, as is feasible, medical records and subsequent calls to these participants and their family to determine whether they sought medical care for shortness of breath or hypoxia. We will also confirm medical care needed (e.g., supplemental oxygen, length of stay, ventilator support) as is feasible.

We will collect sufficient data on all participants to also measure the clinical deterioration on the World Health Administration COVID-19 clinical trial outcome scale: 0-1: ambulatory (no clinical deterioration during RCT phase), 2: limitation of activities but no hospitalization; 3: hospitalization but no O_2 needed; 4: hospitalization, O_2 needed; 5: non-invasive ventilation or high-flow oxygen; 6: ventilator needed; 7: ventilator plus organ support needed; 8: death. Scale can be found on p6 of: *https://www.who.int/blueprint/priority-diseases/key-action/COVID-19_Treatment_Trial_Design_Master_Protocol_synopsis_Final_18022020.pdf.* Since ordinal scales have proved useful in studies of hospitalized patients with respiratory illnesses, this measure will be particularly helpful as an outcome measure for the subset of study participants who require hospitalization. This cross-walk with the WHO scale is also useful given that our primary outcome is a study-specific scale.

The secondary endpoint will be functioning at day 15 and day 90, measured by the 10-item PROMIS Global Health Scale ("Global-10"). This scale's items assess general domains of health and functioning including overall physical health, mental health, social health, pain, fatigue, and overall perceived quality of life. The 10 questions of the Global-10 have largely been adapted from older measures such as the *SF-36* and *EQ-5D*, with modifications that resulted in greater sensitivity and precision than the questions as originally worded.

We will also measure persistence of COVID-19 symptoms as an exploratory measure, asking about all symptoms that have been reported in the literature to date on a 0-10 scale (0= absent; 10=severe).

Data collection and participant interaction specifics: All interactions with participants will be fully-remote via phone/text/email/videoconference. Data collection is done by twice-daily REDCap surveys sent to patients via email, with telephone-based data collection as a back-up to ensure that individuals without internet access are able to participate. The surveys record oxygen saturation, vital signs, medication adherence, and COVID-19 symptoms. Fixed race and ethnicity categories are collected by interviewers as part of the demographic information to characterize the sample. The surveys measure dyspnea (shortness of breath) using a continuous 0-10 scale (0= symptom not present, 10= symptom is very severe) via the technique of Ecological Momentary Assessment (i.e., "how bad is your symptom right now?"). Phone contact is attempted daily during the first three days of the trial to address participants' questions and concerns such as medication-related issues, and throughout the trial to remind them to complete assessments as necessary. Additional phone calls are conducted on a case-by-case basis when participants' survey data indicate out of range values (e.g., low O_2 saturation) and to recheck and confirm these as necessary. This messaging will also be displayed when participants enter out of range values online for the baseline and daily surveys. Also, for participants who appear to be having worsening COVID-19 illness, study staff recommend that they seek medical attention from their primary care provider or via a local emergency room.

8.2 SAFETY AND OTHER ASSESSMENTS

The first assessment of safety will be conducted at screening. Research staff will, via telephone, determine whether participants meet eligibility criteria and what are their medical conditions and medications. This information will be

provided to the study physician investigator to concur with enrollment, prior to starting any study medication. For example, if participants are taking an SSRI or a medication of concern for interaction with fluvoxamine (e.g., olanzapine), this will need to be reviewed by the physician to determine whether the participant could safely participate in the study.

The second assessment of safety is conducted when study materials (including pulse oximeter, blood pressure cuff, and thermometer). Participants will report on their O2 saturation, vital signs, and current symptoms, via electronic survey, prior to starting study medication.

The third assessment of safety is throughout days 1-15 of the study. Participants will report on their O2 saturation, vital signs, symptoms, and any adverse events, via the twice-daily survey, with telephone corroboration with study staff as necessary.

All participants will, as is feasible, have a phone visit with study staff daily on days 1-3. Additional phone calls will be as needed (e.g., to confirm out of bounds values on O2 saturation or vitals, and to advise participants whether to call their provider or go to an emergency room).

Medical records, if available, will be used to supplement each of these assessments.

9 STATISTICAL CONSIDERATIONS

9.1 STATISTICAL HYPOTHESES

A formal Statistical Analysis Plan (SAP) will be completed prior to database lock and unblinding of the study data. It will include any additional details regarding the strategies described in this section.

The primary hypotheses (superiority hypotheses) are:

Participants randomized to fluvoxamine will have a lower rate of clinical deterioration, compared to placebo. (Null: participants randomized to fluvoxamine will not have a lower rate of clinical deterioration, compared to placebo)

- Primary Efficacy Endpoint:

Participants will meet the primary endpoint of clinical deterioration at the point that they meet both of the following: (1) presence of dyspnea and/or hospitalization for shortness of breath or pneumonia, plus (2) decrease in O_2 saturation (<92% on room air and/or supplemental oxygen requirement to keep O_2 saturation ≥92%). This will be measured during study days 1-15. The primary source of information to ascertain this endpoint is the twice-daily survey which is carried out on study days 1-15; this survey includes participant self-report of their shortness of breath (dyspnea) rating on a 0-10 scale (where a score of 4 or greater, indicating at least moderate dyspnea, meeting the first part of this endpoint) and of their pulse oximeter reading (where a reading of <92% meets the second part of this endpoint). Study staff will confirm the pulse oximeter readings with participants via phone when possible, asking them to re-check, and will call participants to collect these data via telephone if surveys have been skipped. Other data sources for this endpoint include participant and/or family report, or medical records data, regarding medical care received, during study days 1-15, in a hospital/ER/urgent care center.

The secondary hypotheses (also superiority hypotheses) are:

Participants randomized to fluvoxamine will have higher post-COVID functional status at day 15 and day 90, compared to placebo.

- Secondary Efficacy Endpoint(s):

Secondary outcome will be post-COVID functioning measured at 15 days and at 90 days post-randomization using the PROMIS 10-item Global Health Scale. The source of this data will be a survey sent to participants on these days, with data collection via telephone if the survey is skipped.

An exploratory analysis will examine outcomes of participants who are randomized but discovered to meet the study endpoint of clinical deterioration at baseline. We will examine whether those randomized to fluvoxamine have lower rates of hospitalization, ventilator care, and mortality, and we will gather the same outcomes at day 15 and day 90 post-randomization.

9.2 SAMPLE SIZE DETERMINATION

Software used for power calculations was power.prop.test in R.

With a sample size of 880 in the modified intention to treat sample, we will have 80% power to detect a treatment effect of 35% reduction (20% in placebo vs. 13% in fluvoxamine). Two-tailed alpha=0.05 for this power analysis, as time to clinical deterioration is the primary analysis and endpoint.

The basis for the assumption of 20% meeting the clinical deterioration endpoint (in the placebo group) is the enrichment strategy for this trial, in which participants must indicate, on screening, at least one risk factor for clinical deterioration (e.g., age, medical condition).

The basis for the assumption of 35% reduction: (1) 35% would be a clinically meaningful relative risk reduction in a high-risk group; (2) a 35% relative risk reduction is what has been reported with corticosteroids such as dexamethasone when given in more serious COVID-19.

To achieve n=880 in the modified intention to treat sample, we anticipate enrolling 1100 individuals, because we expect 20% dropout/ineligible from the time of shipping/couriering study supplies to the first dose of medication, as in the preliminary clinical trial. The main reason for dropout/ineligibility in the preliminary study was that we were unable to confirm study entry criteria, which included a baseline O_2 saturation of 92% or greater. This level of dropout/withdrawal after consent and shipping supplies is also consistent with the hydroxychloroquine early treatment trial conducted by University of Minnesota.

This sample size has suitable power for the secondary endpoint analysis (function at day 15 and day 90). For a simple t-test between two groups, n=400 each (allowing for dropout), we can detect a difference of 0.2 standard deviations (Fleiss' d) with a power of 80%; i.e., a small effect size.

The examination of persistent symptoms at day 15 and day 90 will be exploratory only; the analytic plan will depend on the structure of the data collected. Therefore no power analysis was conducted for this exploratory examination.

The moderator hypothesis is: Participants with baseline O_2 saturation ≤96% will have a larger treatment effect size (i.e., larger fluvoxamine-placebo difference in rate of clinical deterioration) than those with O_2 saturation ≥97%.

This moderator hypothesis is consistent with the strategy of prognostic enrichment, and our methodology and analytic strategy follows the FDA guidance on prognostic enrichment, found in *https://www.fda.gov/regulatory-information/search-fda-guidance-documents/enrichment-strategies-clinical-trials-support-approval-human-drugs-and-biological-products*.

The test for moderation is a test of the interaction between treatment and baseline O_2 saturation (dichotomized as ≤96% vs. ≥97%).

Power for this moderator hypothesis: We have at least 80% power at p=0.016 to find an effect size modulation such that in the low-risk group (baseline O_2 saturation ≥97%) there was a treatment odds ratio of 1 (e.g., equal proportions of

deterioration in both fluvoxamine and placebo groups), while in the high-risk group (baseline O_2 saturation≤96%) there was a treatment odds ratio of 0.4 (i.e., 12% deterioration in the fluvoxamine group vs. 26% in the placebo group).

This is not a power calculation for the moderation per se, but is an example of a moderation effect which could be detected. The odds ratios and the standard error of the log odds ratio are based on the usual formulas, e.g.
OR = *x11*x22)/(x21*x12)
SE(log(OR)) = sqrt(1/x11 + 1/x22 + 1/x21 + 1/x12)
as is a simple Z test of the difference between the odds ratios and its P value.

9.3 STATISTICAL ANALYSES

.

9.3.1 GENERAL APPROACH

- For descriptive statistics, categorical data will be presented as n (percentage), and continuous data will be presented as means with standard deviations, or median with interquartile range.
- For all inferential tests, p-value will be 0.05 with 95% confidence intervals reported, all two-tailed.
- Any covariates will be specified at the time of conducting analyses, but prior to unblinding.
- The primary analysis requires no checks for normality; the secondary endpoint analysis will require a check of normality and corrective procedures will be applied (e.g., transformation or nonparametric tests).
- We will use SAS 9.4 for analyses.

9.3.2 ANALYSIS OF THE PRIMARY EFFICACY ENDPOINT(S)

The primary analysis is survival analysis of the primary outcome (clinical deterioration). This analysis treats participants as censored on the day that they meet the primary outcome, or on the last day that they fill out an outcome assessment. It is superior to an exact test of proportions because of the ability to handle missing data (due to dropout or meeting endpoint).

Findings will be reported as the rates of clinical deterioration in groups (i.e., fluvoxamine vs. placebo), the absolute difference in the endpoint, the logrank chi square, and the respective p-value.

No imputation for missing data will be used. However, for all participants who do not complete surveys through day 15, we will use follow-up phone calls and medical records to ascertain whether they met the endpoint (e.g., were hospitalized and received supplemental oxygen).

9.3.3 ANALYSIS OF THE SECONDARY ENDPOINT(S)

Because of the potential for type 1 error due to multiple comparisons, findings for analyses of secondary endpoint (global function at day 15 and 90) should be interpreted as exploratory. The analyses will proceed regardless of findings of primary endpoint.

The measurement is the summary of the 10-item scale which is expected to have a normal distribution. It will be examined separately at days 15 and 90 rather than as a repeated measure. The scale is not measured at baseline, so the

comparison is a t-test or ANCOVA (if covariates are used) between intervention and placebo at these time points (day 15, day 90).

No imputation for missing data will be used.

10 SUPPORTING DOCUMENTATION AND OPERATIONAL CONSIDERATIONS

10.1 REGULATORY, ETHICAL, AND STUDY OVERSIGHT CONSIDERATIONS

10.1.1 FUTURE USE OF STORED SPECIMENS AND DATA

Data collected for this study will be analyzed and stored at Washington University. After the study is completed, the de-identified, archived data will be stored at Washington University for use by other researchers including those outside of the study. Permission to transmit data to other researchers will be included in the informed consent.

10.1.2 SAFETY OVERSIGHT

We will appoint DSMB members who are not involved in the study (i.e., as investigators). The board will include individuals with expertise important to this trial who will be responsible for overseeing the safety of participants and overseeing that the trial's conduct.

The DSMB will meet, at intervals determined by the DSMB, during the study (e.g., prior to start of recruitment, various times during recruitment phase, and as needed). They will review study processes such as screening rate, enrollment rate, follow-up rate, dropout rate, demographics of enrolled sample, baseline clinical data, and participant safety. The primary safety measure will be AE reports.

10.1.3 QUALITY ASSURANCE AND QUALITY CONTROL

Each clinical site will perform internal quality management of study conduct, data collection, documentation and completion.

Quality control (QC) procedures will be implemented beginning with the data entry system and data QC checks that will be run on the database will be generated. Any missing data or data anomalies will be communicated to the site(s) for clarification/resolution.

The investigational site will provide direct access to all trial related sites, source data/documents, and reports for the purpose of monitoring and auditing by the sponsor, and inspection by local and regulatory authorities.

11 REFERENCES

1. Wiersinga WJ, Rhodes A, Cheng AC, Peacock SJ, Prescott HC. Pathophysiology, Transmission, Diagnosis, and Treatment of Coronavirus Disease 2019 (COVID-19): A Review. *JAMA.* 2020;324(8):782-793.
2. Guan WJ, Ni ZY, Hu Y, et al. Clinical Characteristics of Coronavirus Disease 2019 in China. *N Engl J Med.* 2020;382(18):1708-1720.

3. Huang C, Wang Y, Li X, et al. Clinical features of patients infected with 2019 novel coronavirus in Wuhan, China. *Lancet.* 2020;395(10223):497-506.
4. Lescure FX, Bouadma L, Nguyen D, et al. Clinical and virological data of the first cases of COVID-19 in Europe: a case series. *Lancet Infect Dis.* 2020;20(6):697-706.
5. Mehta P, McAuley DF, Brown M, et al. COVID-19: consider cytokine storm syndromes and immunosuppression. *Lancet.* 2020;395(10229):1033-1034.
6. Prasad A, Prasad M. Single Virus Targeting Multiple Organs: What We Know and Where We Are Heading? *Front Med (Lausanne).* 2020;7:370.
7. Del Valle DM, Kim-Schulze S, Huang H-H, et al. An inflammatory cytokine signature predicts COVID-19 severity and survival. *Nat Med.* 2020;26(10):1636-1643.
8. Laing AG, Lorenc A, del Molino del Barrio I, et al. A dynamic COVID-19 immune signature includes associations with poor prognosis. *Nat Med.* 2020;26(10):1623-1635.
9. Rubin R. As Their Numbers Grow, COVID-19 “Long Haulers” Stump Experts. *JAMA.* 2020.
10. Rosen DA, Seki SM, Fernandez-Castaneda A, et al. Modulation of the sigma-1 receptor-IRE1 pathway is beneficial in preclinical models of inflammation and sepsis. *Sci Transl Med.* 2019;11(478).
11. Bourrie B, Bribes E, Derocq JM, Vidal H, Casellas P. Sigma receptor ligands: applications in inflammation and oncology. *Current opinion in investigational drugs (London, England : 2000).* 2004;5(11):1158-1163.
12. Lewis R, Li J, McCormick PJ, C LHH, Jeevaratnam K. Is the sigma-1 receptor a potential pharmacological target for cardiac pathologies? A systematic review. *International journal of cardiology Heart & vasculature.* 2020;26:100449.
13. Oxombre B, Lee-Chang C, Duhamel A, et al. High-affinity σ1 protein agonist reduces clinical and pathological signs of experimental autoimmune encephalomyelitis. *Br J Pharmacol.* 2015;172(7):1769-1782.
14. Hashimoto K. Activation of sigma-1 receptor chaperone in the treatment of neuropsychiatric diseases and its clinical implication. *J Pharmacol Sci.* 2015;127(1):6-9.
15. Cobos EJ, Entrena JM, Nieto FR, Cendan CM, Del Pozo E. Pharmacology and therapeutic potential of sigma(1) receptor ligands. *Curr Neuropharmacol.* 2008;6(4):344-366.
16. Hallifax D, Houston JB. Saturable Uptake of Lipophilic Amine Drugs into Isolated Hepatocytes: Mechanisms and Consequences for Quantitative Clearance Prediction. *Drug Metabolism and Disposition.* 2007;35(8):1325.
17. Deftereos SG, Giannopoulos G, Vrachatis DA, et al. Effect of Colchicine vs Standard Care on Cardiac and Inflammatory Biomarkers and Clinical Outcomes in Patients Hospitalized With Coronavirus Disease 2019: The GRECCO-19 Randomized Clinical Trial. *JAMA Network Open.* 2020;3(6):e2013136-e2013136.
18. Group TWREAfC-TW. Association Between Administration of Systemic Corticosteroids and Mortality Among Critically Ill Patients With COVID-19: A Meta-analysis. *JAMA.* 2020.
19. Kox M, Waalders NJB, Kooistra EJ, Gerretsen J, Pickkers P. Cytokine Levels in Critically Ill Patients With COVID-19 and Other Conditions. *JAMA.* 2020.
20. Mofsen AM, Rodebaugh TL, Nicol GE, Depp CA, Miller JP, Lenze EJ. When All Else Fails, Listen to the Patient: A Viewpoint on the Use of Ecological Momentary Assessment in Clinical Trials. *JMIR Ment Health.* 2019;6(5):e11845.
21. Narita N, Hashimoto K, Tomitaka S, Minabe Y. Interactions of selective serotonin reuptake inhibitors with subtypes of sigma receptors in rat brain. *Eur J Pharmacol.* 1996;307(1):117-119.
22. Delprat B, Crouzier L, Su TP, Maurice T. At the Crossing of ER Stress and MAMs: A Key Role of Sigma-1 Receptor? *Adv Exp Med Biol.* 2020;1131:699-718.
23. Mori T, Hayashi T, Hayashi E, Su TP. Sigma-1 receptor chaperone at the ER-mitochondrion interface mediates the mitochondrion-ER-nucleus signaling for cellular survival. *PLoS One.* 2013;8(10):e76941.
24. Hoffmann M, Kleine-Weber H, Schroeder S, et al. SARS-CoV-2 Cell Entry Depends on ACE2 and TMPRSS2 and Is Blocked by a Clinically Proven Protease Inhibitor. *Cell.* 2020;181(2):271-280 e278.
25. Knoops K, Kikkert M, Worm SH, et al. SARS-coronavirus replication is supported by a reticulovesicular network of modified endoplasmic reticulum. *PLoS Biol.* 2008;6(9):e226.

26. Gassen NC, Papies J, Bajaj T, et al. Analysis of SARS-CoV-2-controlled autophagy reveals spermidine, MK-2206, and niclosamide as putative antiviral therapeutics. *bioRxiv.* 2020:2020.2004.2015.997254.
27. Laurent EMN, Sofianatos Y, Komarova A, et al. Global BioID-based SARS-CoV-2 proteins proximal interactome unveils novel ties between viral polypeptides and host factors involved in multiple COVID19-associated mechanisms. *bioRxiv.* 2020:2020.2008.2028.272955.
28. Gordon DE, Jang GM, Bouhaddou M, et al. A SARS-CoV-2 protein interaction map reveals targets for drug repurposing. *Nature.* 2020;583(7816):459-468.
29. Yang H, Shen H, Li J, Guo LW. SIGMAR1/Sigma-1 receptor ablation impairs autophagosome clearance. *Autophagy.* 2019;15(9):1539-1557.
30. Gorshkov K, Chen CZ, Bostwick R, et al. The SARS-CoV-2 cytopathic effect is blocked with autophagy modulators. *bioRxiv : the preprint server for biology.* 2020:2020.2005.2016.091520.
31. Homolak J, Kodvanj I. Widely available lysosome targeting agents should be considered as potential therapy for COVID-19. *Int J Antimicrob Agents.* 2020;56(2):106044.
32. Daniel WA, Wojcikowski J. Contribution of lysosomal trapping to the total tissue uptake of psychotropic drugs. *Pharmacol Toxicol.* 1997;80(2):62-68.
33. Fung TS, Liu DX. The ER stress sensor IRE1 and MAP kinase ERK modulate autophagy induction in cells infected with coronavirus infectious bronchitis virus. *Virology.* 2019;533:34-44.
34. Kornhuber J, Tripal P, Gulbins E, Muehlbacher M. Functional inhibitors of acid sphingomyelinase (FIASMAs). *Handb Exp Pharmacol.* 2013(215):169-186.
35. Breiden B, Sandhoff K. Emerging mechanisms of drug-induced phospholipidosis. *Biol Chem.* 2019;401(1):31-46.
36. Chan SW. The unfolded protein response in virus infections. *Front Microbiol.* 2014;5:518.
37. Jheng JR, Ho JY, Horng JT. ER stress, autophagy, and RNA viruses. *Front Microbiol.* 2014;5:388.
38. Nabirotchkin S, Peluffo AE, Bouaziz J, Cohen D. Focusing on the Unfolded Protein Response and Autophagy Related Pathways to Reposition Common Approved Drugs against COVID-19. *Preprints.* 2020.
39. Hosszu A, Antal Z, Lenart L, et al. sigma1-Receptor Agonism Protects against Renal Ischemia-Reperfusion Injury. *J Am Soc Nephrol.* 2017;28(1):152-165.
40. Tagashira H, Bhuiyan MS, Fukunaga K. Diverse regulation of IP3 and ryanodine receptors by pentazocine through sigma1-receptor in cardiomyocytes. *Am J Physiol Heart Circ Physiol.* 2013;305(8):H1201-1212.
41. Manne BK, Denorme F, Middleton EA, et al. Platelet gene expression and function in patients with COVID-19. *Blood.* 2020;136(11):1317-1329.
42. Schlienger RG, Meier CR. Effect of selective serotonin reuptake inhibitors on platelet activation: can they prevent acute myocardial infarction? *Am J Cardiovasc Drugs.* 2003;3(3):149-162.
43. van den Berg DF, Te Velde AA. Severe COVID-19: NLRP3 Inflammasome Dysregulated. *Front Immunol.* 2020;11:1580.
44. Ratajczak MZ, Kucia M. SARS-CoV-2 infection and overactivation of Nlrp3 inflammasome as a trigger of cytokine "storm" and risk factor for damage of hematopoietic stem cells. *Leukemia.* 2020;34(7):1726-1729.
45. Garcia JA, Volt H, Venegas C, et al. Disruption of the NF-kappaB/NLRP3 connection by melatonin requires retinoid-related orphan receptor-alpha and blocks the septic response in mice. *FASEB J.* 2015;29(9):3863-3875.
46. Volt H, Garcia JA, Doerrier C, et al. Same molecule but different expression: aging and sepsis trigger NLRP3 inflammasome activation, a target of melatonin. *J Pineal Res.* 2016;60(2):193-205.
47. Hartter S, Wang X, Weigmann H, et al. Differential effects of fluvoxamine and other antidepressants on the biotransformation of melatonin. *J Clin Psychopharmacol.* 2001;21(2):167-174.
48. Henry JA. Overdose and safety with fluvoxamine. *Int Clin Psychopharmacol.* 1991;6 Suppl 3:41-45; discussion 45-47.
49. Lenze EJ, Mattar C, Zorumski CF, et al. Fluvoxamine vs Placebo and Clinical Deterioration in Outpatients With Symptomatic COVID-19: A randomized clinical trial. *JAMA. Published online November 12, 2020.* doi:10.1001/jama.2020.22760.

STOP COVID 2 Summary of Protocol Revisions

The initial protocol for the Stop Covid 2 study was approved by the Washington University's IRB on 12/10/2020. The final protocol revision was approved on 4/20/2021. Key protocol changes during the course of the study include:

- Added exclusion criteria #14: Individuals who are already taking an approved or investigational COVID-19 treatment, or have already received monoclonal antibody treatment or convalescent plasma.

- Added an exploratory analysis examining outcomes of participants who are randomized but discovered to meet the endpoint criteria at baseline.

- Modified exclusion criteria #3 by clarifying immunocompromised as from the following: solid organ transplant, BMT, high dose steroids (>20mg prednisone per day), or tocilizumab

- Modified inclusion criteria #6 to add racial category South Asian as a risk factor

- Increased the minimum age to enroll from 18 years old to 30 years old

- Added Icahn School of Medicine at Mount Sinai as a potential enrolling site (however, study enrollment ceased before this site gained final approval and activation).
